# Supplementary figures and images for: Diet-induced loss of adipose hexokinase 2 correlates with hyperglycemia
Source: eLife. 2023 Mar 15;12:e85103. doi: 10.7554/eLife.85103 (PMC10017106; doi:10.7554/eLife.85103)

Figure 1B vWAT

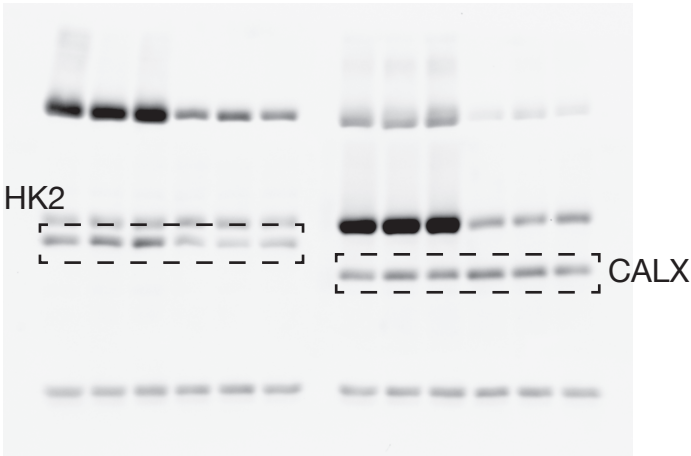

Figure 1B sWAT

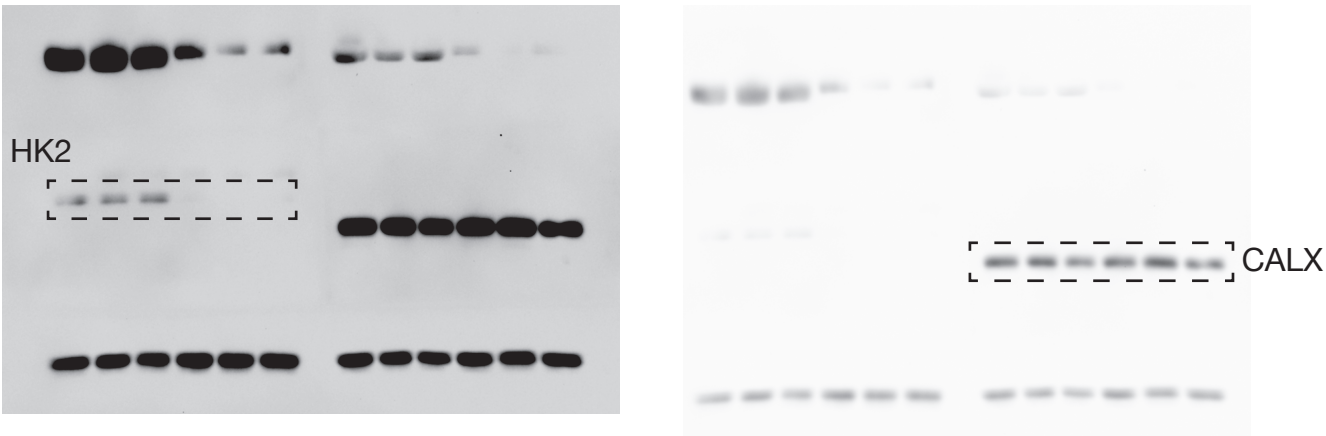

Figure 1B BAT

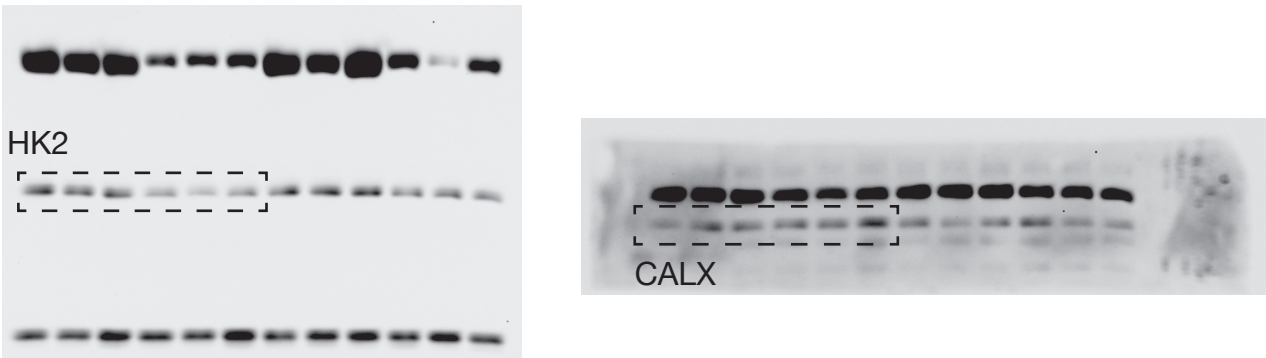

Figure 1B muscle

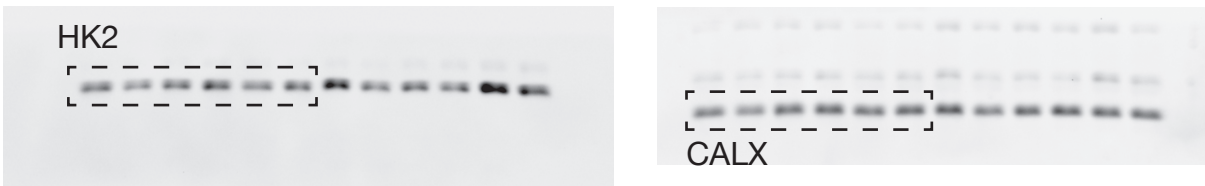

Supplement: Figure 1—source data 1. [file elife-85103-fig1-data1.zip › Figure 1 - source data/Figure 1B - source data.pdf]

Figure 1E vWAT

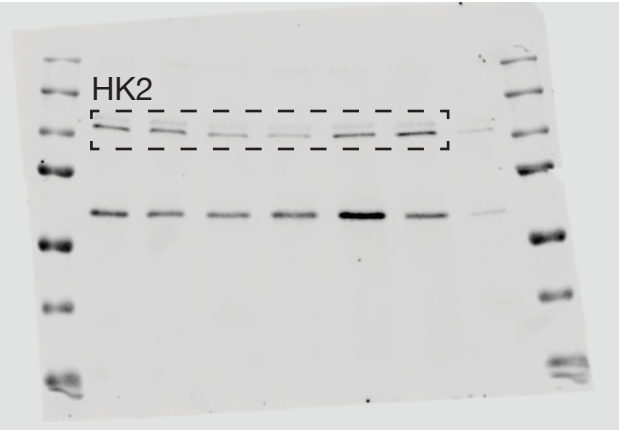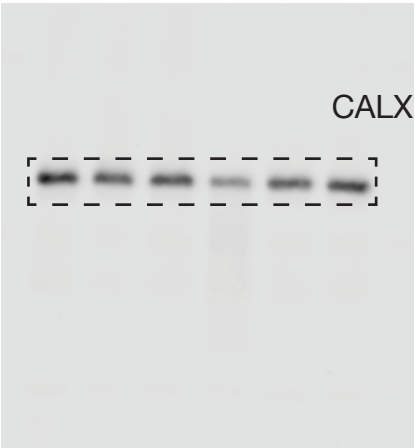

Figure 1F sWAT

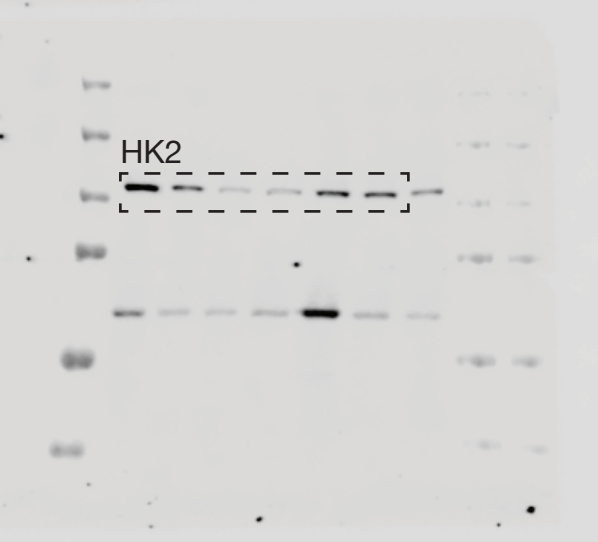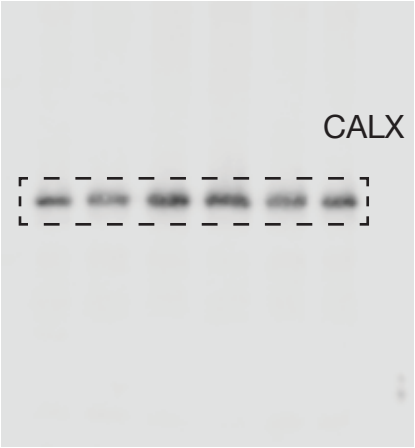

Supplement: Figure 1—source data 1. [file elife-85103-fig1-data1.zip › Figure 1 - source data/Figure1E-1F - source data.pdf]

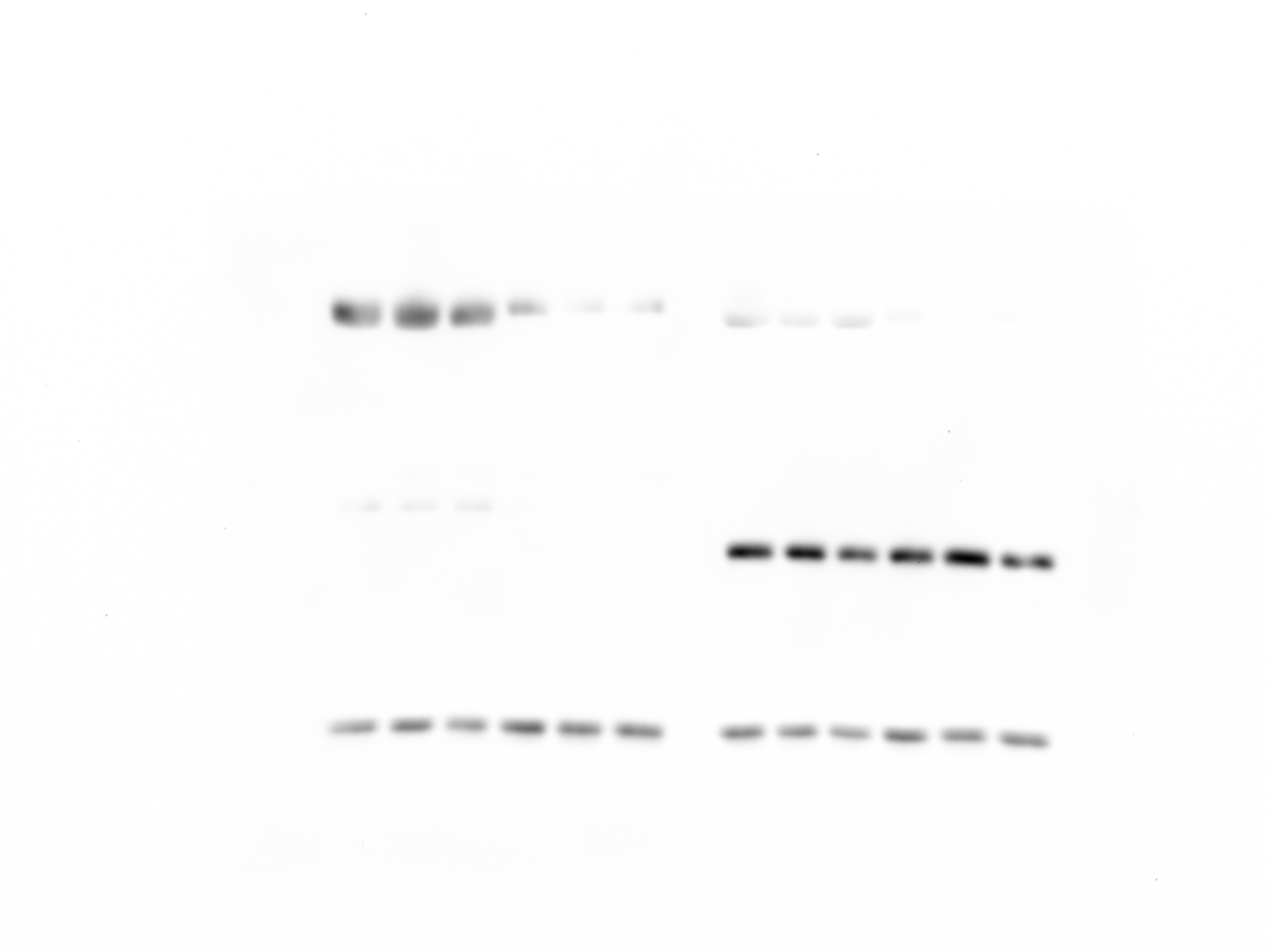

Supplement: Figure 1—source data 1. [file elife-85103-fig1-data1.zip › Figure 1 - source data/source data Figure1B/sWAT_HK2_CALX.tif]

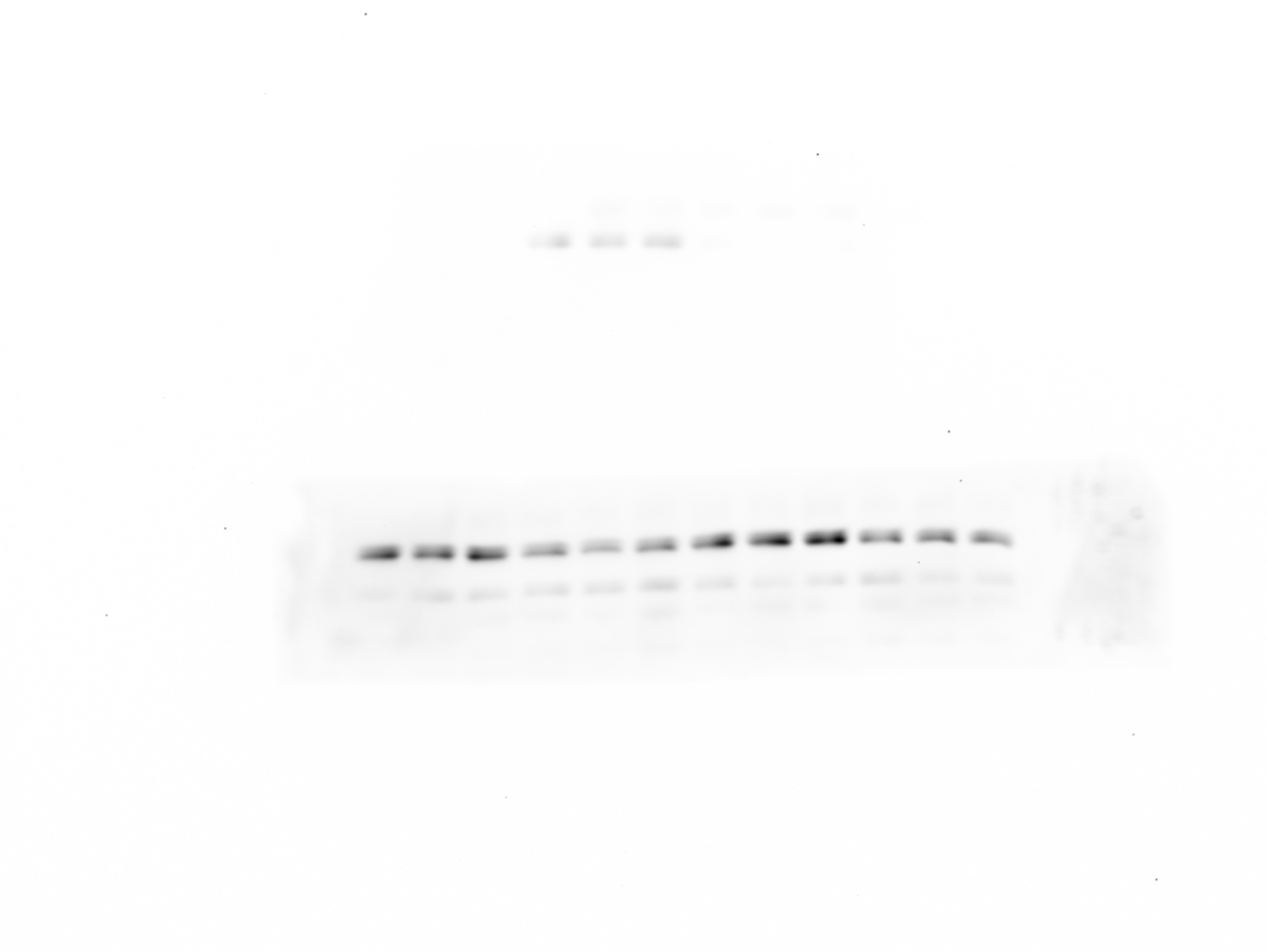

Supplement: Figure 1—source data 1. [file elife-85103-fig1-data1.zip › Figure 1 - source data/source data Figure1B/BAT_CALX.tif]

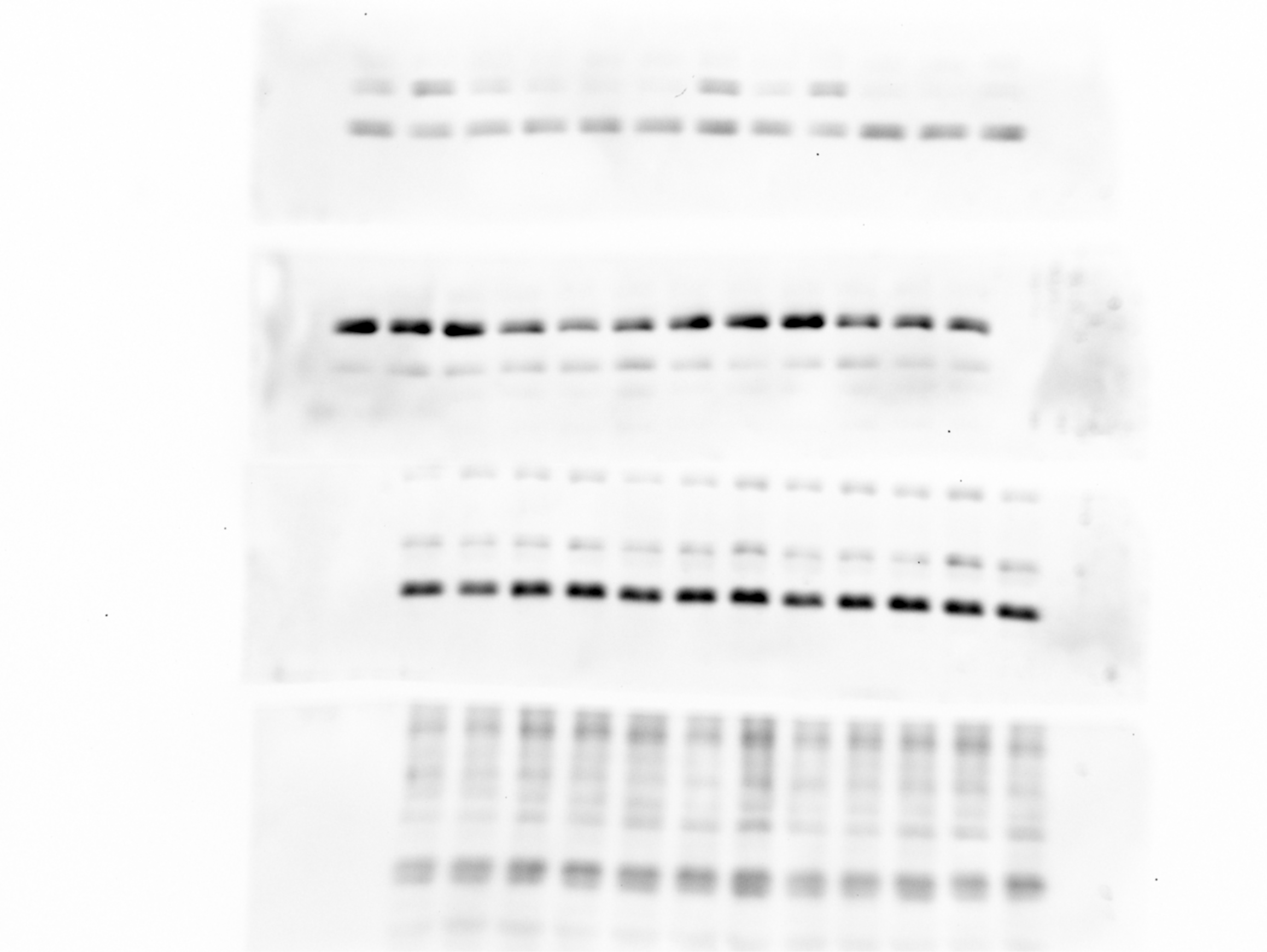

Supplement: Figure 1—source data 1. [file elife-85103-fig1-data1.zip › Figure 1 - source data/source data Figure1B/muscle_CALX.tif]

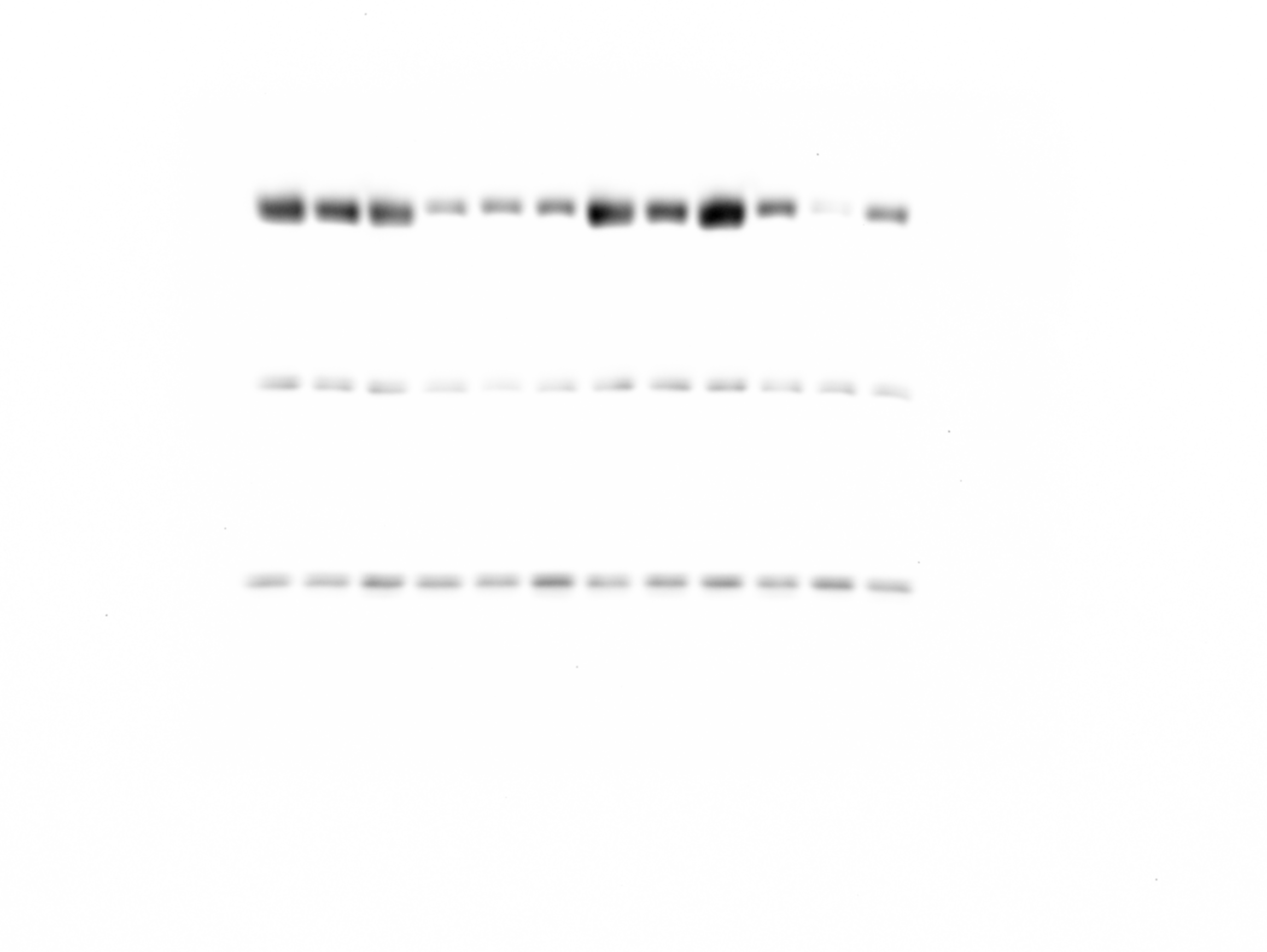

Supplement: Figure 1—source data 1. [file elife-85103-fig1-data1.zip › Figure 1 - source data/source data Figure1B/BAT_HK2.tif]

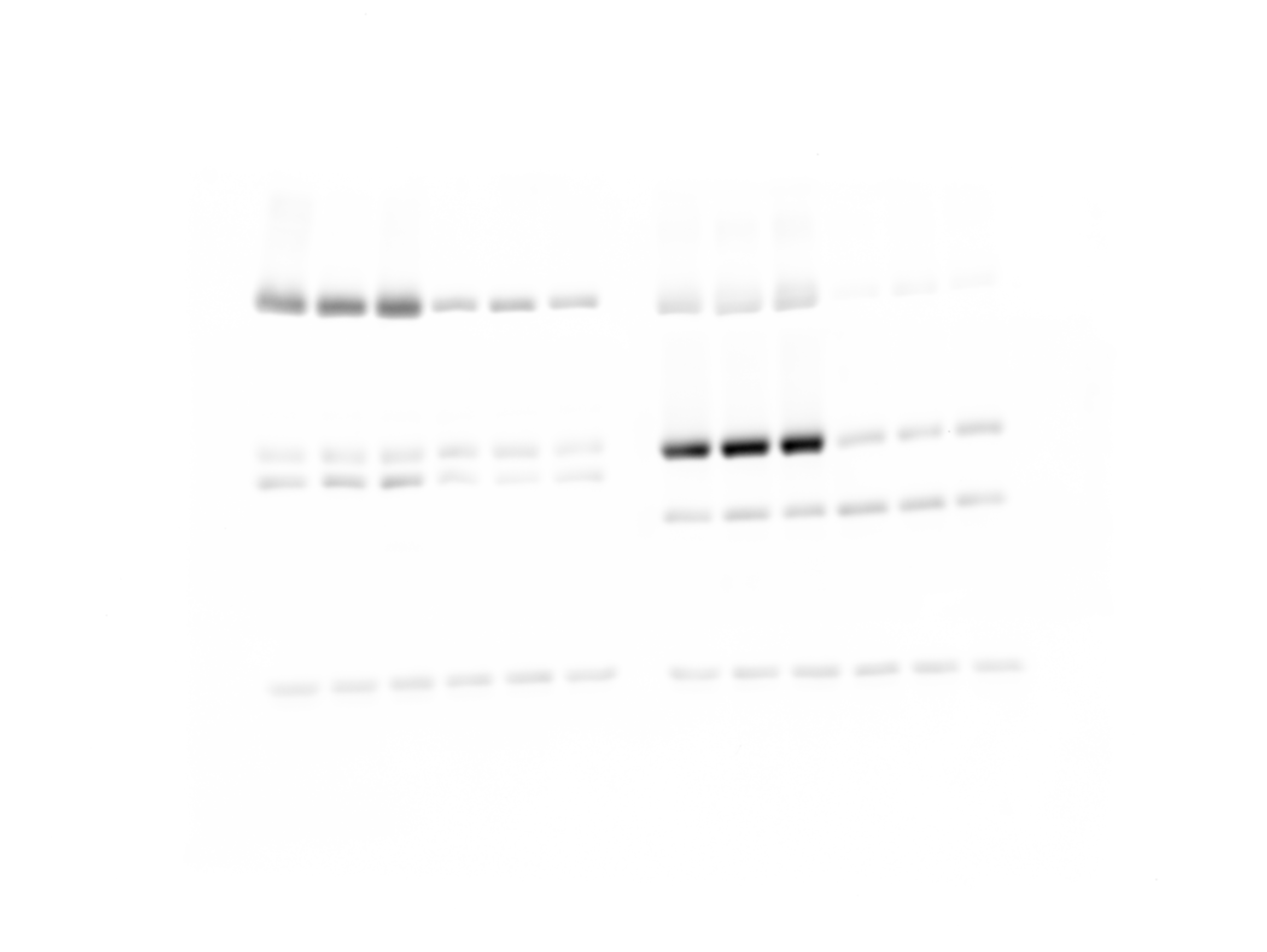

Supplement: Figure 1—source data 1. [file elife-85103-fig1-data1.zip › Figure 1 - source data/source data Figure1B/vWAT_HK2_CALX.tif]

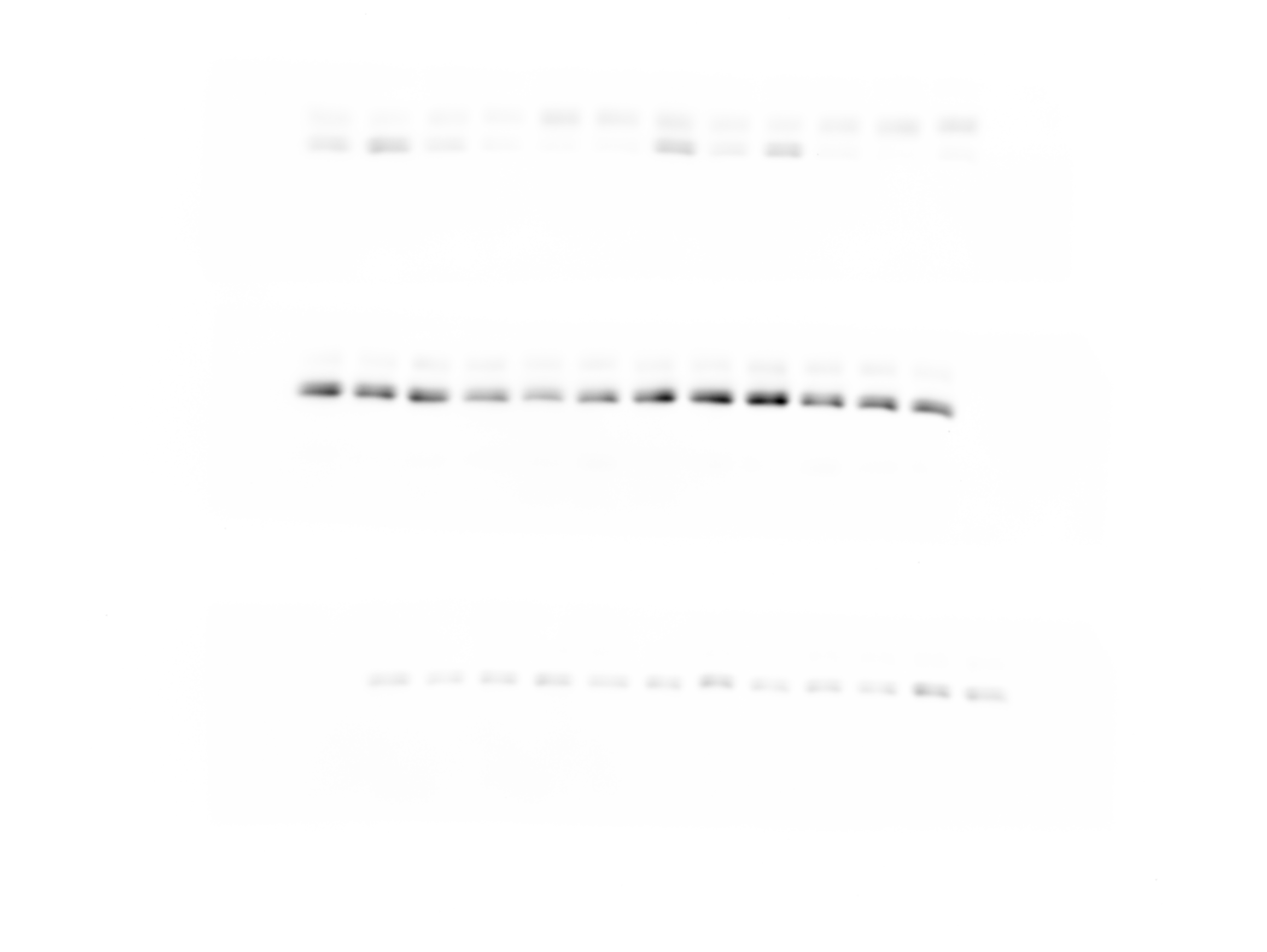

Supplement: Figure 1—source data 1. [file elife-85103-fig1-data1.zip › Figure 1 - source data/source data Figure1B/muscle_HK2.tif]

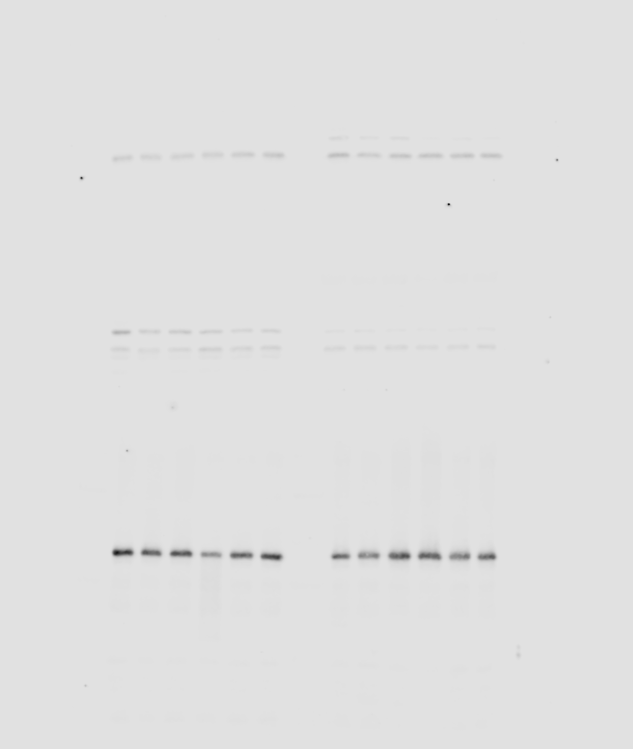

Supplement: Figure 1—source data 1. [file elife-85103-fig1-data1.zip › Figure 1 - source data/source data Figure1E-1F/eWAT_sWAT_CALX.tif]

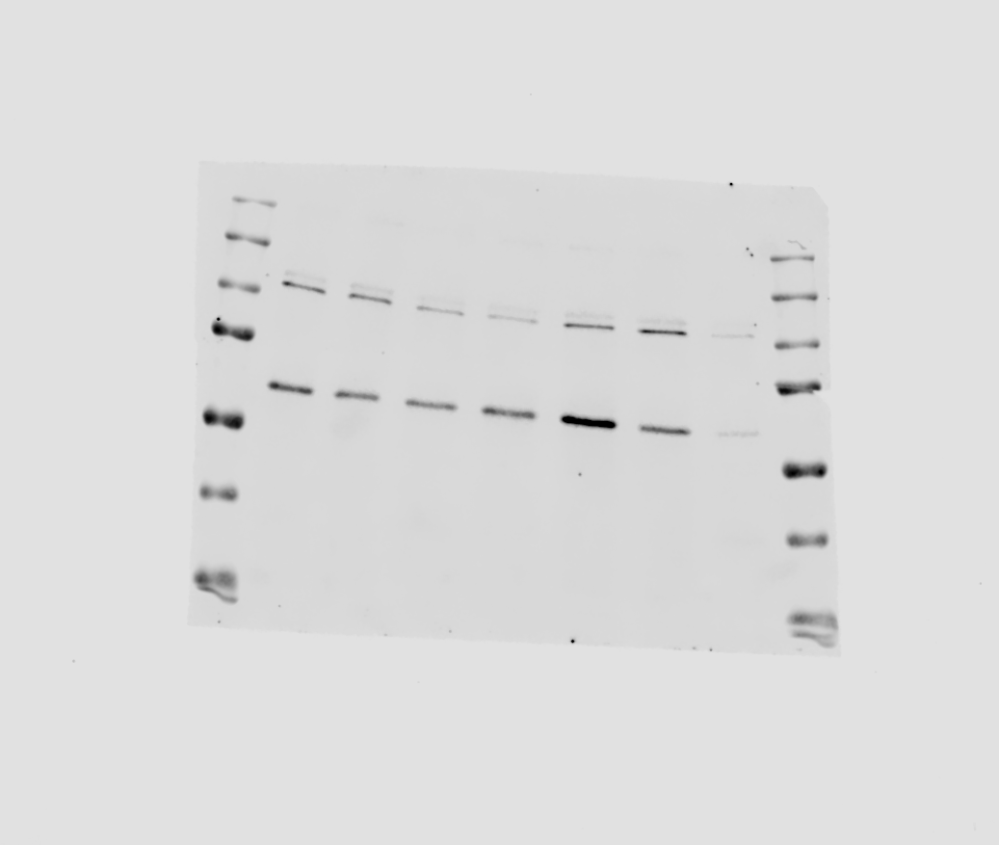

Supplement: Figure 1—source data 1. [file elife-85103-fig1-data1.zip › Figure 1 - source data/source data Figure1E-1F/eWAT_HK2.tif]

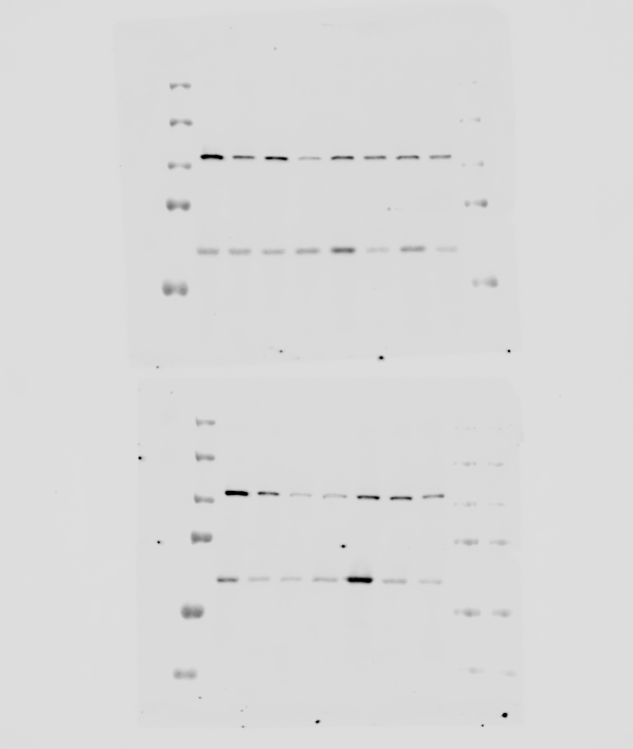

Supplement: Figure 1—source data 1. [file elife-85103-fig1-data1.zip › Figure 1 - source data/source data Figure1E-1F/sWAT_HK2.tif]

Figure 1 - figure supplement 1A

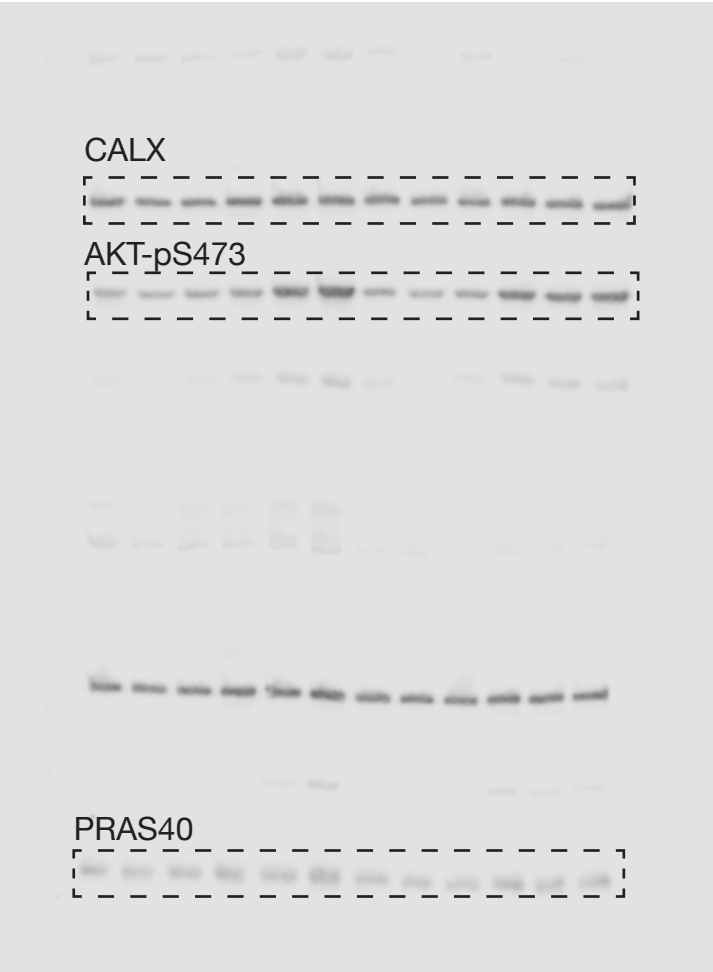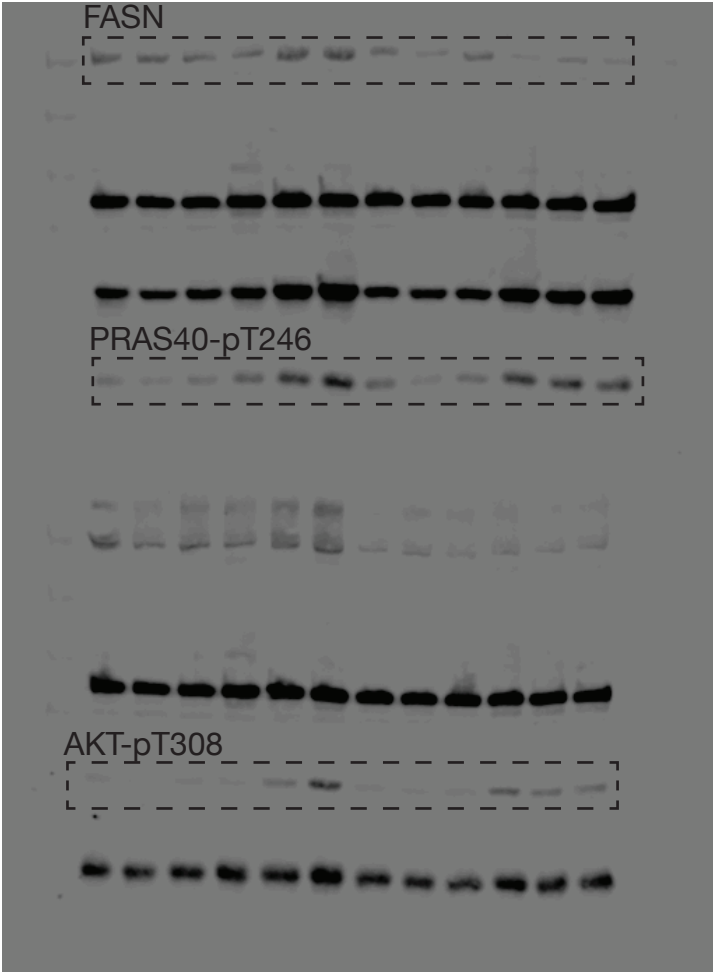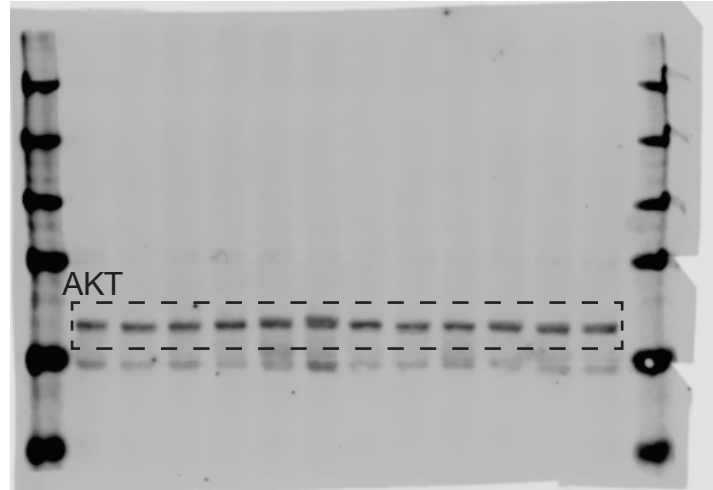

Supplement: Figure 1—figure supplement 1—source data 1. [file elife-85103-fig1-figsupp1-data1.zip › Figure 1 - figure supplement 1 - source data/Figure 1 - figure supplement 1A.pdf]

Figure 1 - figure supplement 1F vWAT

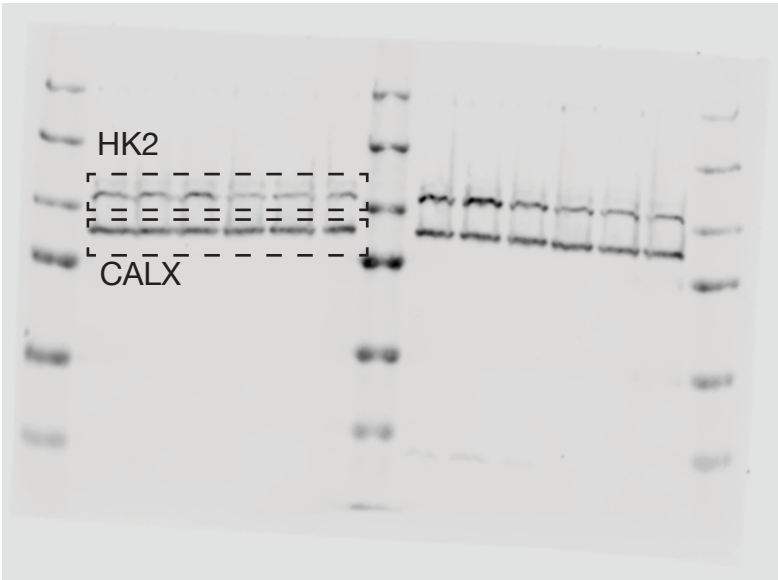

Figure 1 - figure supplement 1F sWAT

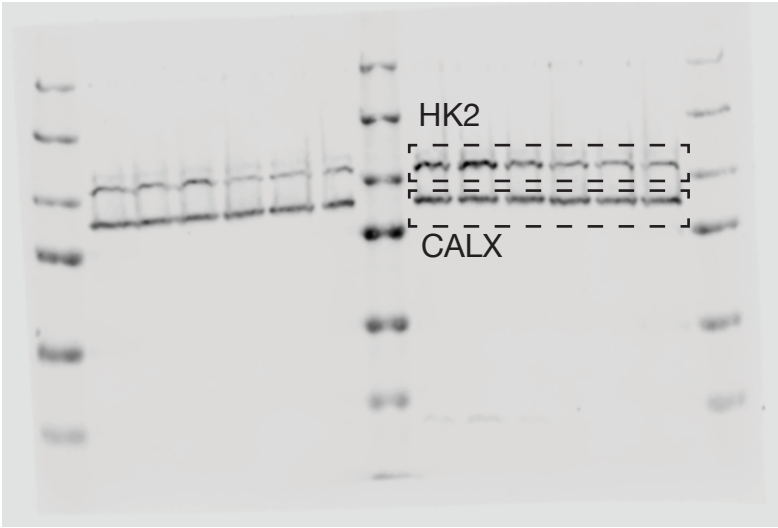

Supplement: Figure 1—figure supplement 1—source data 1. [file elife-85103-fig1-figsupp1-data1.zip › Figure 1 - figure supplement 1 - source data/Figure 1 - figure supplement 1F.pdf]

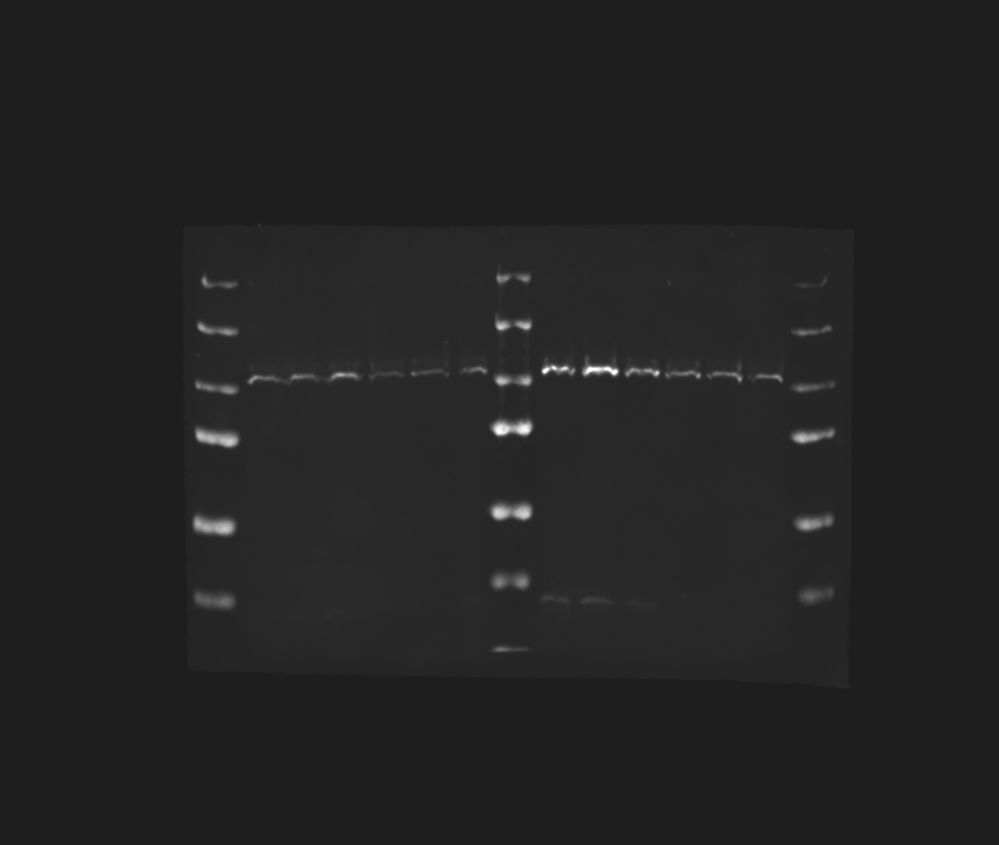

Supplement: Figure 1—figure supplement 1—source data 1. [file elife-85103-fig1-figsupp1-data1.zip › Figure 1 - figure supplement 1 - source data/source data Figure 1 - figure supplement 1F/eWAT_sWAT_HK2.tif]

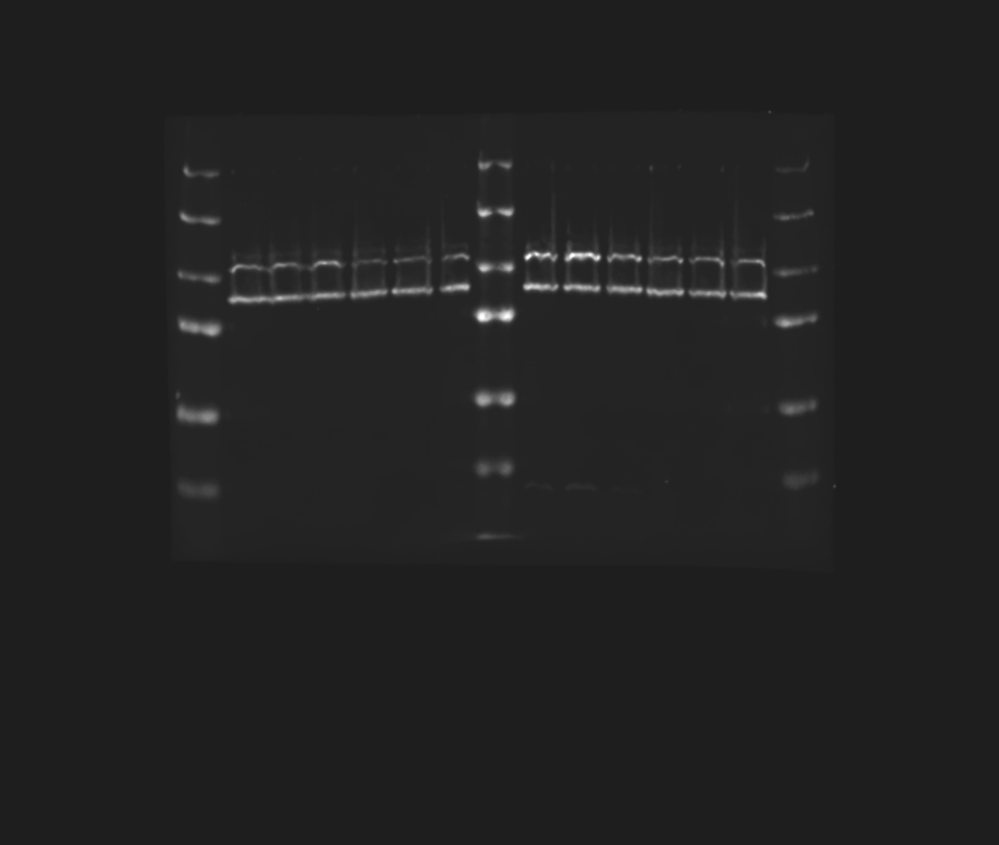

Supplement: Figure 1—figure supplement 1—source data 1. [file elife-85103-fig1-figsupp1-data1.zip › Figure 1 - figure supplement 1 - source data/source data Figure 1 - figure supplement 1F/eWAT_sWAT_HK2_CALX.tif]

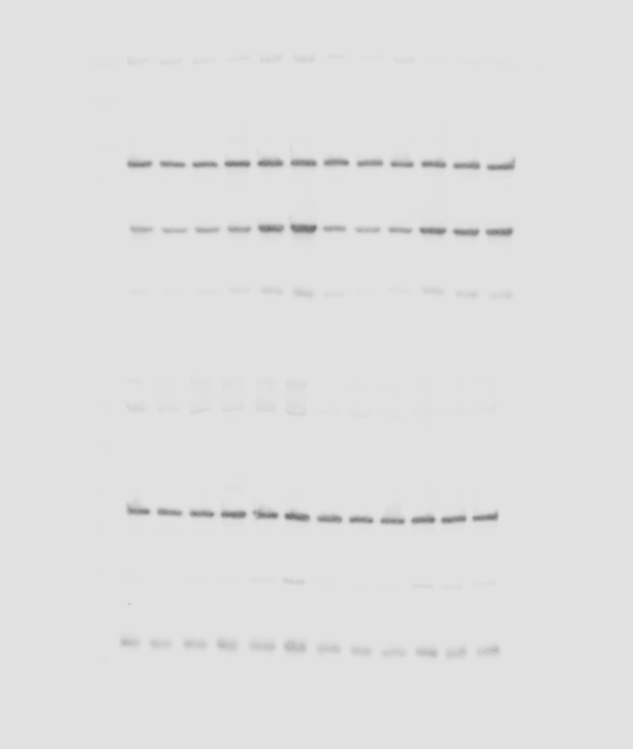

Supplement: Figure 1—figure supplement 1—source data 1. [file elife-85103-fig1-figsupp1-data1.zip › Figure 1 - figure supplement 1 - source data/source data Figure 1 - figure supplement 1A/FASN_CALX_pS473_pPRAS40_ACC_pT308_PRAS40.tif]

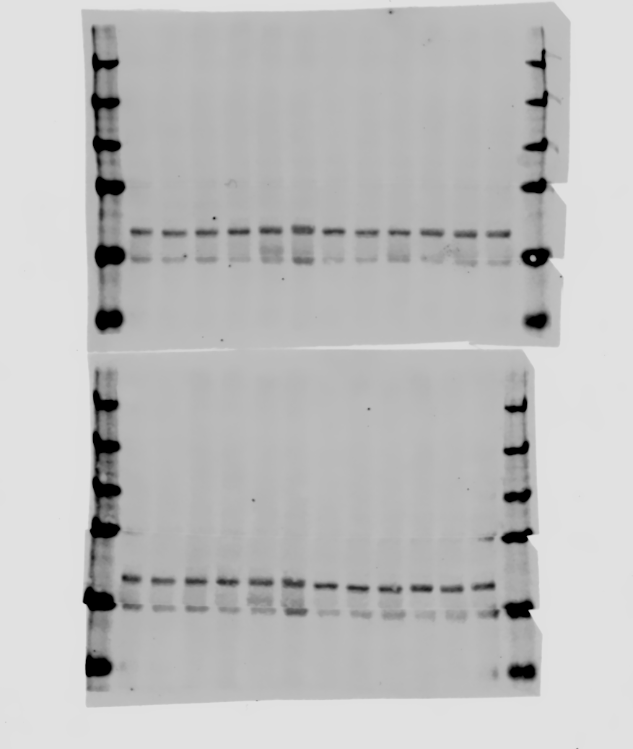

Supplement: Figure 1—figure supplement 1—source data 1. [file elife-85103-fig1-figsupp1-data1.zip › Figure 1 - figure supplement 1 - source data/source data Figure 1 - figure supplement 1A/AKT.tif]

Figure 1 - figure supplement 2D

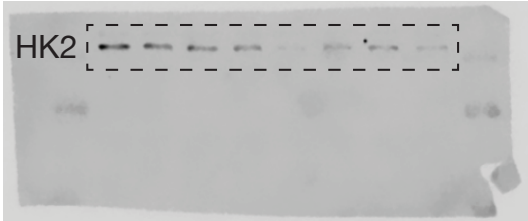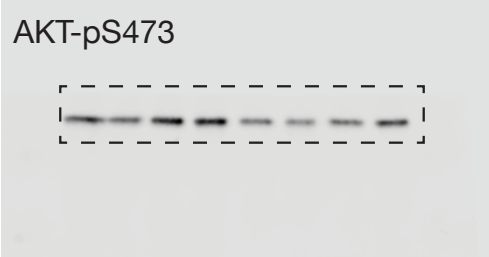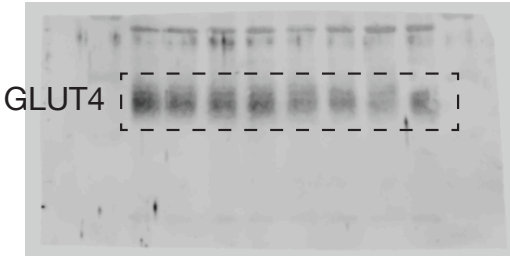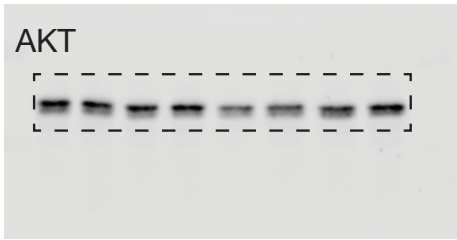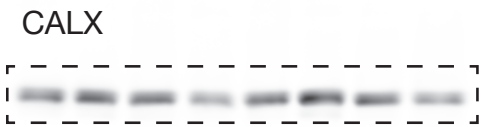

Figure 1 - figure supplement 2E

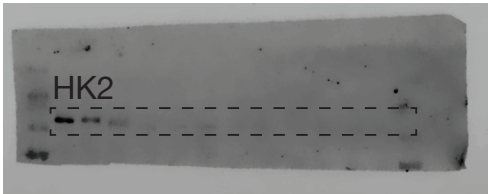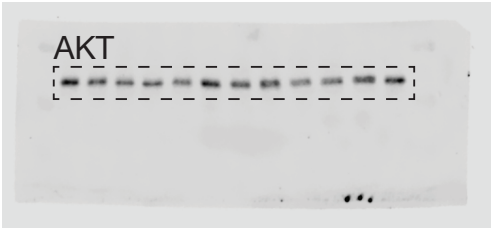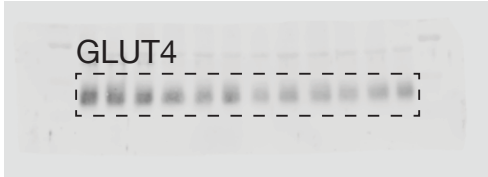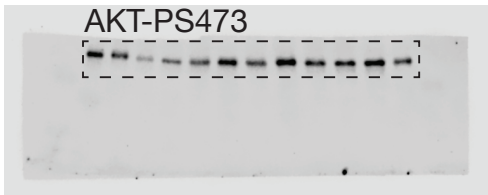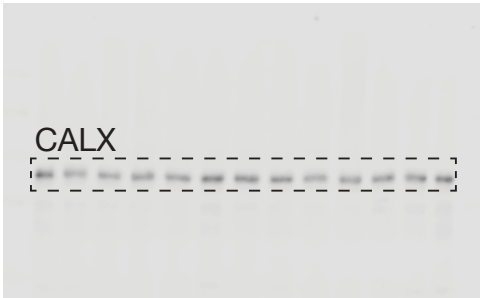

Figure 1 - figure supplement 2F

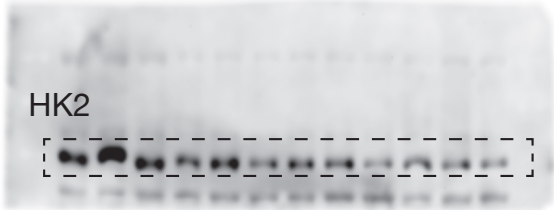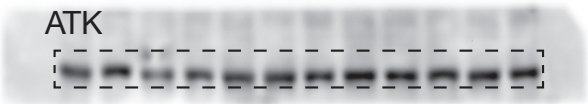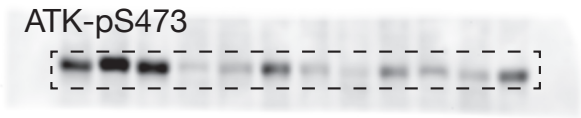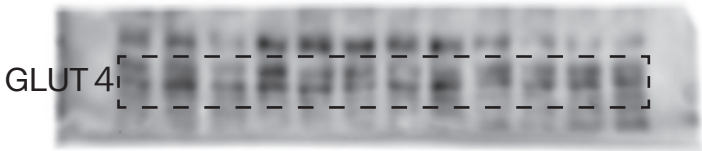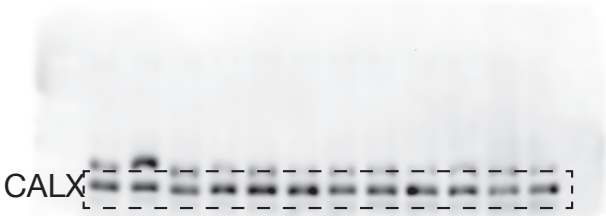

Supplement: Figure 1—figure supplement 2—source data 1. [file elife-85103-fig1-figsupp2-data1.zip › Figure 1 - figure supplement 2 - source data/Figure 1 figure supplment 2D-F.pdf]

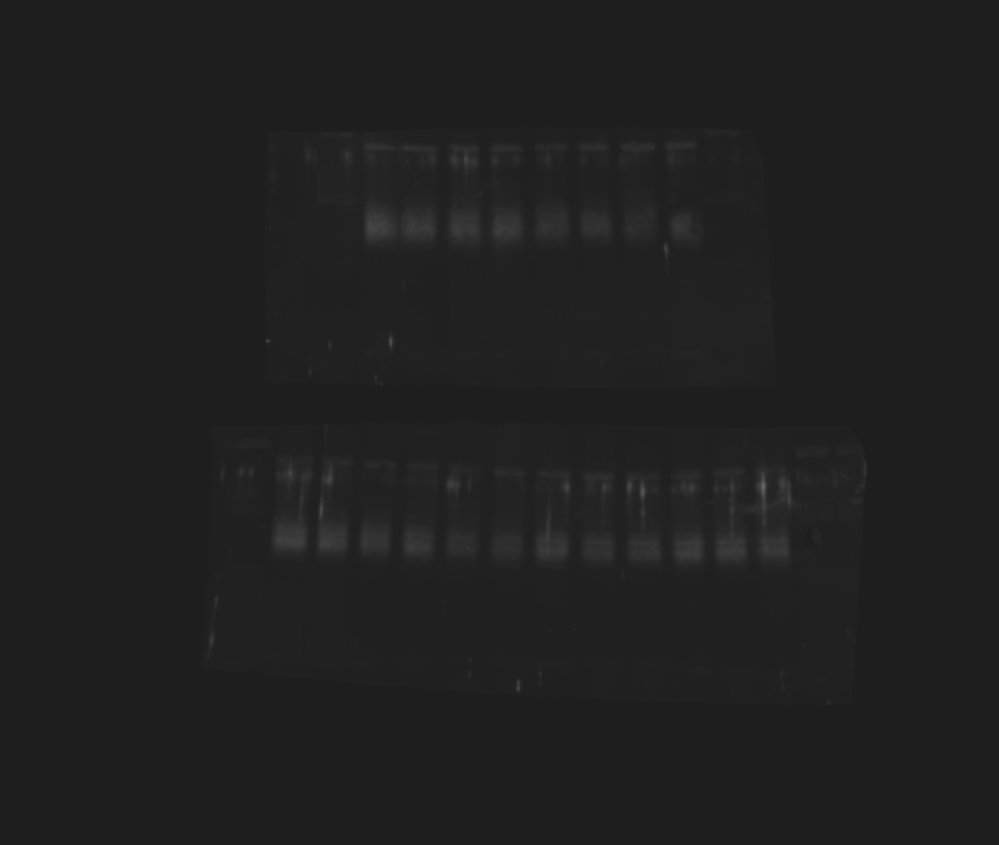

Supplement: Figure 1—figure supplement 2—source data 1. [file elife-85103-fig1-figsupp2-data1.zip › Figure 1 - figure supplement 2 - source data/source data Figure 1 - figure supplement 2D-F/vWAT_GLUT4.tif]

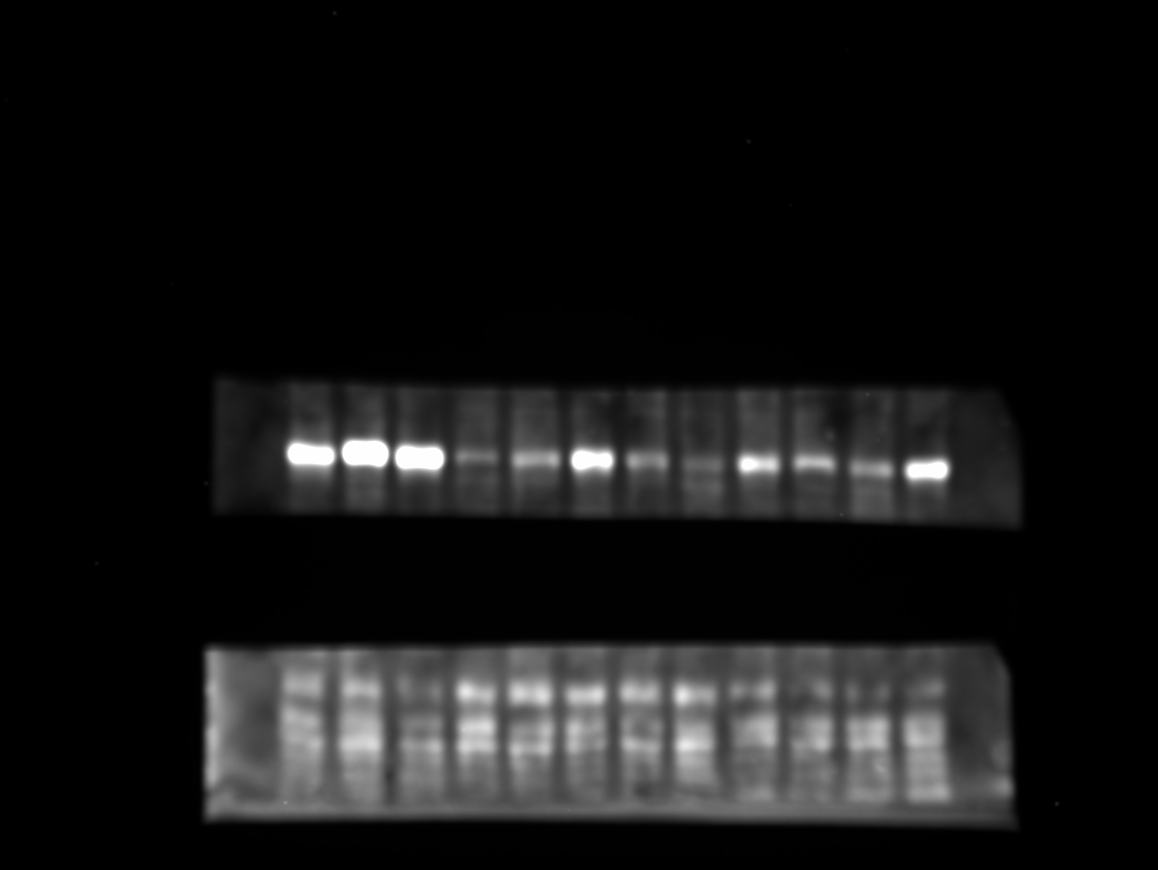

Supplement: Figure 1—figure supplement 2—source data 1. [file elife-85103-fig1-figsupp2-data1.zip › Figure 1 - figure supplement 2 - source data/source data Figure 1 - figure supplement 2D-F/BAT_GLUT4.tif]

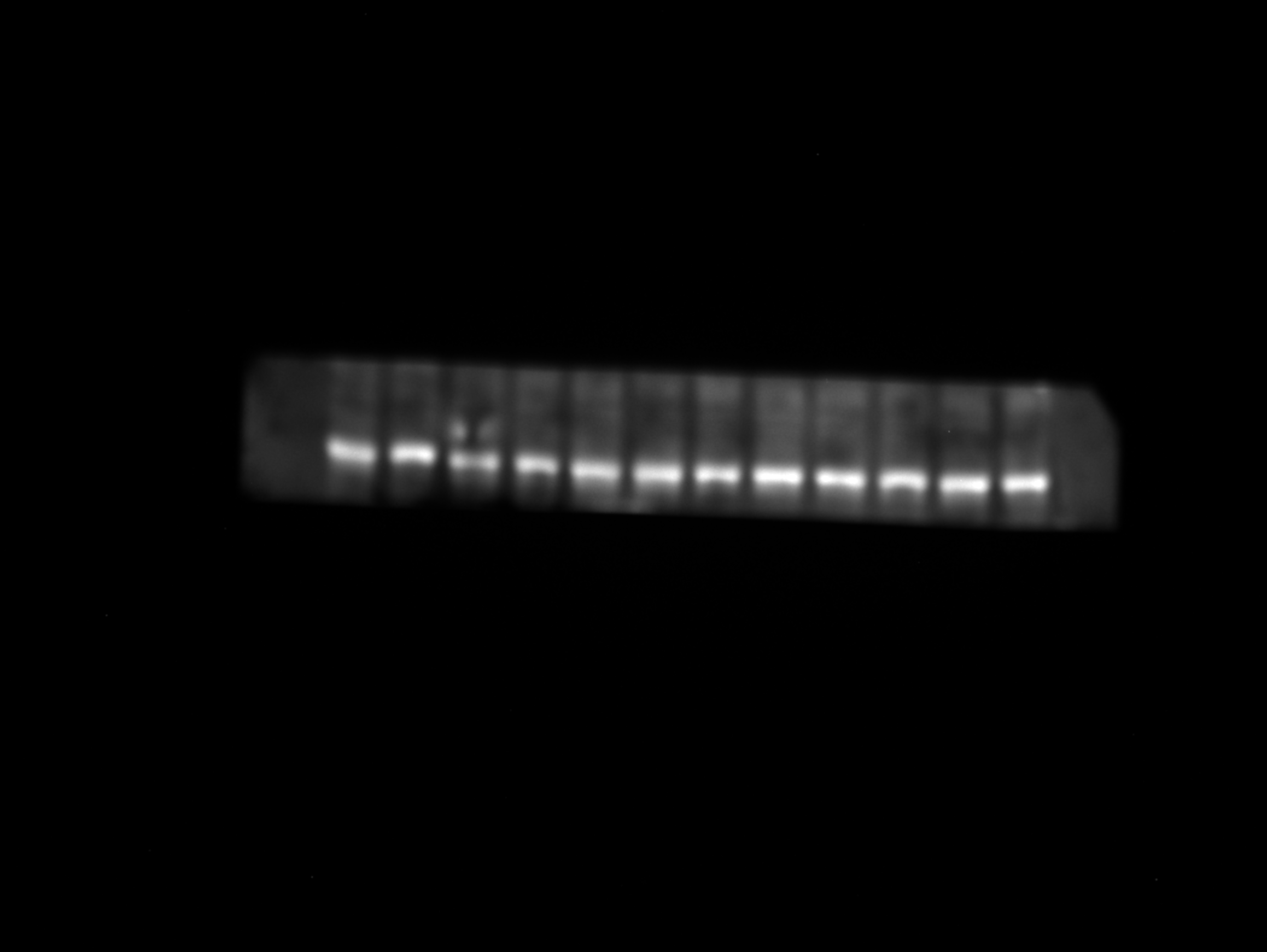

Supplement: Figure 1—figure supplement 2—source data 1. [file elife-85103-fig1-figsupp2-data1.zip › Figure 1 - figure supplement 2 - source data/source data Figure 1 - figure supplement 2D-F/BAT_AKT.tif]

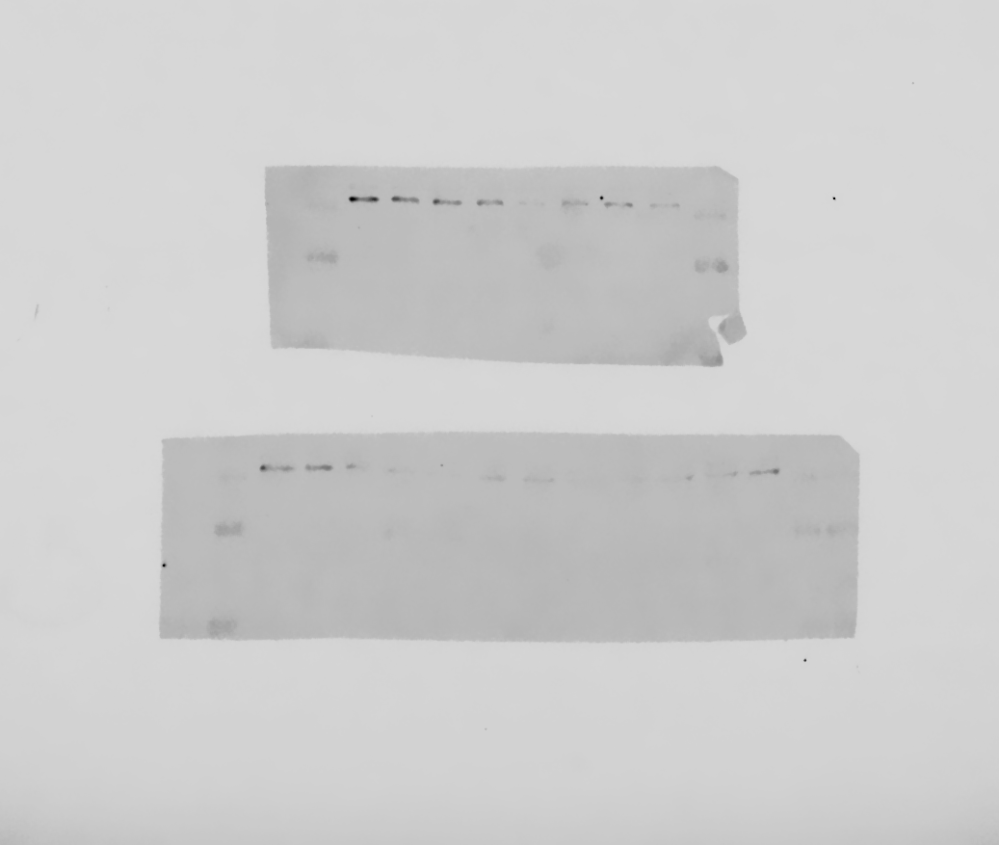

Supplement: Figure 1—figure supplement 2—source data 1. [file elife-85103-fig1-figsupp2-data1.zip › Figure 1 - figure supplement 2 - source data/source data Figure 1 - figure supplement 2D-F/vWAT_HK2.png]

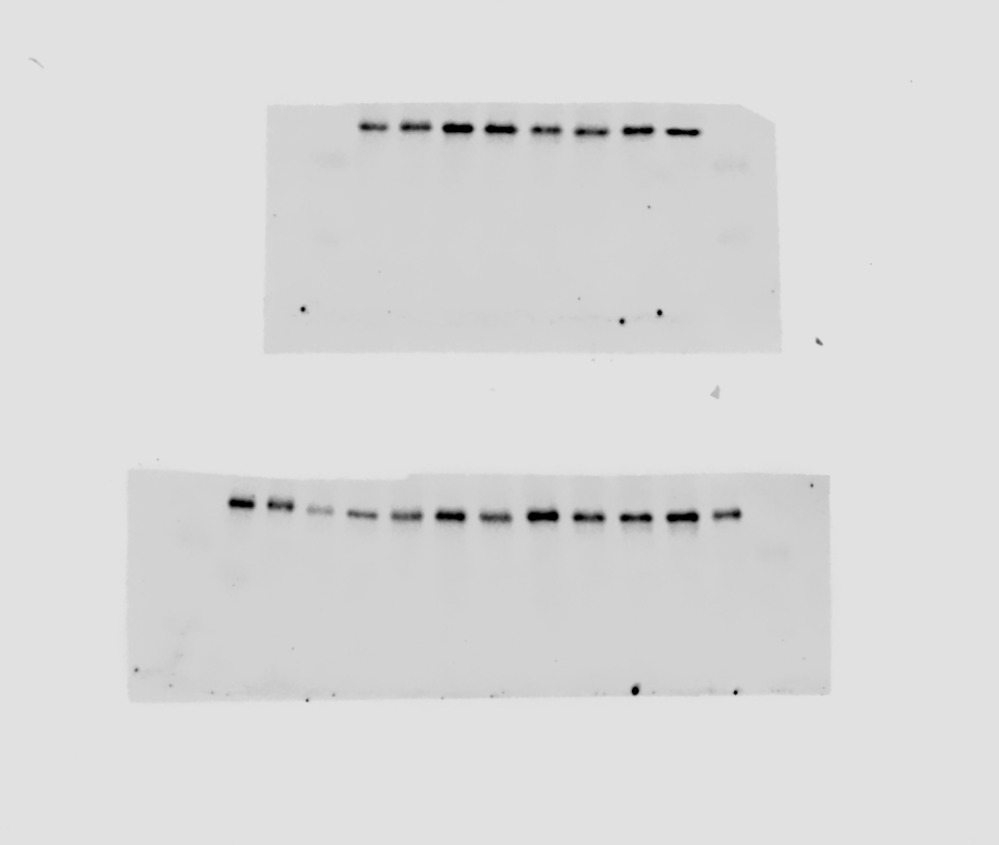

Supplement: Figure 1—figure supplement 2—source data 1. [file elife-85103-fig1-figsupp2-data1.zip › Figure 1 - figure supplement 2 - source data/source data Figure 1 - figure supplement 2D-F/sWAT_AKT-pS473.tif]

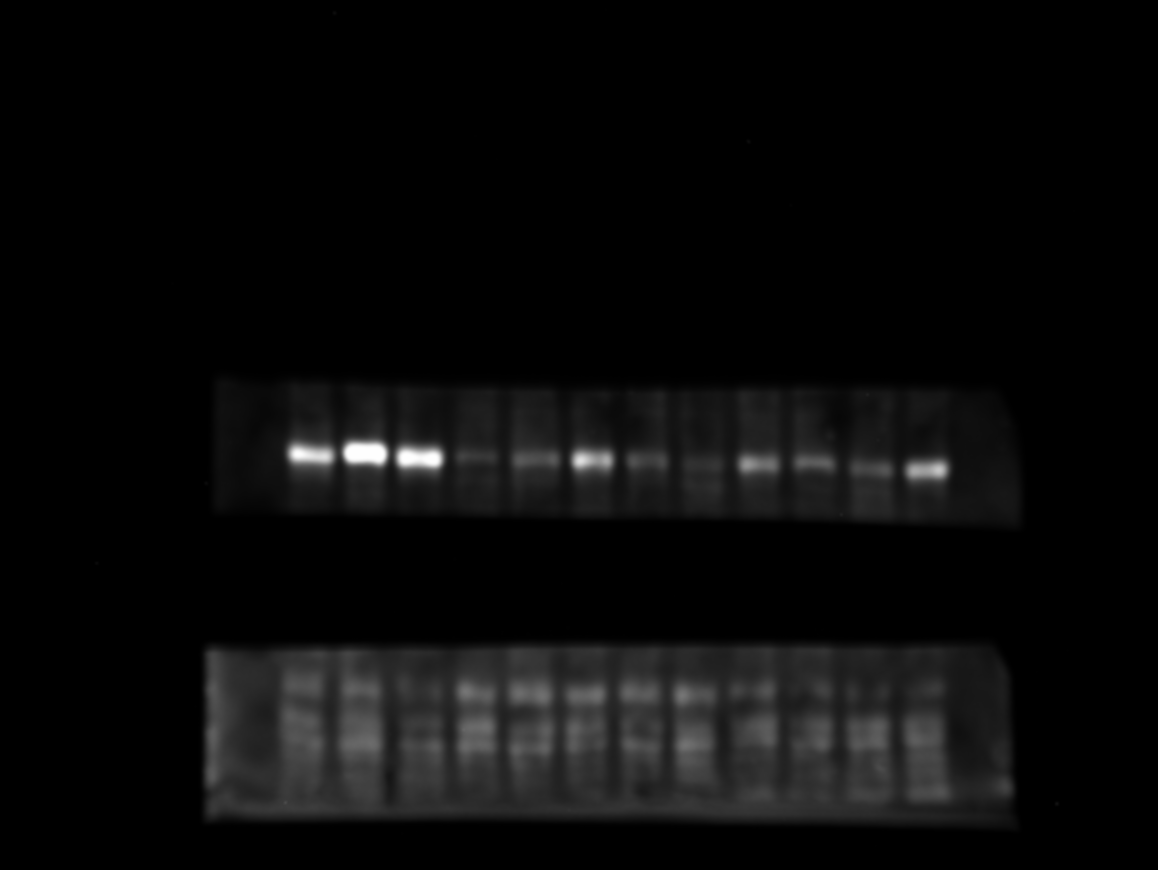

Supplement: Figure 1—figure supplement 2—source data 1. [file elife-85103-fig1-figsupp2-data1.zip › Figure 1 - figure supplement 2 - source data/source data Figure 1 - figure supplement 2D-F/BAT_AKTpS473.tif]

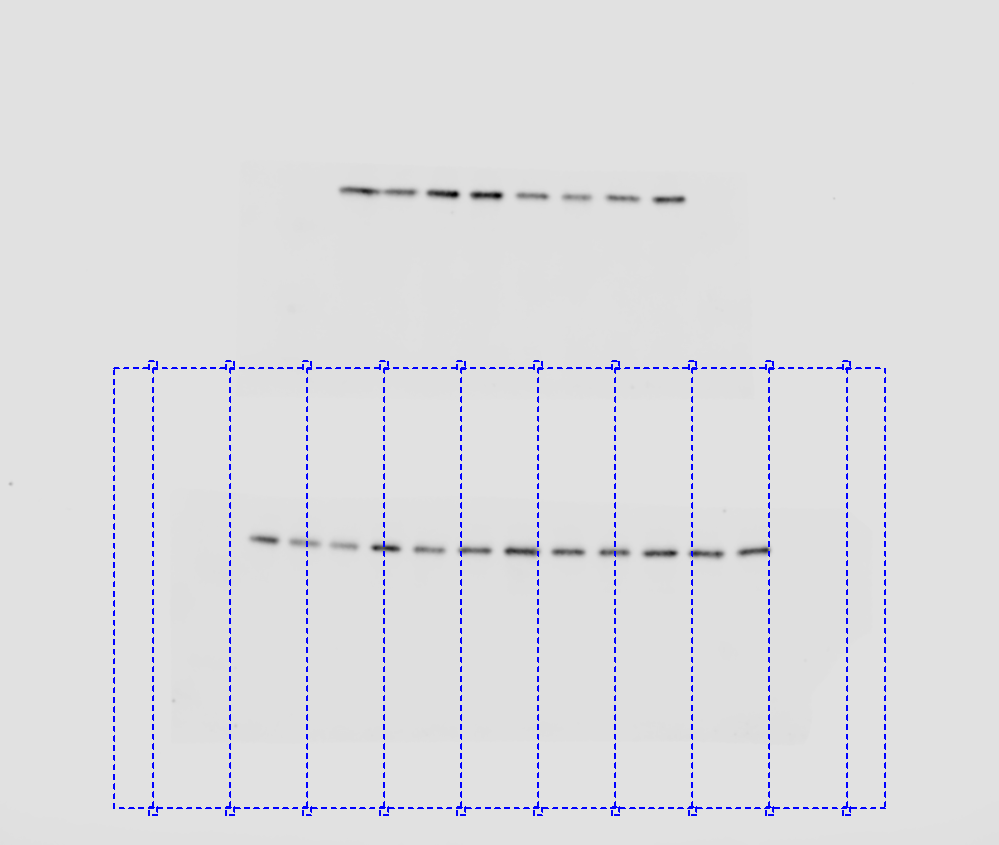

Supplement: Figure 1—figure supplement 2—source data 1. [file elife-85103-fig1-figsupp2-data1.zip › Figure 1 - figure supplement 2 - source data/source data Figure 1 - figure supplement 2D-F/vWAT_AKT-pS473.tif]

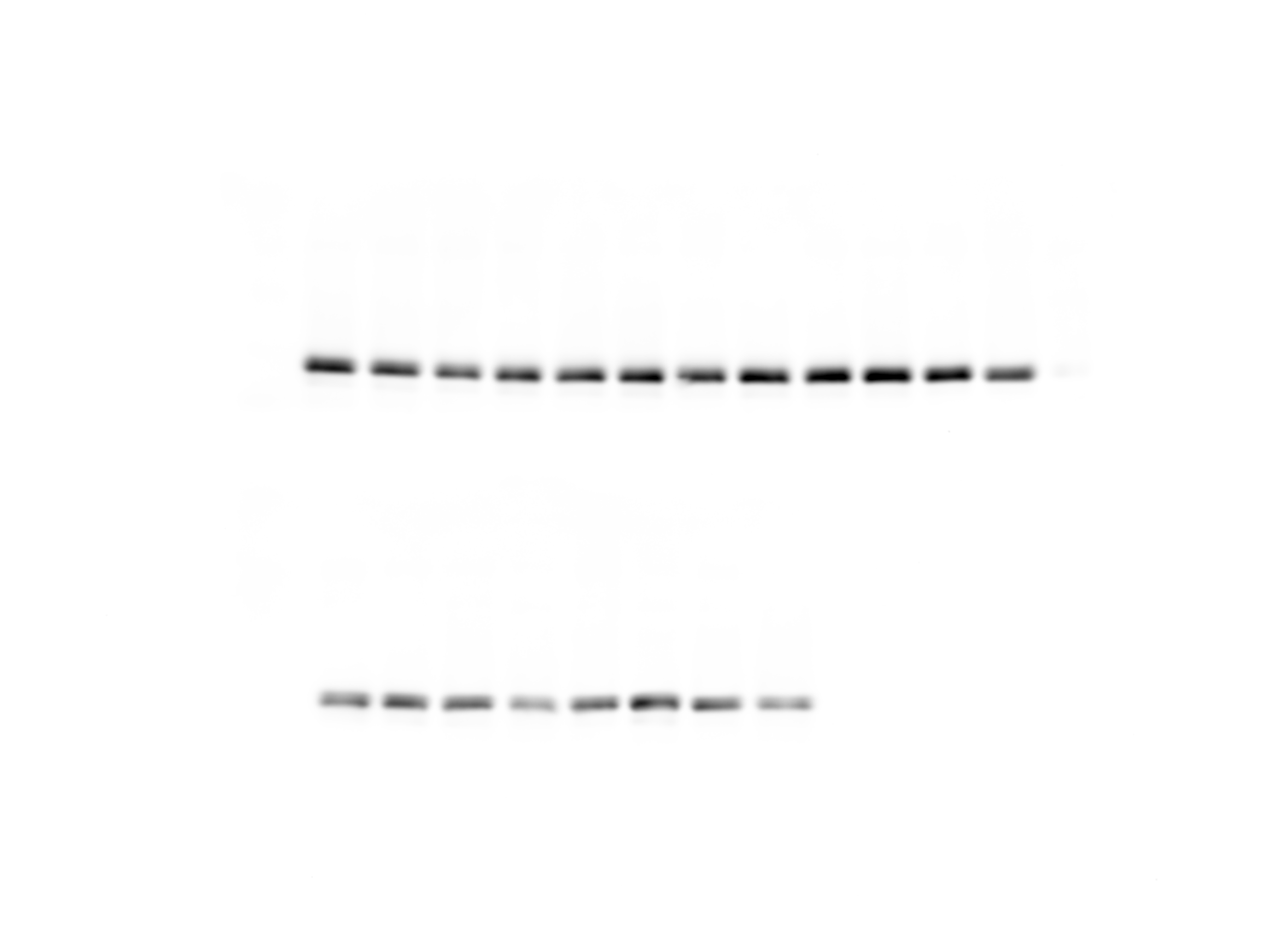

Supplement: Figure 1—figure supplement 2—source data 1. [file elife-85103-fig1-figsupp2-data1.zip › Figure 1 - figure supplement 2 - source data/source data Figure 1 - figure supplement 2D-F/vWAT_CALX.tif]

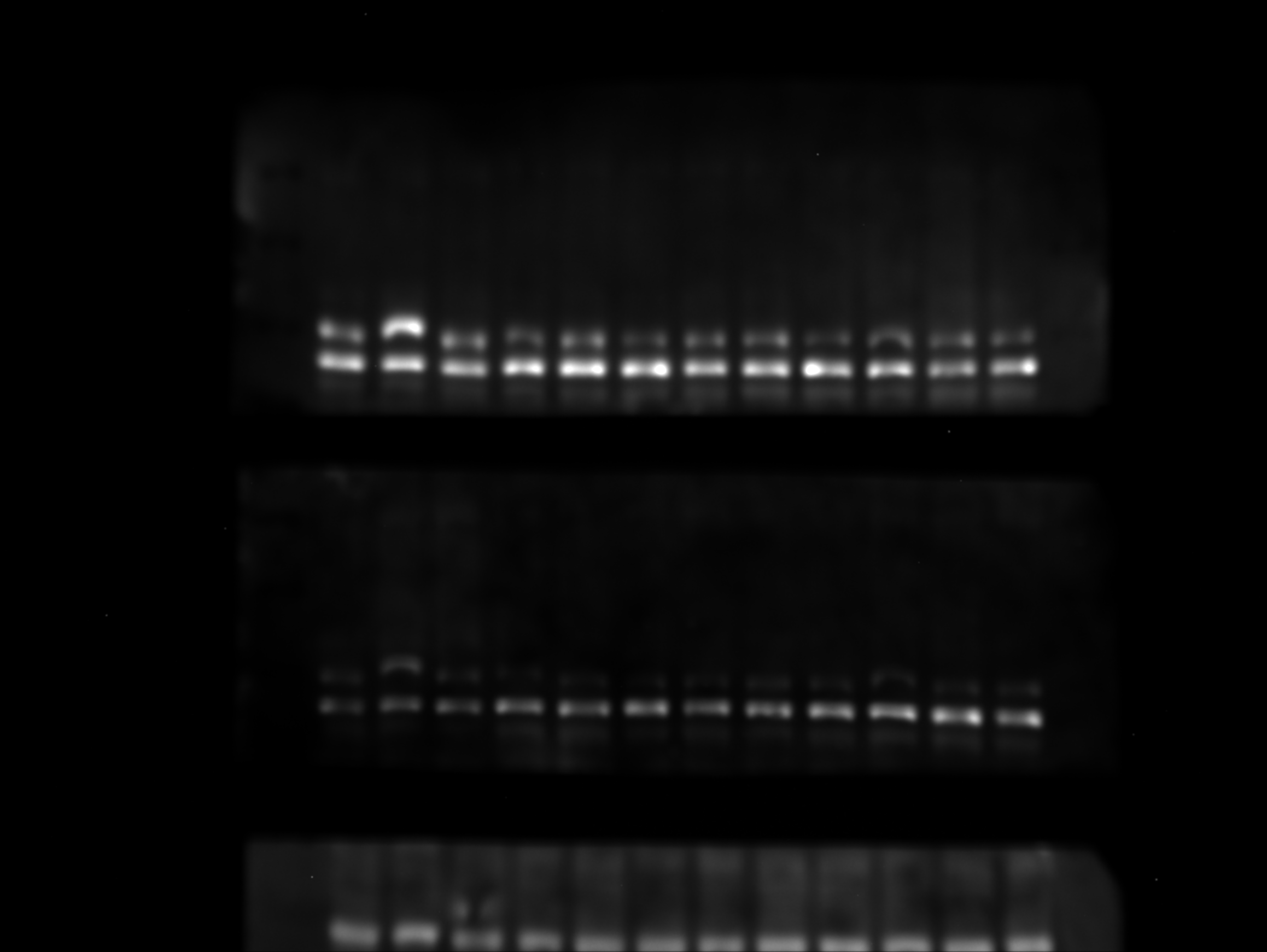

Supplement: Figure 1—figure supplement 2—source data 1. [file elife-85103-fig1-figsupp2-data1.zip › Figure 1 - figure supplement 2 - source data/source data Figure 1 - figure supplement 2D-F/BAT_CALX.tif]

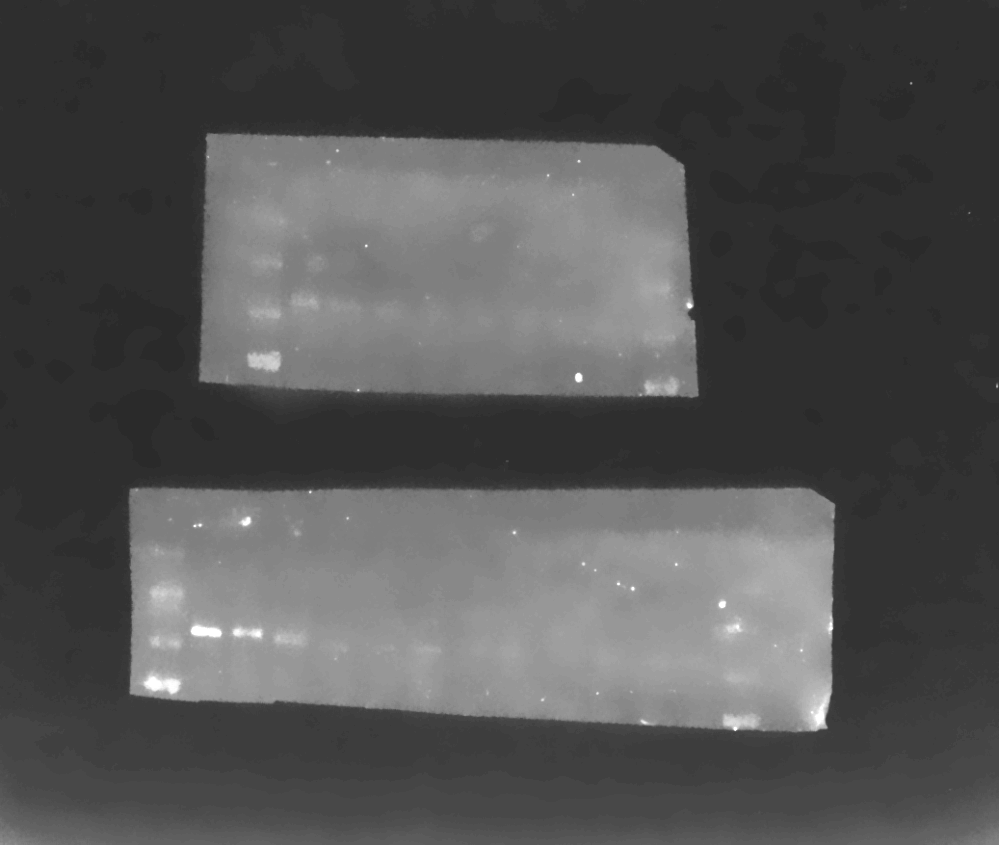

Supplement: Figure 1—figure supplement 2—source data 1. [file elife-85103-fig1-figsupp2-data1.zip › Figure 1 - figure supplement 2 - source data/source data Figure 1 - figure supplement 2D-F/sWAT_HK2.tif]

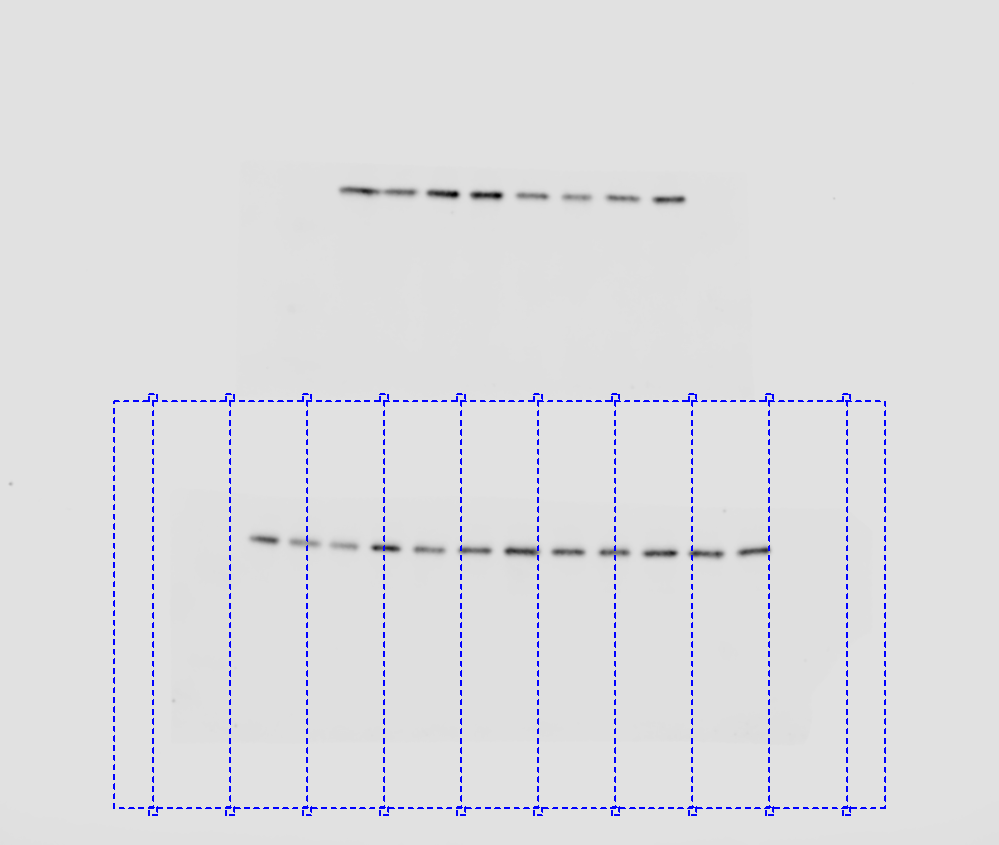

Supplement: Figure 1—figure supplement 2—source data 1. [file elife-85103-fig1-figsupp2-data1.zip › Figure 1 - figure supplement 2 - source data/source data Figure 1 - figure supplement 2D-F/vWAT_AKT.png]

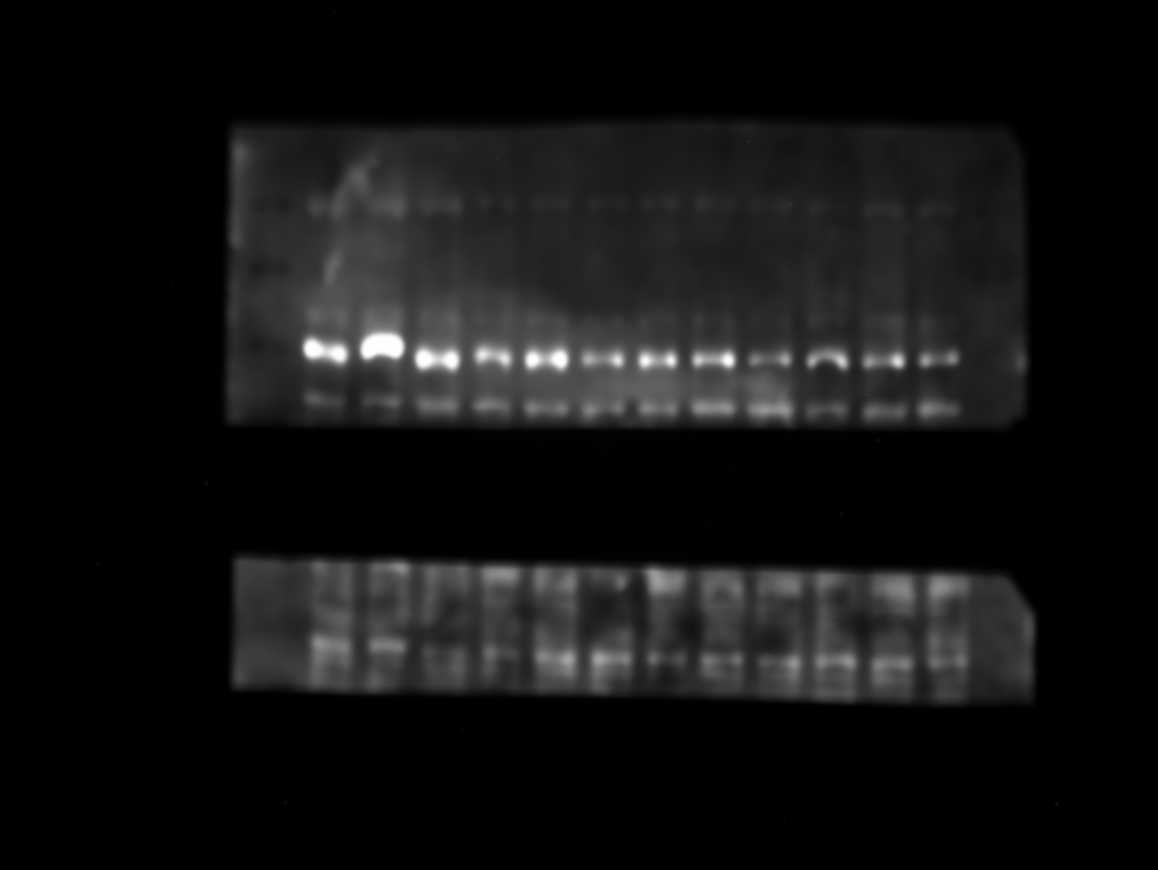

Supplement: Figure 1—figure supplement 2—source data 1. [file elife-85103-fig1-figsupp2-data1.zip › Figure 1 - figure supplement 2 - source data/source data Figure 1 - figure supplement 2D-F/BAT_HK2.tif]

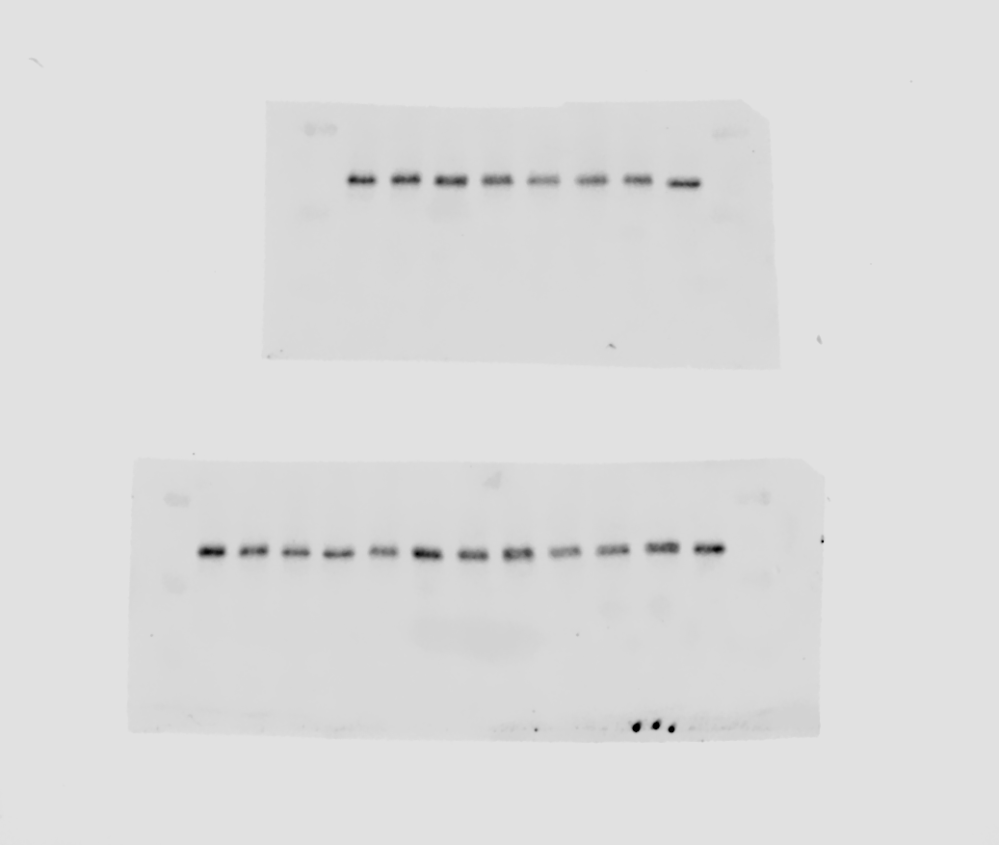

Supplement: Figure 1—figure supplement 2—source data 1. [file elife-85103-fig1-figsupp2-data1.zip › Figure 1 - figure supplement 2 - source data/source data Figure 1 - figure supplement 2D-F/sWAT_AKT.tif]

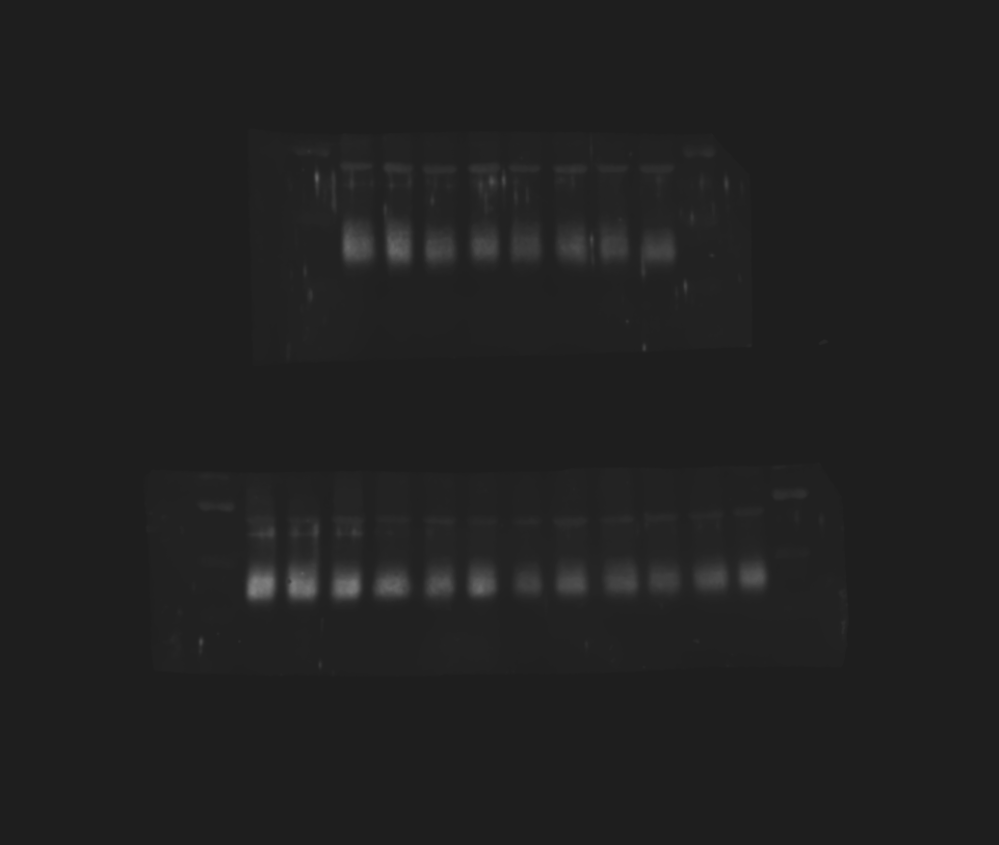

Supplement: Figure 1—figure supplement 2—source data 1. [file elife-85103-fig1-figsupp2-data1.zip › Figure 1 - figure supplement 2 - source data/source data Figure 1 - figure supplement 2D-F/sWAT_GLUT4.tif]

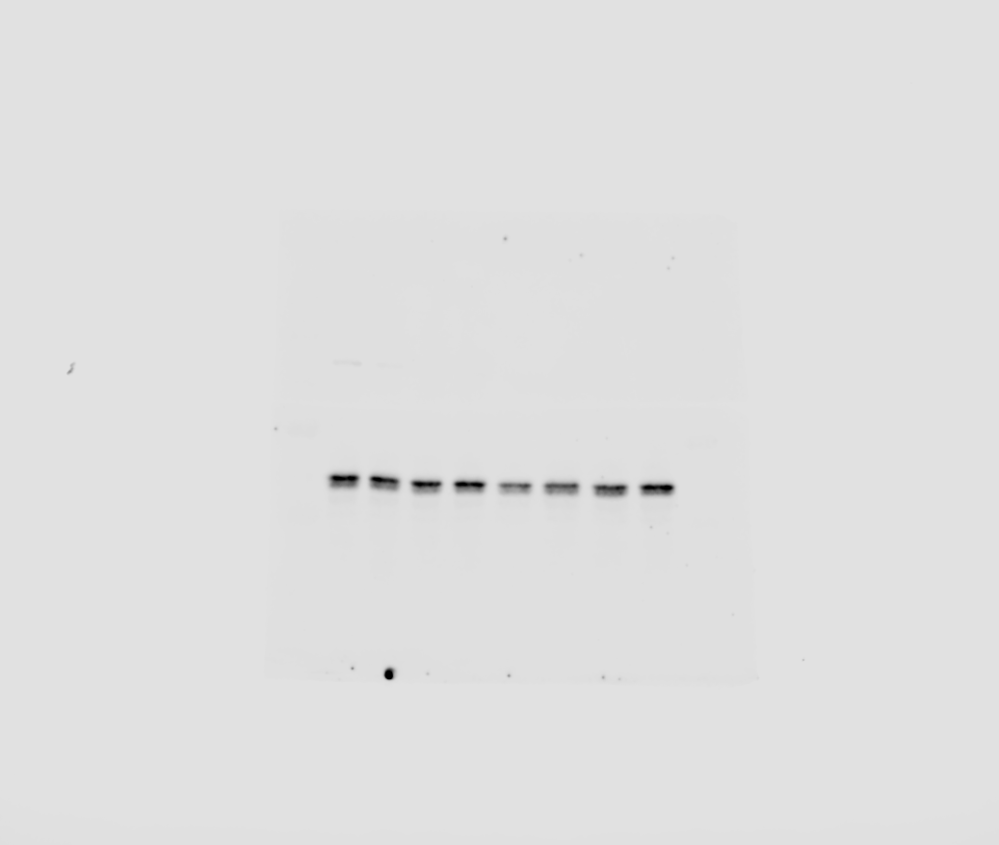

Supplement: Figure 1—figure supplement 2—source data 1. [file elife-85103-fig1-figsupp2-data1.zip › Figure 1 - figure supplement 2 - source data/source data Figure 1 - figure supplement 2D-F/vWAT_AKT.tif]

Figure 2G

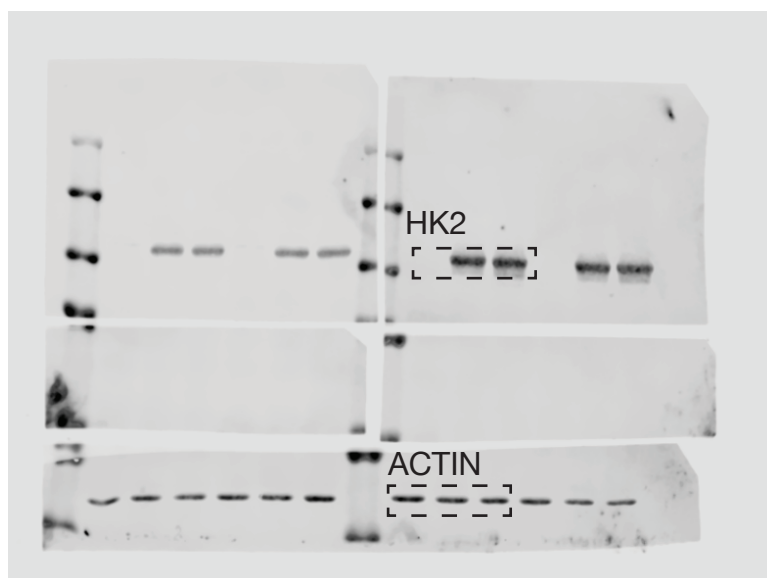

Supplement: Figure 2—source data 1. [file elife-85103-fig2-data1.zip › Figure 2 - source data/Figure2G - source data.pdf]

Figure 2F

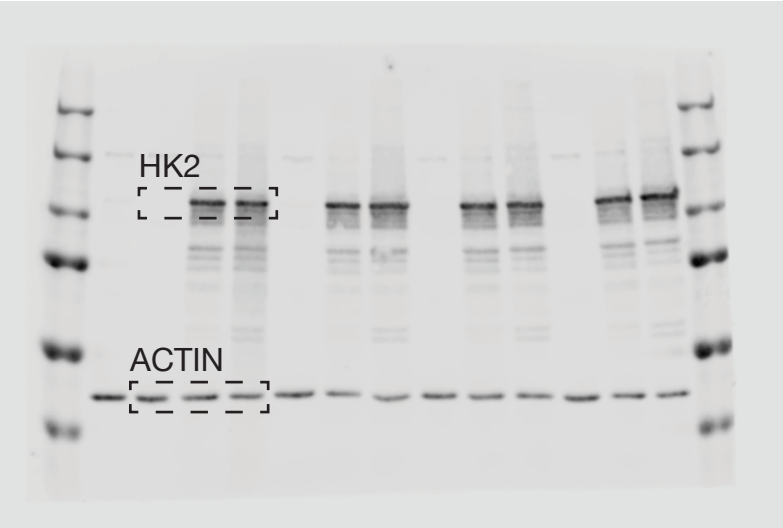

Supplement: Figure 2—source data 1. [file elife-85103-fig2-data1.zip › Figure 2 - source data/Figure2F - source data.pdf]

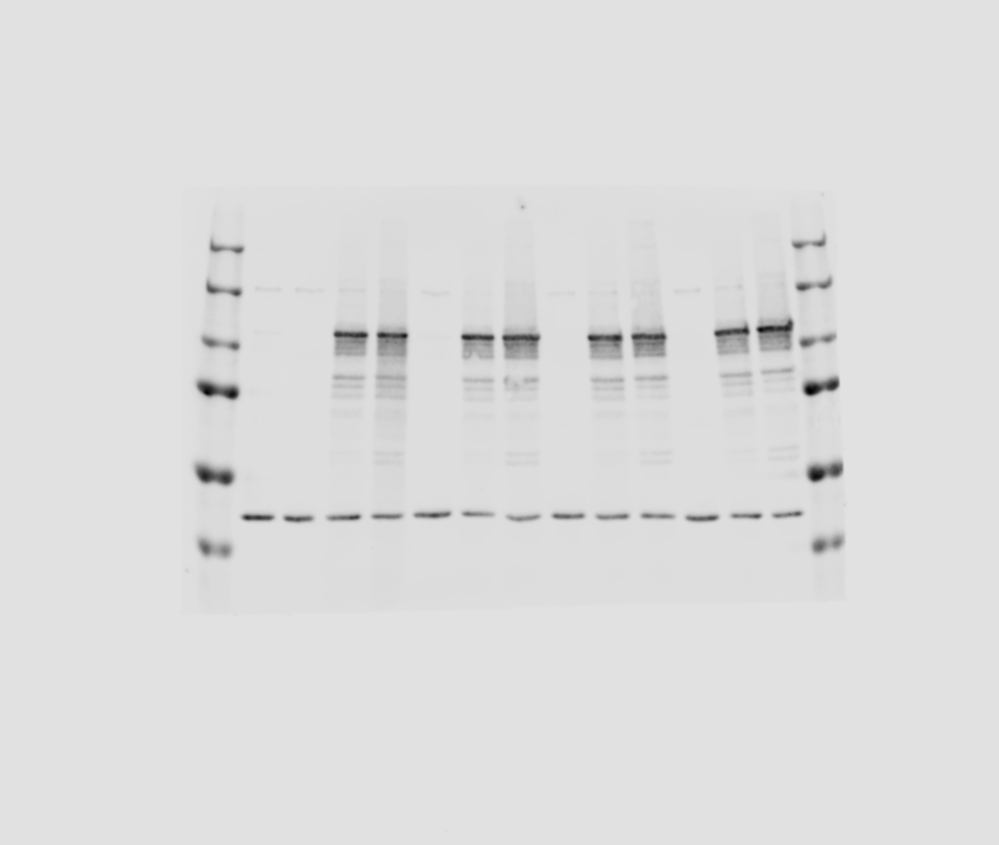

Supplement: Figure 2—source data 1. [file elife-85103-fig2-data1.zip › Figure 2 - source data/source data Figure2F/HK2_ACTIN.tif]

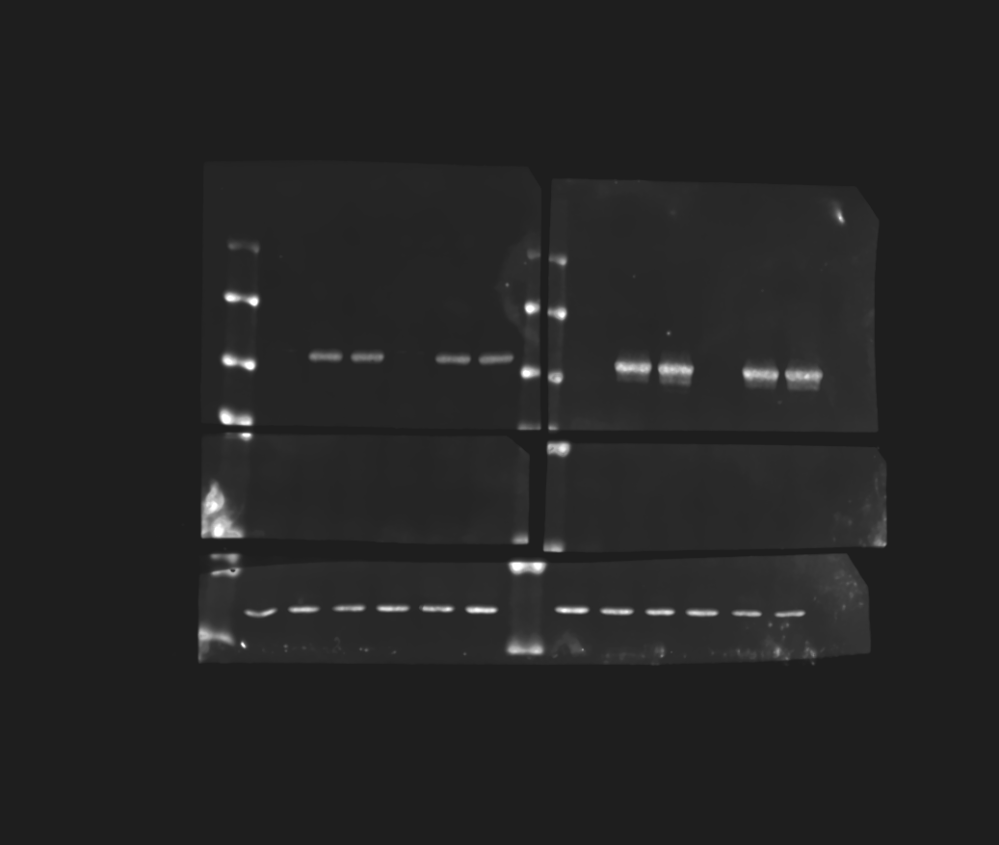

Supplement: Figure 2—source data 1. [file elife-85103-fig2-data1.zip › Figure 2 - source data/source data Figure2G/HK2_ACTIN.tif]

Figure 3A

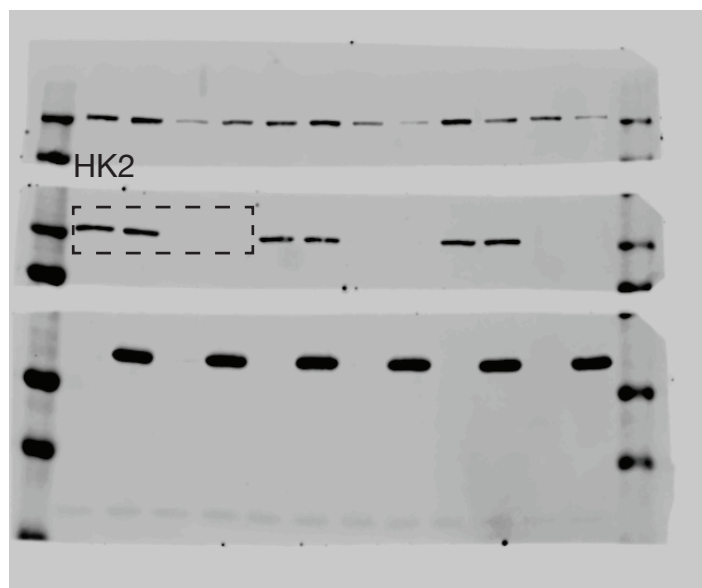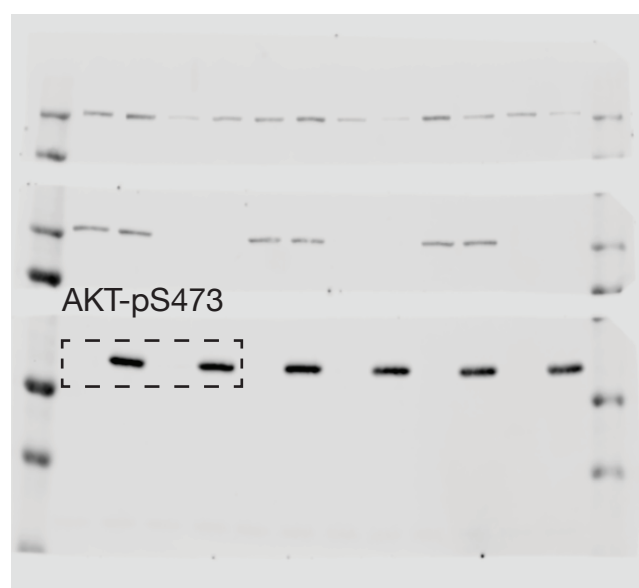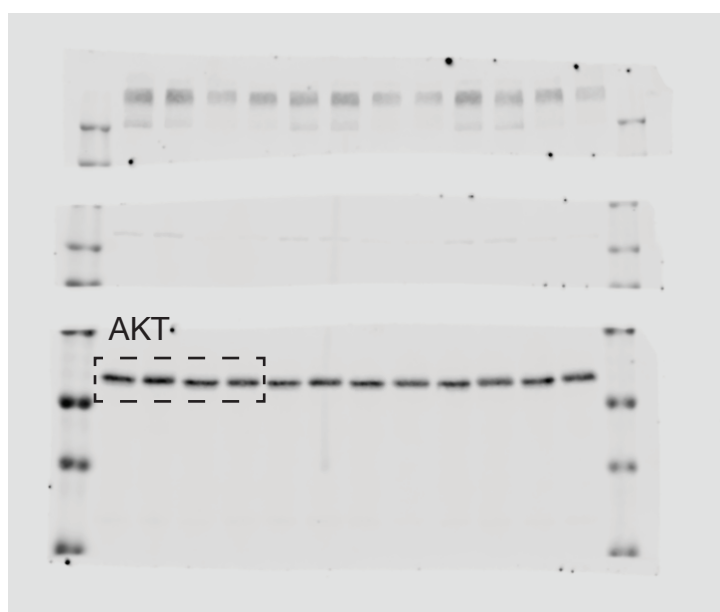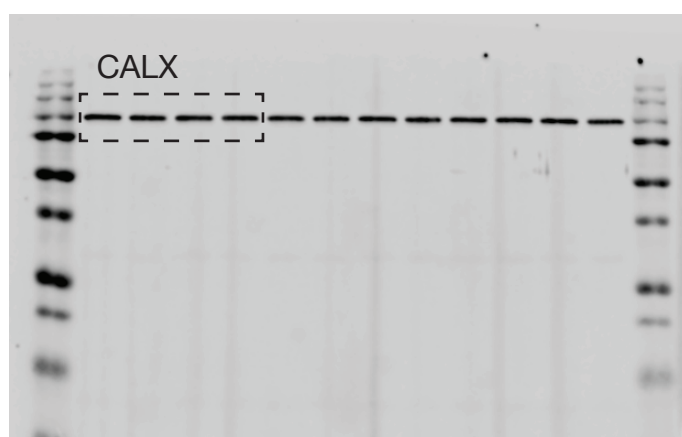

Supplement: Figure 3—source data 1. [file elife-85103-fig3-data1.zip › Figure 3 - source data/Figure 3A - source data.pdf]

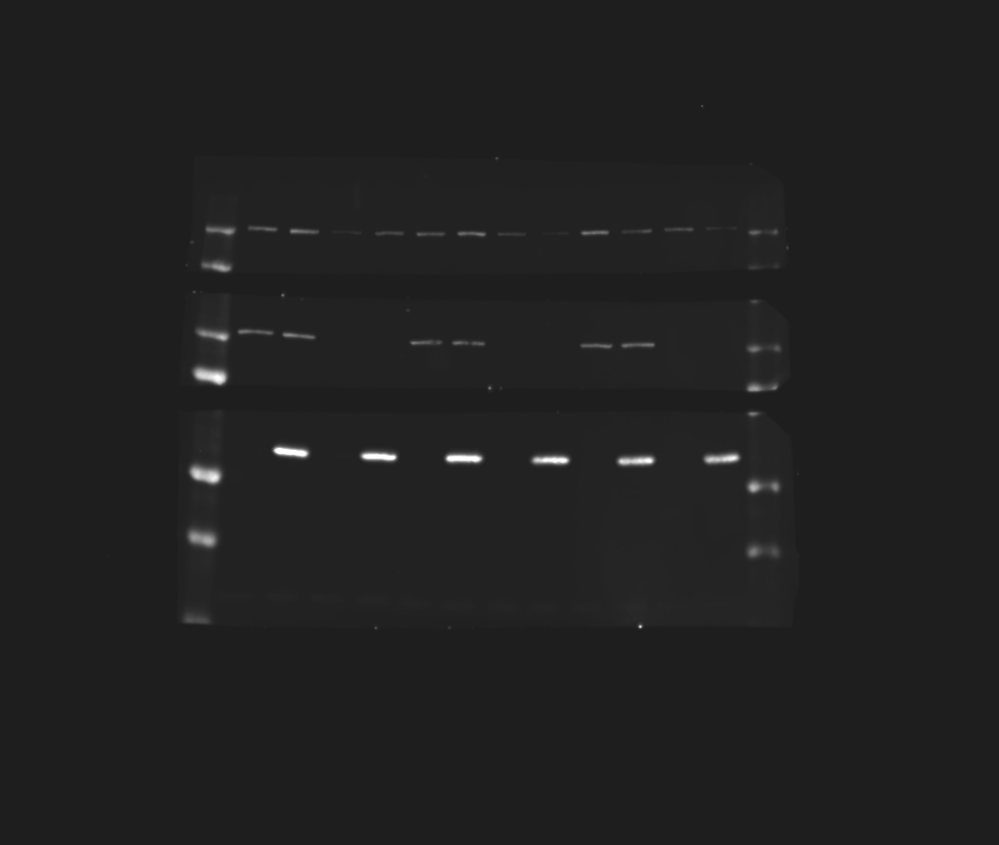

Supplement: Figure 3—source data 1. [file elife-85103-fig3-data1.zip › Figure 3 - source data/source data Figure3A/HK2_AKT-pS473.tif]

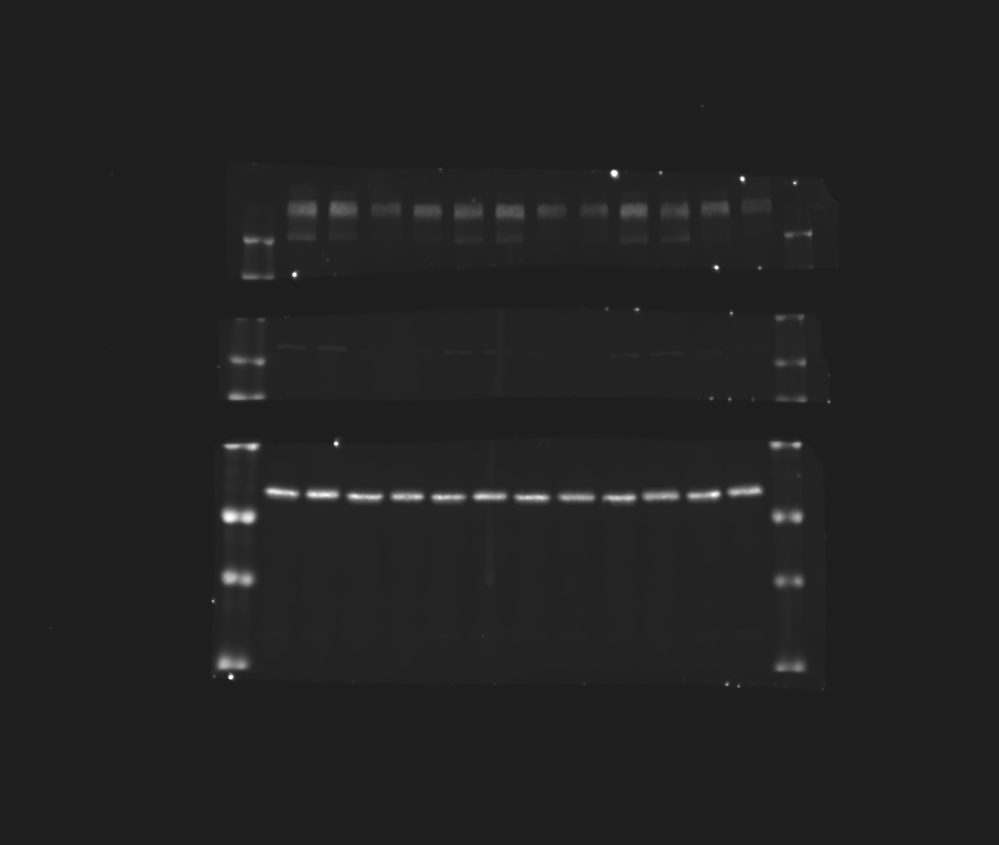

Supplement: Figure 3—source data 1. [file elife-85103-fig3-data1.zip › Figure 3 - source data/source data Figure3A/AKT.tif]

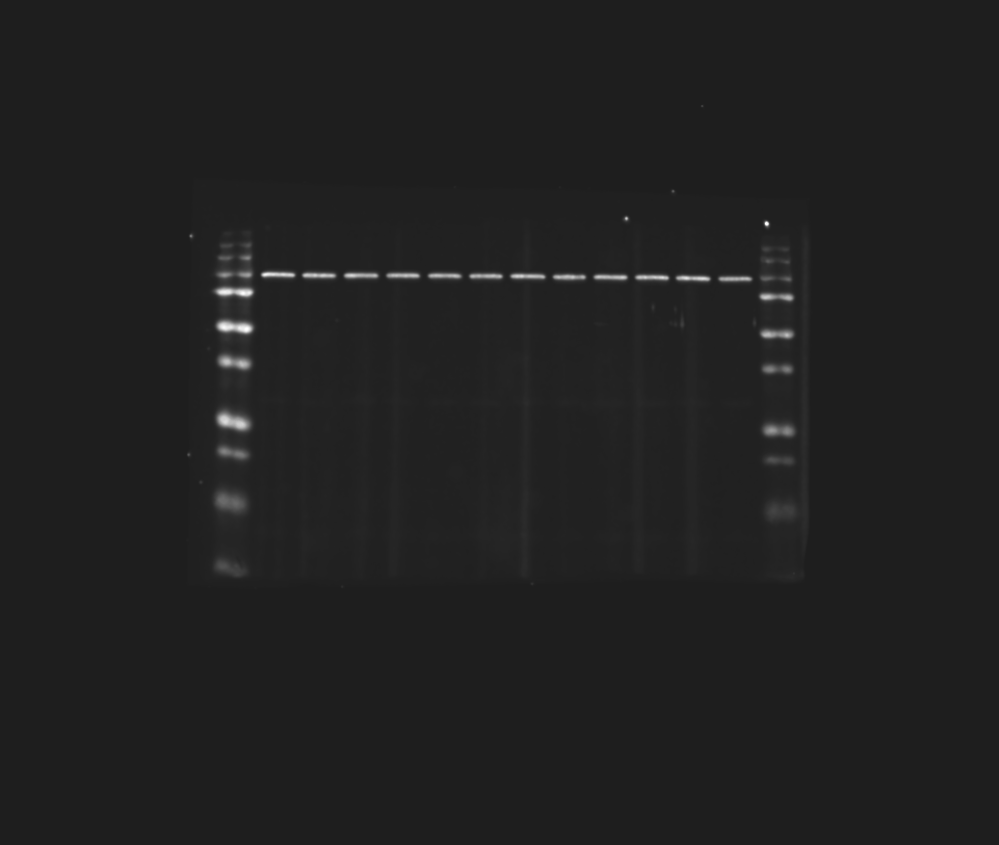

Supplement: Figure 3—source data 1. [file elife-85103-fig3-data1.zip › Figure 3 - source data/source data Figure3A/CALX.tif]

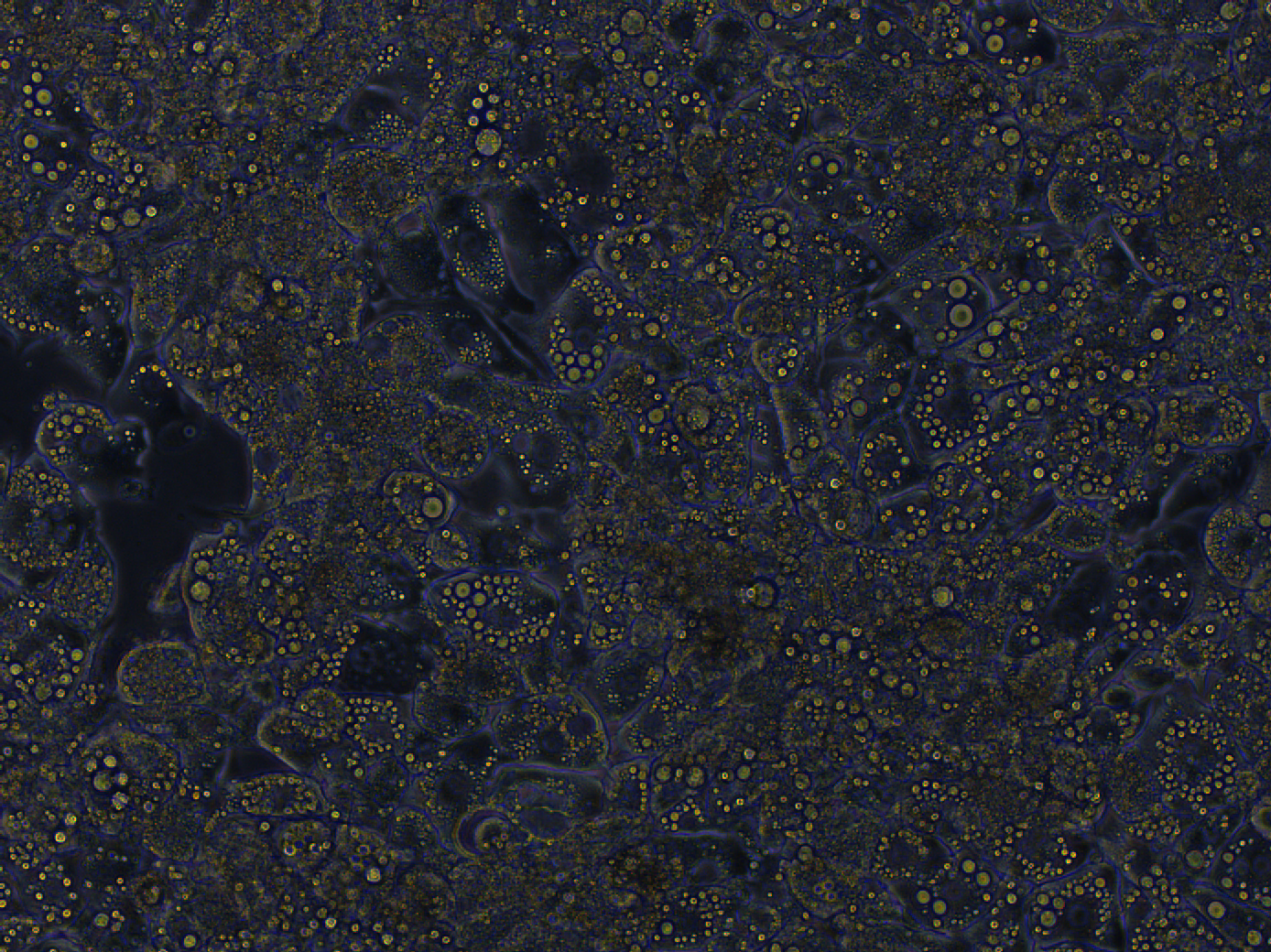

Supplement: Figure 3—figure supplement 1—source data 1. [file elife-85103-fig3-figsupp1-data1.zip › Figure 3 - figure supplement 1 - source data/source data Figure 3 - figure supplement 1A/control.tif]

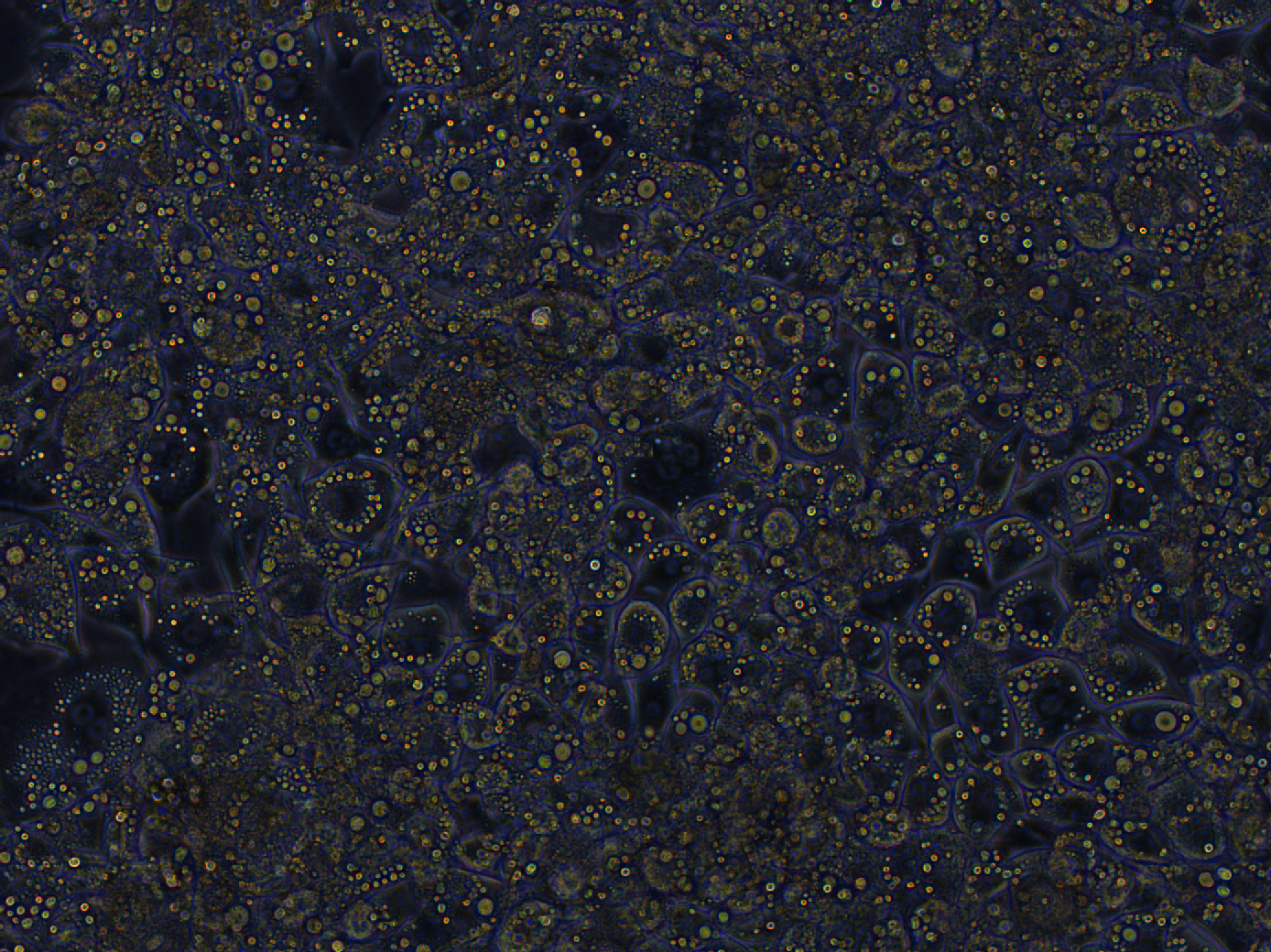

Supplement: Figure 3—figure supplement 1—source data 1. [file elife-85103-fig3-figsupp1-data1.zip › Figure 3 - figure supplement 1 - source data/source data Figure 3 - figure supplement 1A/shHK2.tif]

Figure 4A

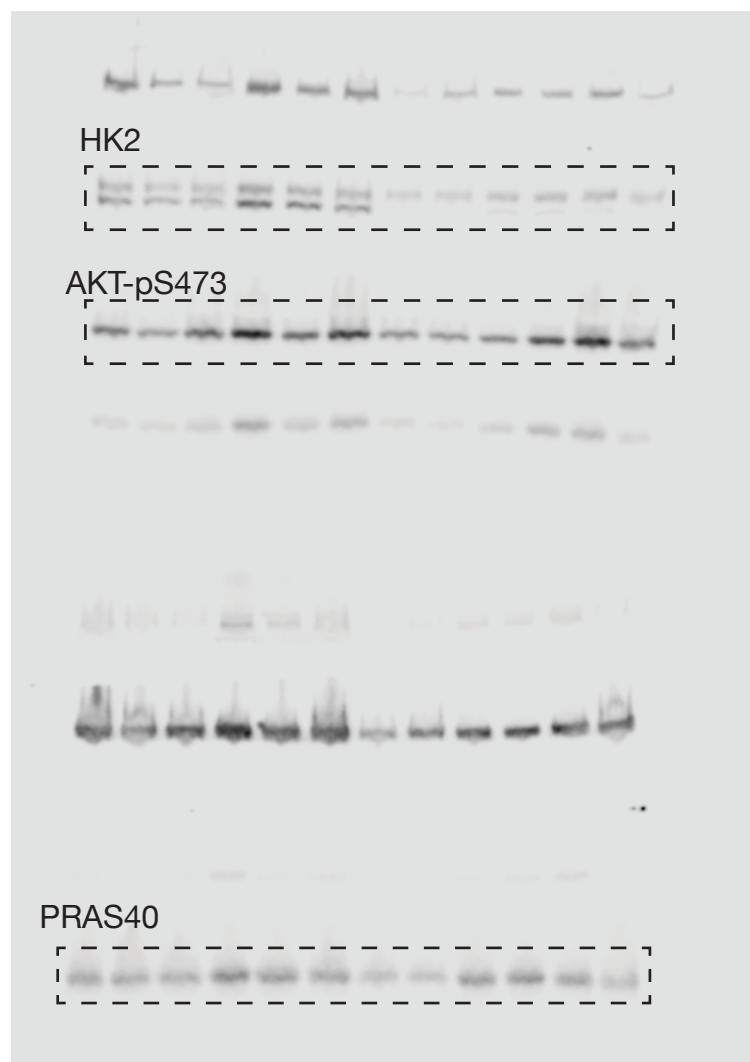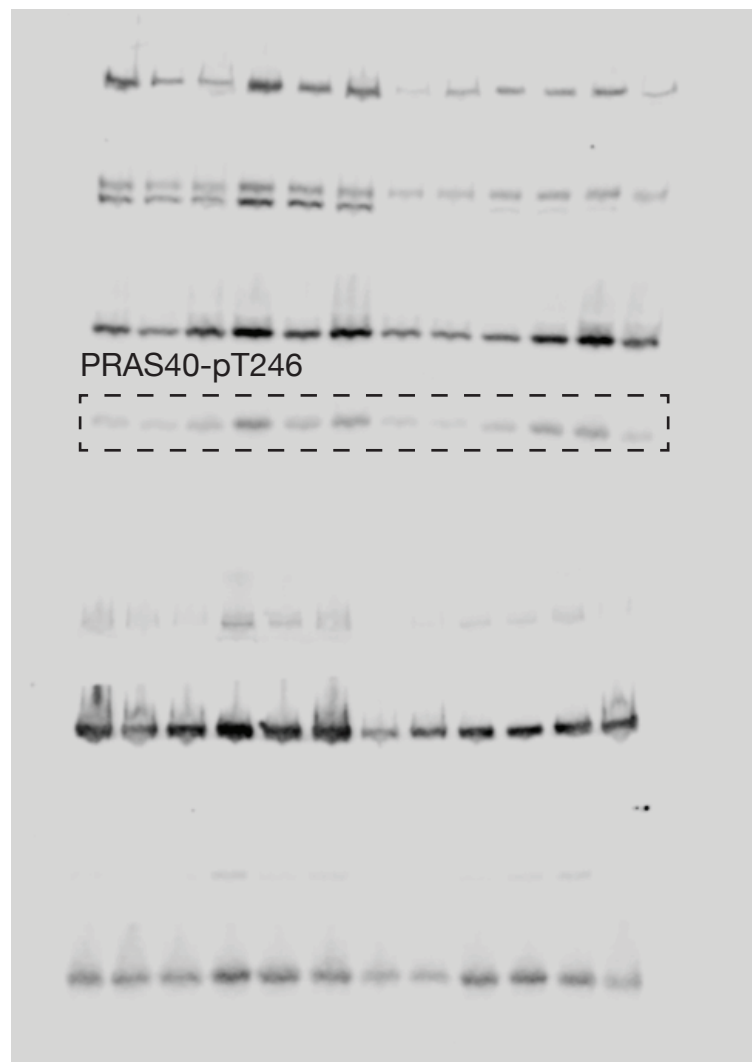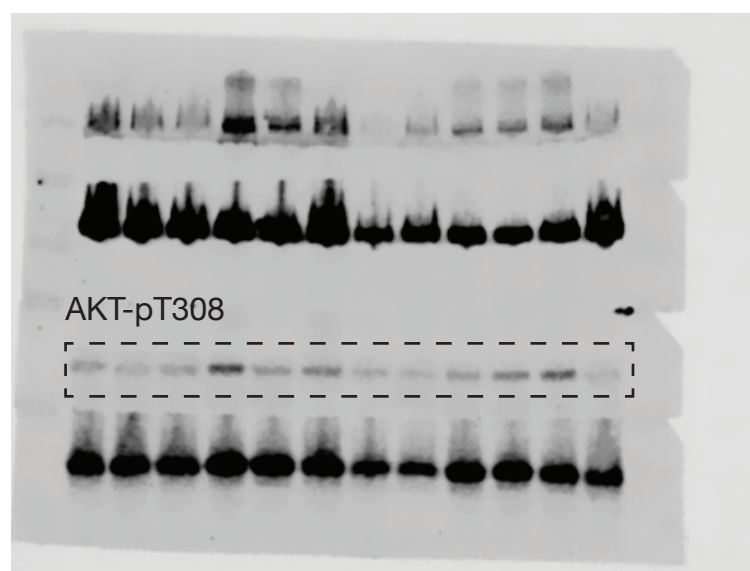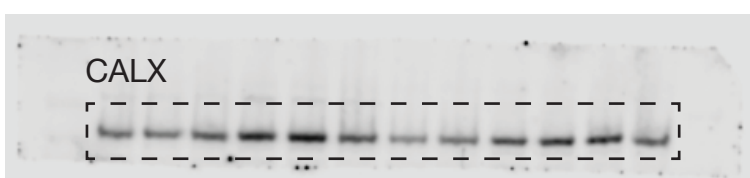

Supplement: Figure 4—source data 1. [file elife-85103-fig4-data1.zip › Figure 4- source data/Figure 4A - source data.pdf]

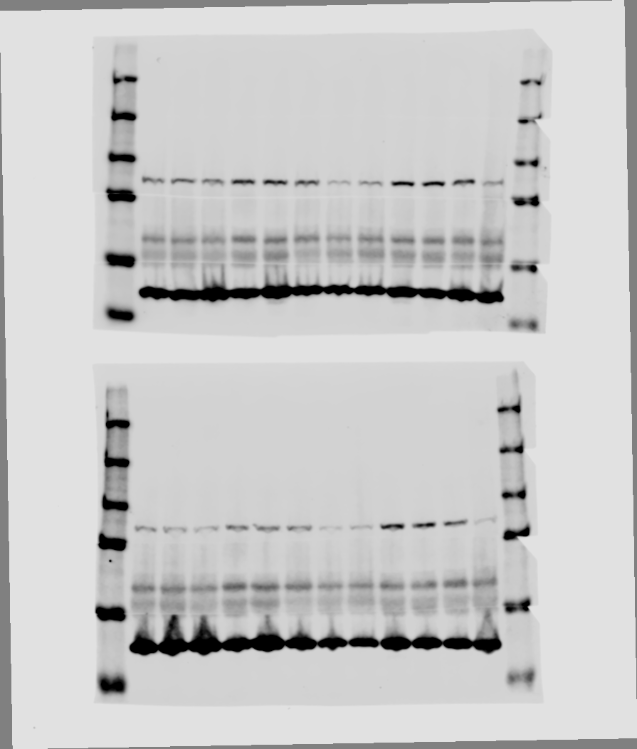

Supplement: Figure 4—source data 1. [file elife-85103-fig4-data1.zip › Figure 4- source data/source data Figure4A/AKT.tif]

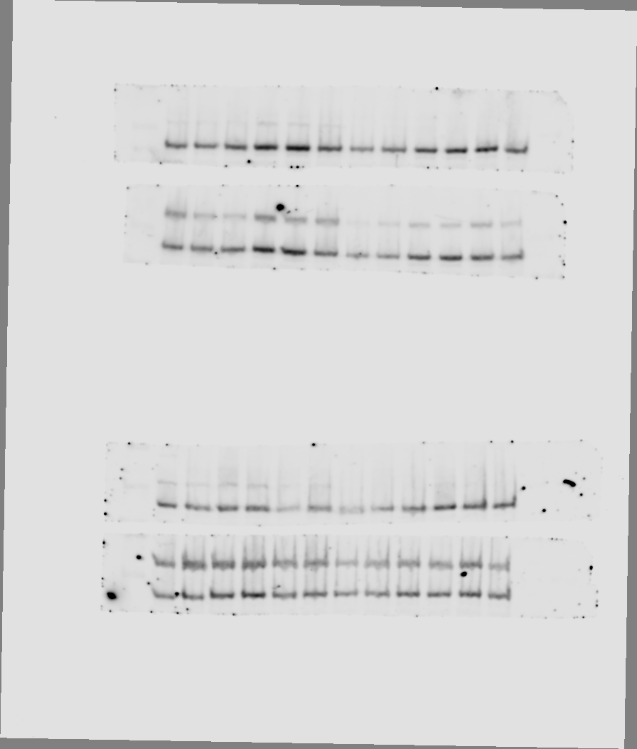

Supplement: Figure 4—source data 1. [file elife-85103-fig4-data1.zip › Figure 4- source data/source data Figure4A/CALX.tif]

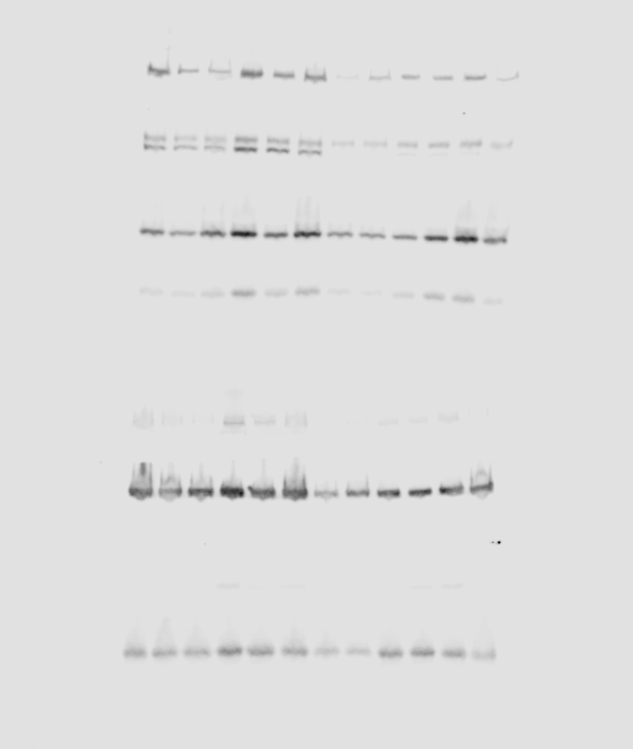

Supplement: Figure 4—source data 1. [file elife-85103-fig4-data1.zip › Figure 4- source data/source data Figure4A/HK2_AKT-pS473_PRAS40-pT246_PRAS40.tif]

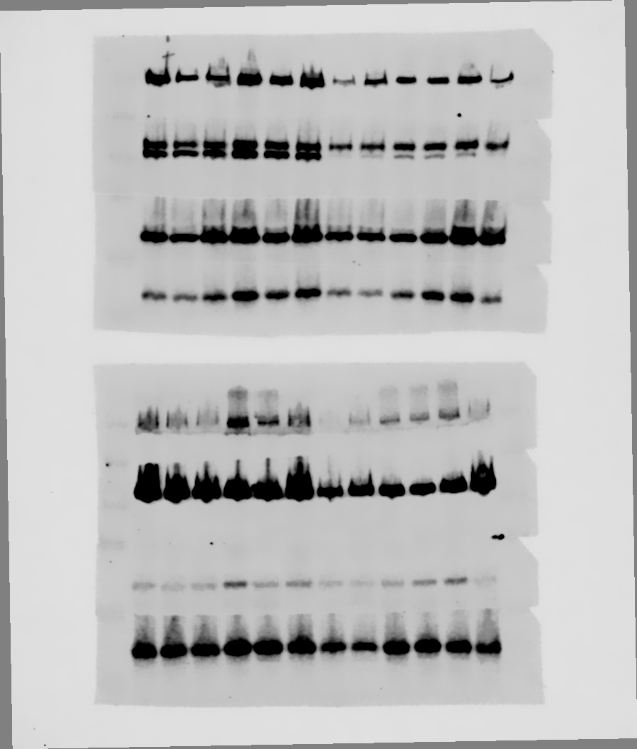

Supplement: Figure 4—source data 1. [file elife-85103-fig4-data1.zip › Figure 4- source data/source data Figure4A/AKT-pT308.tif]

Figure 4- figure supplement 1B

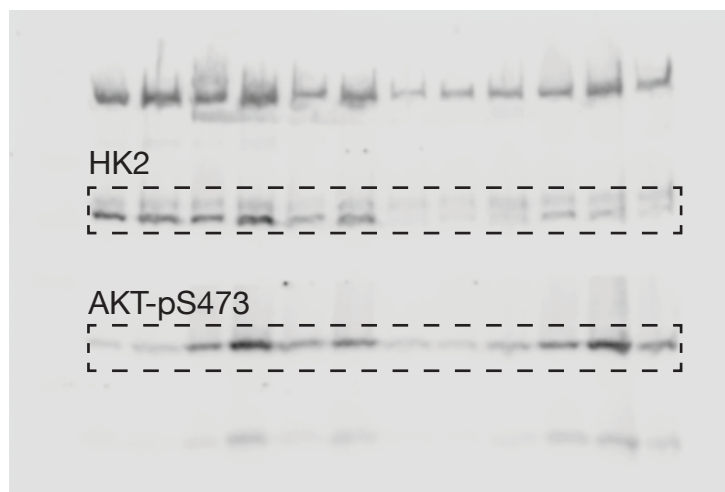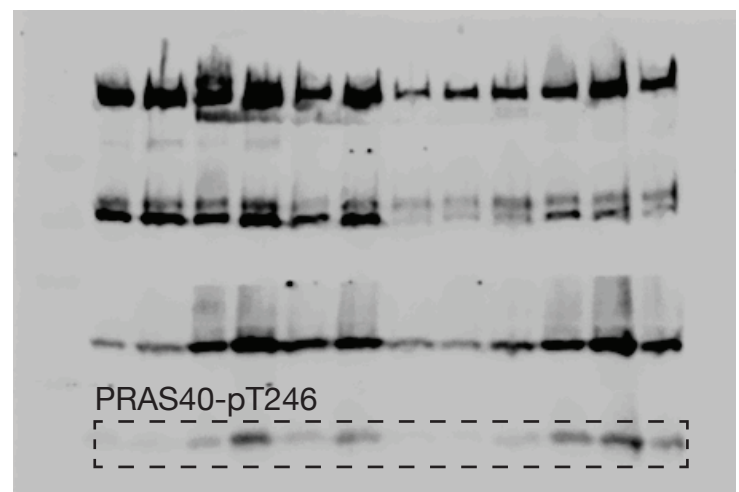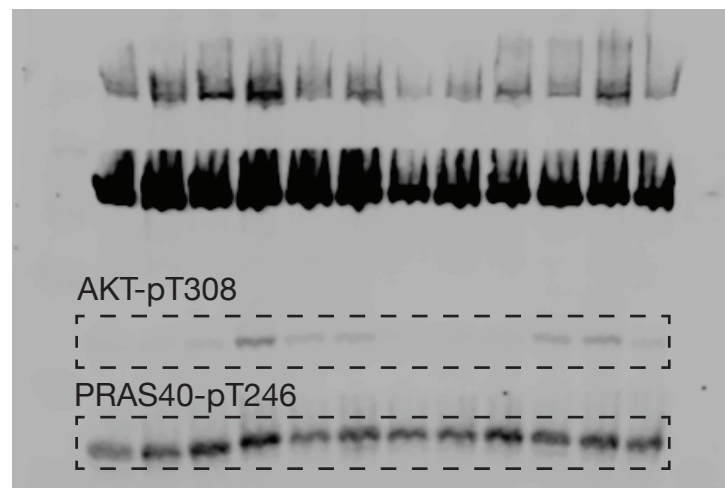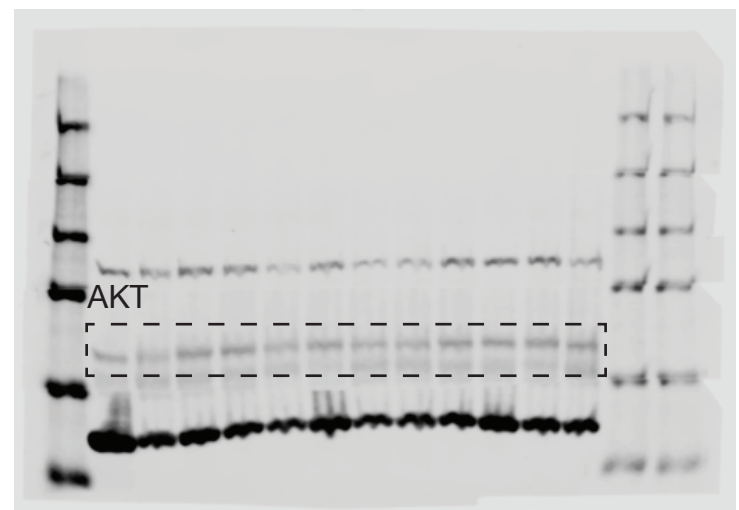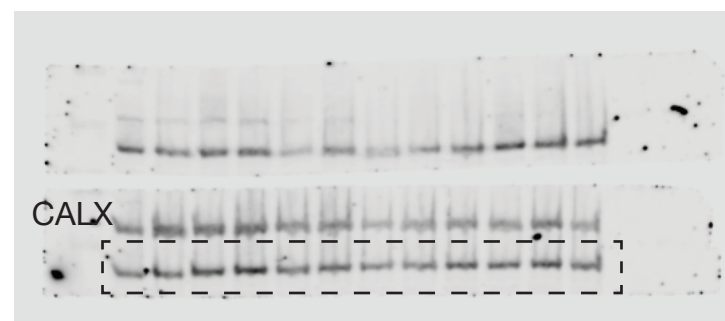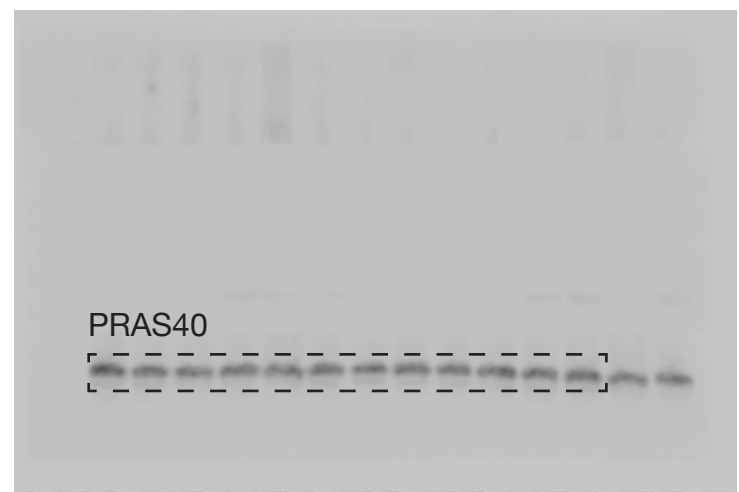

Figure 4- figure supplement 1C

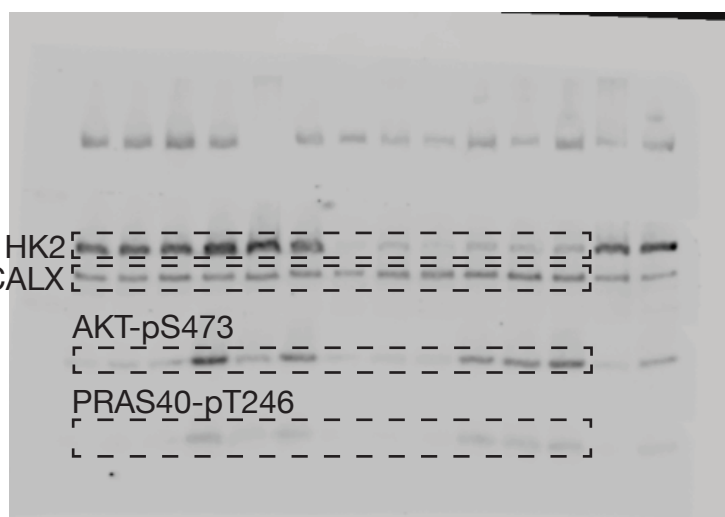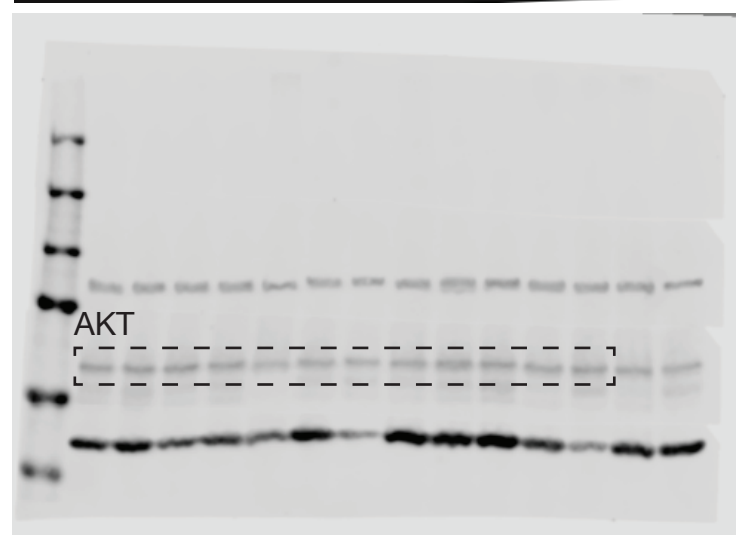

Supplement: Figure 4—figure supplement 1—source data 1. [file elife-85103-fig4-figsupp1-data1.zip › Figure 4 - figure supplement 1 - source data/Figure 4- figure supplement 1B-C - source data.pdf]

Figure 4- figure supplement 1D

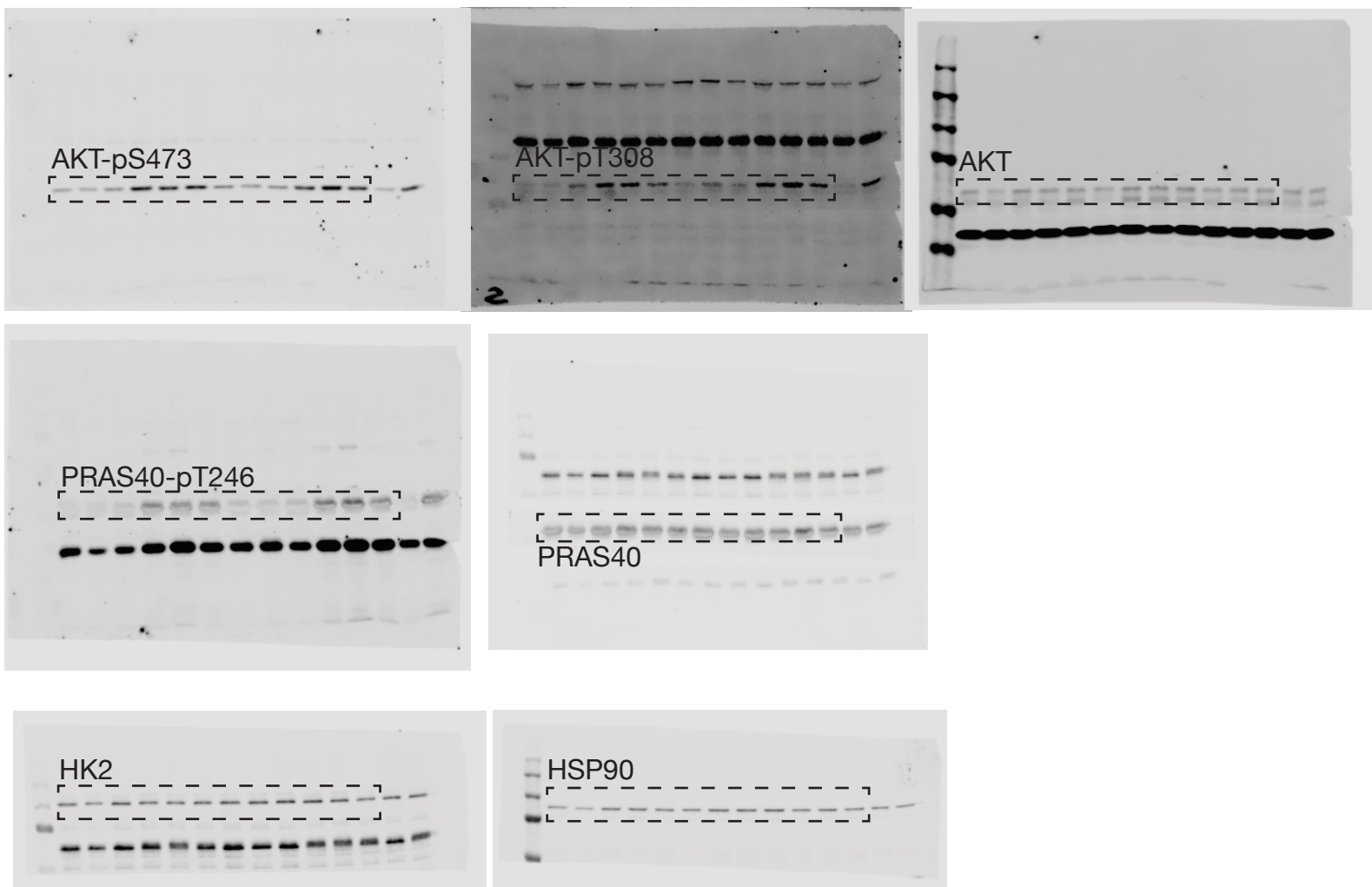

Supplement: Figure 4—figure supplement 1—source data 1. [file elife-85103-fig4-figsupp1-data1.zip › Figure 4 - figure supplement 1 - source data/Figure 4- figure supplement 1D - source data.pdf]

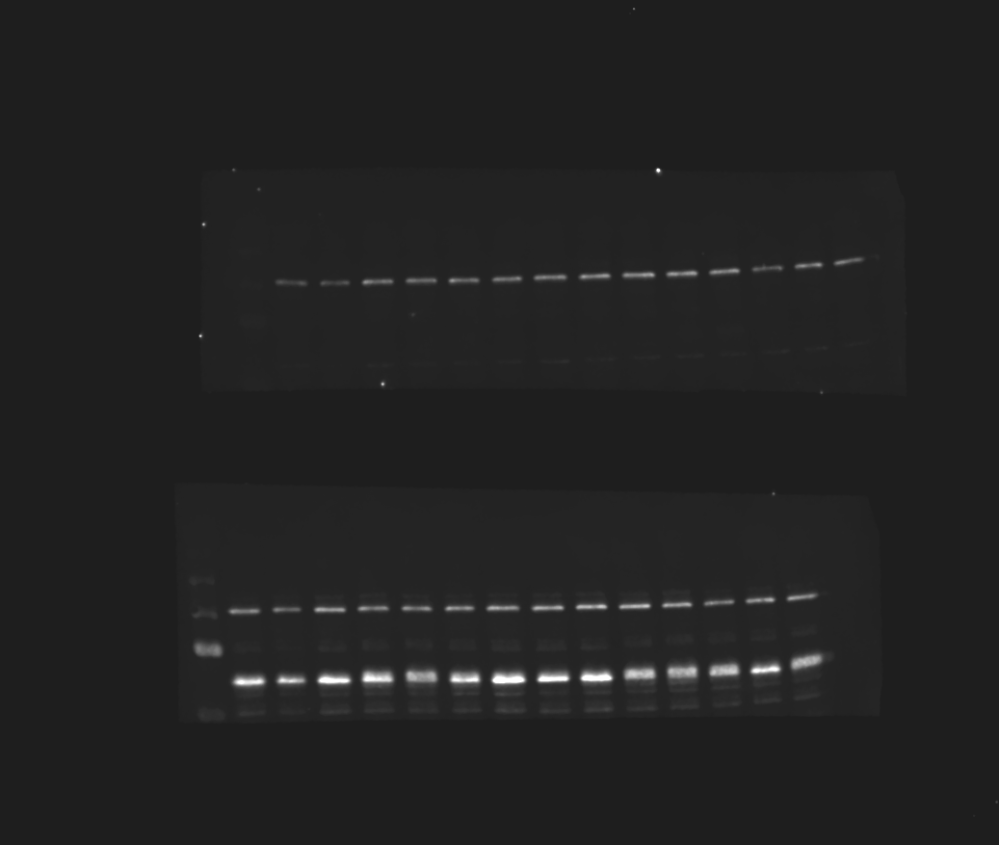

Supplement: Figure 4—figure supplement 1—source data 1. [file elife-85103-fig4-figsupp1-data1.zip › Figure 4 - figure supplement 1 - source data/source data Figure 4 - figure supplement 1D/HK2.tif]

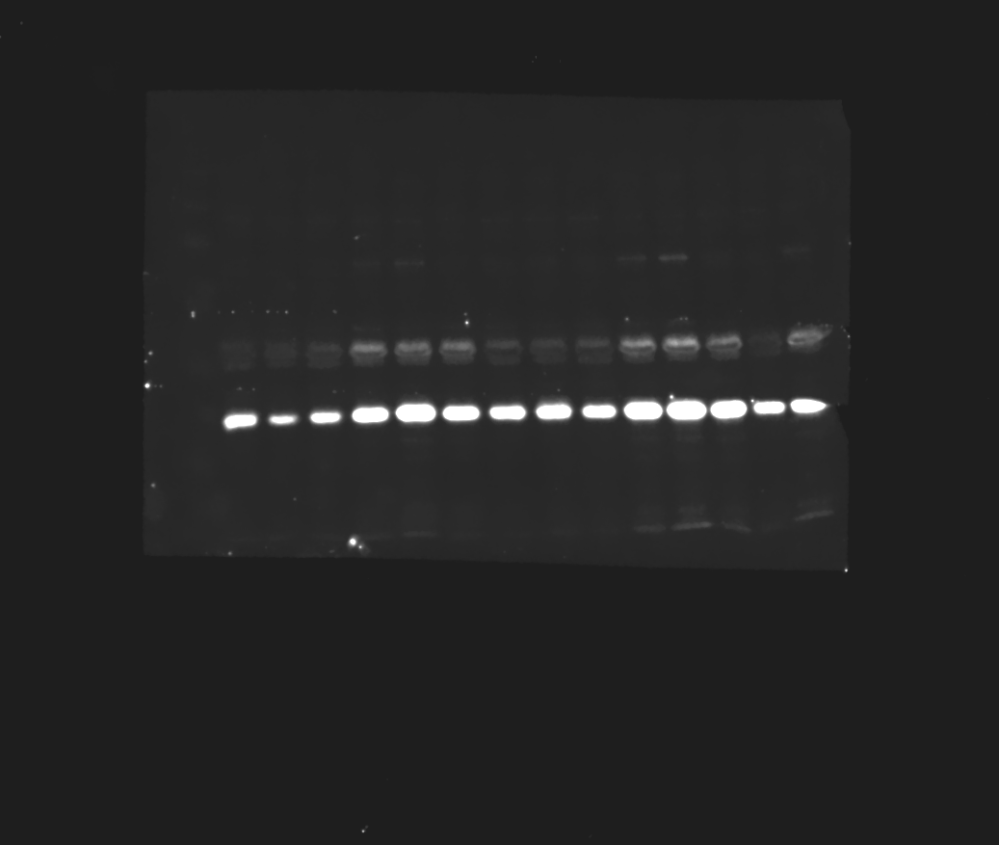

Supplement: Figure 4—figure supplement 1—source data 1. [file elife-85103-fig4-figsupp1-data1.zip › Figure 4 - figure supplement 1 - source data/source data Figure 4 - figure supplement 1D/PRAS40-pT246.tif]

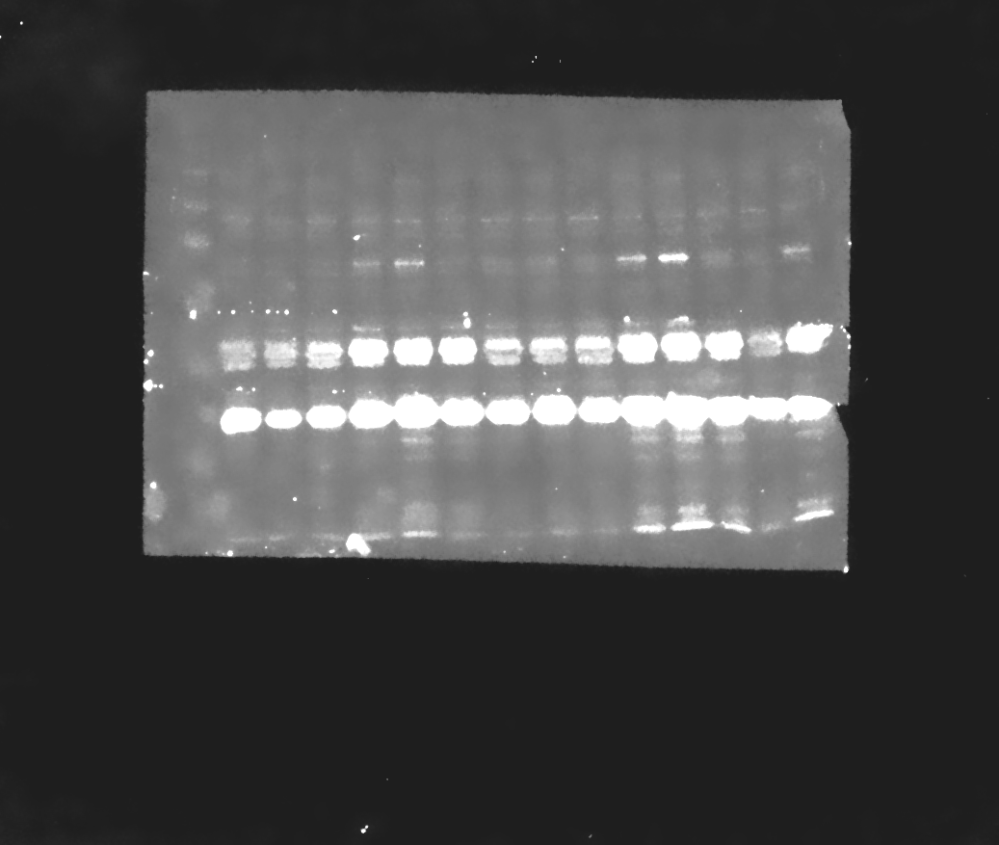

Supplement: Figure 4—figure supplement 1—source data 1. [file elife-85103-fig4-figsupp1-data1.zip › Figure 4 - figure supplement 1 - source data/source data Figure 4 - figure supplement 1D/S6K-pT389.tif]

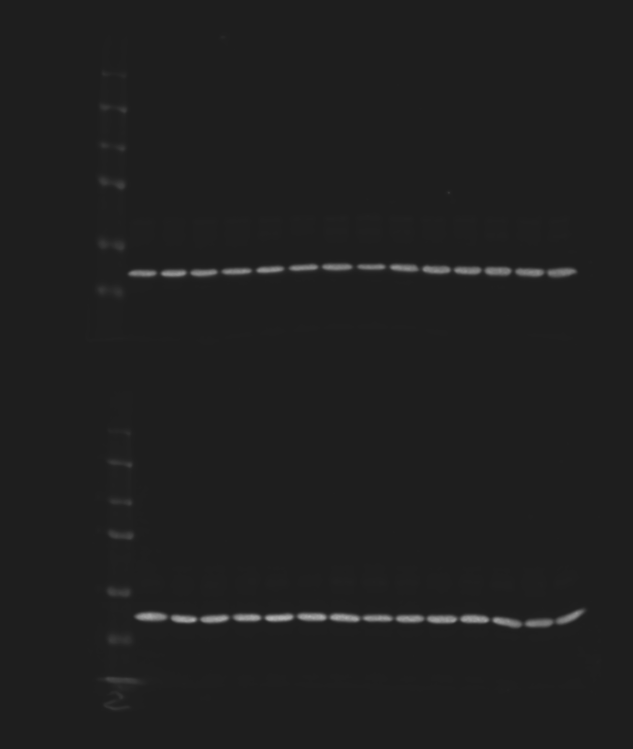

Supplement: Figure 4—figure supplement 1—source data 1. [file elife-85103-fig4-figsupp1-data1.zip › Figure 4 - figure supplement 1 - source data/source data Figure 4 - figure supplement 1D/ACTIN.tif]

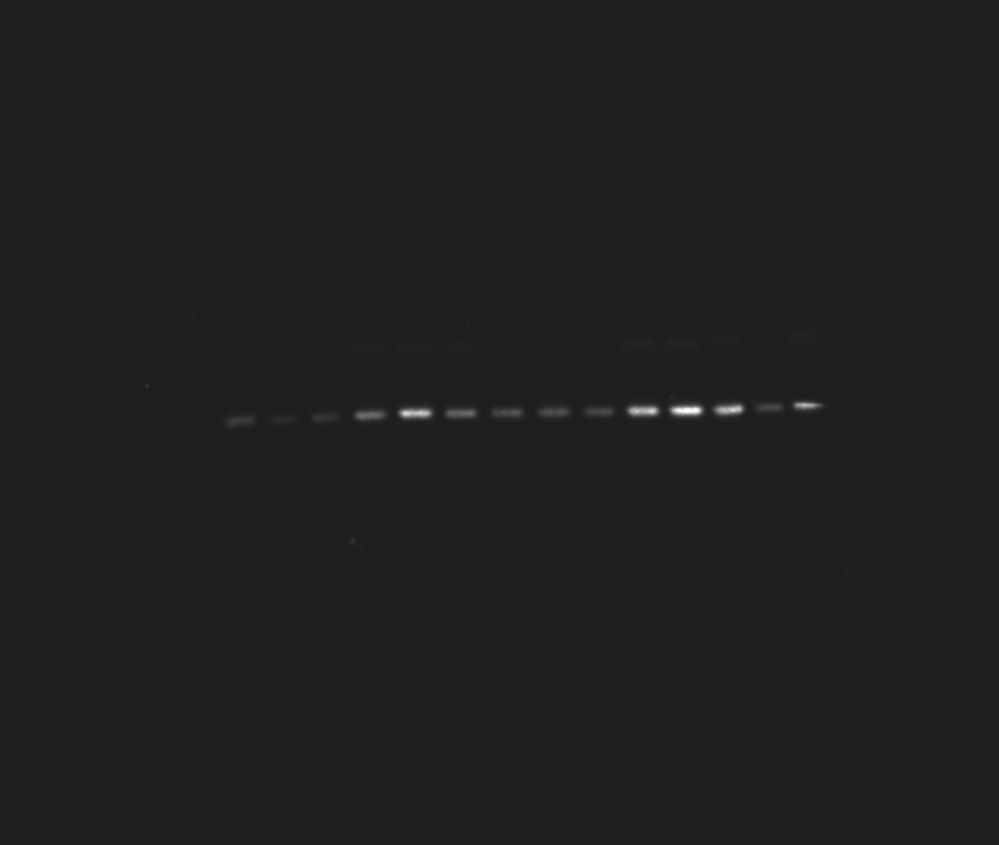

Supplement: Figure 4—figure supplement 1—source data 1. [file elife-85103-fig4-figsupp1-data1.zip › Figure 4 - figure supplement 1 - source data/source data Figure 4 - figure supplement 1D/S6-pS240.tif]

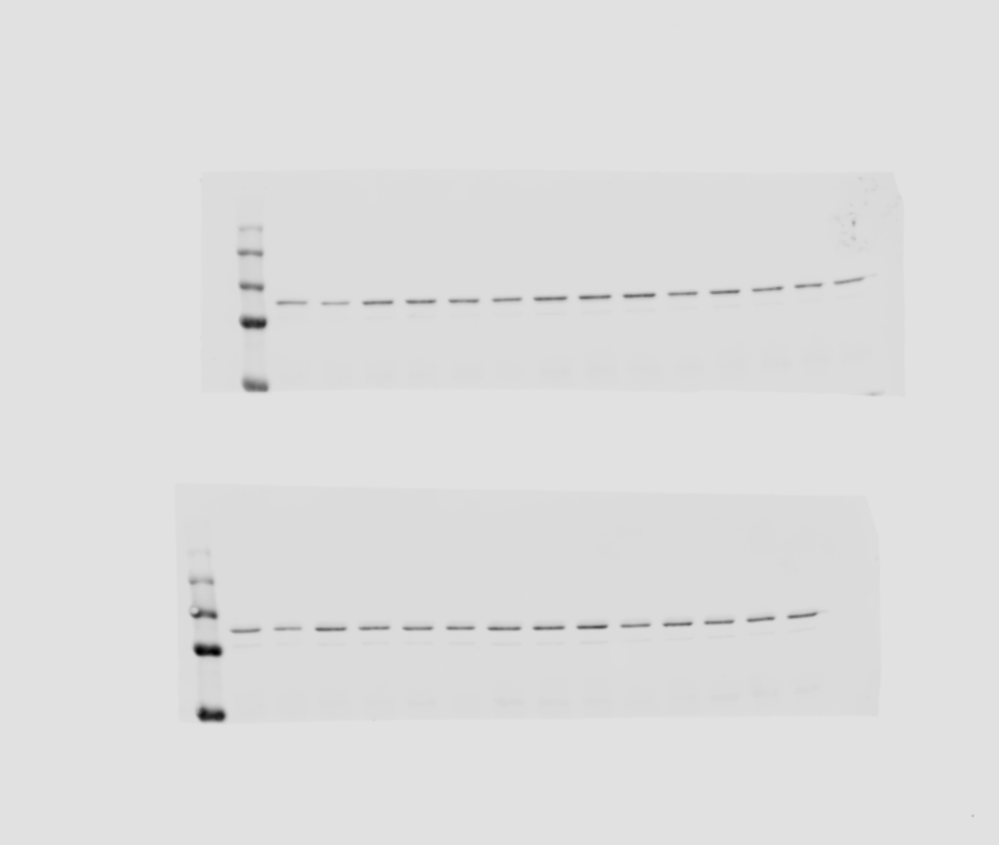

Supplement: Figure 4—figure supplement 1—source data 1. [file elife-85103-fig4-figsupp1-data1.zip › Figure 4 - figure supplement 1 - source data/source data Figure 4 - figure supplement 1D/HSP90.tif]

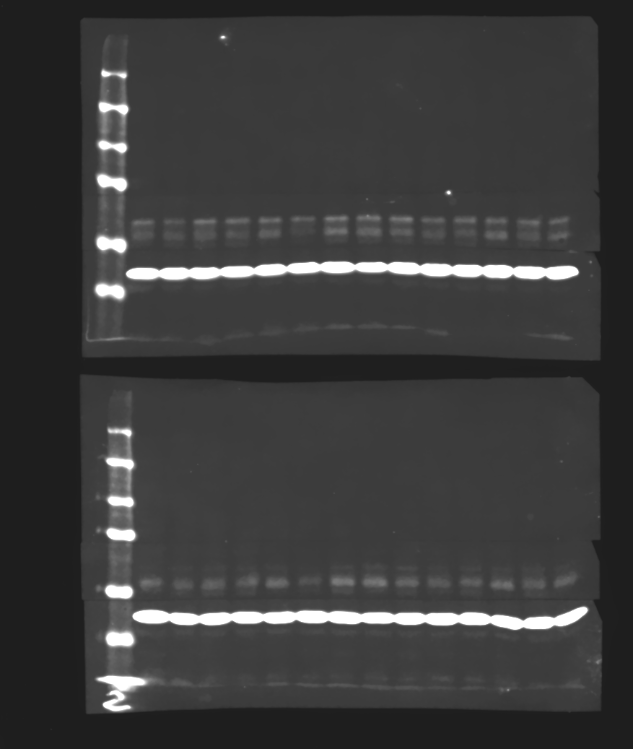

Supplement: Figure 4—figure supplement 1—source data 1. [file elife-85103-fig4-figsupp1-data1.zip › Figure 4 - figure supplement 1 - source data/source data Figure 4 - figure supplement 1D/AKT.tif]

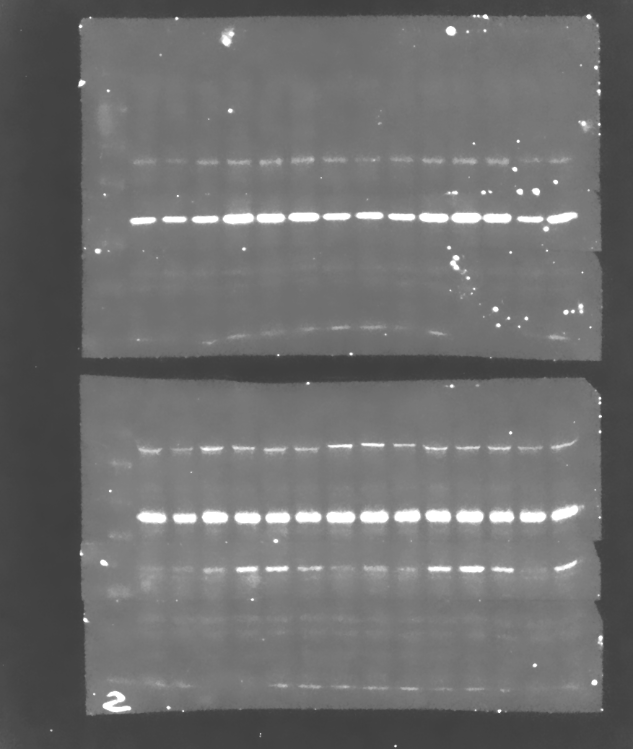

Supplement: Figure 4—figure supplement 1—source data 1. [file elife-85103-fig4-figsupp1-data1.zip › Figure 4 - figure supplement 1 - source data/source data Figure 4 - figure supplement 1D/IR-pY1146_AKT-pT308.tif]

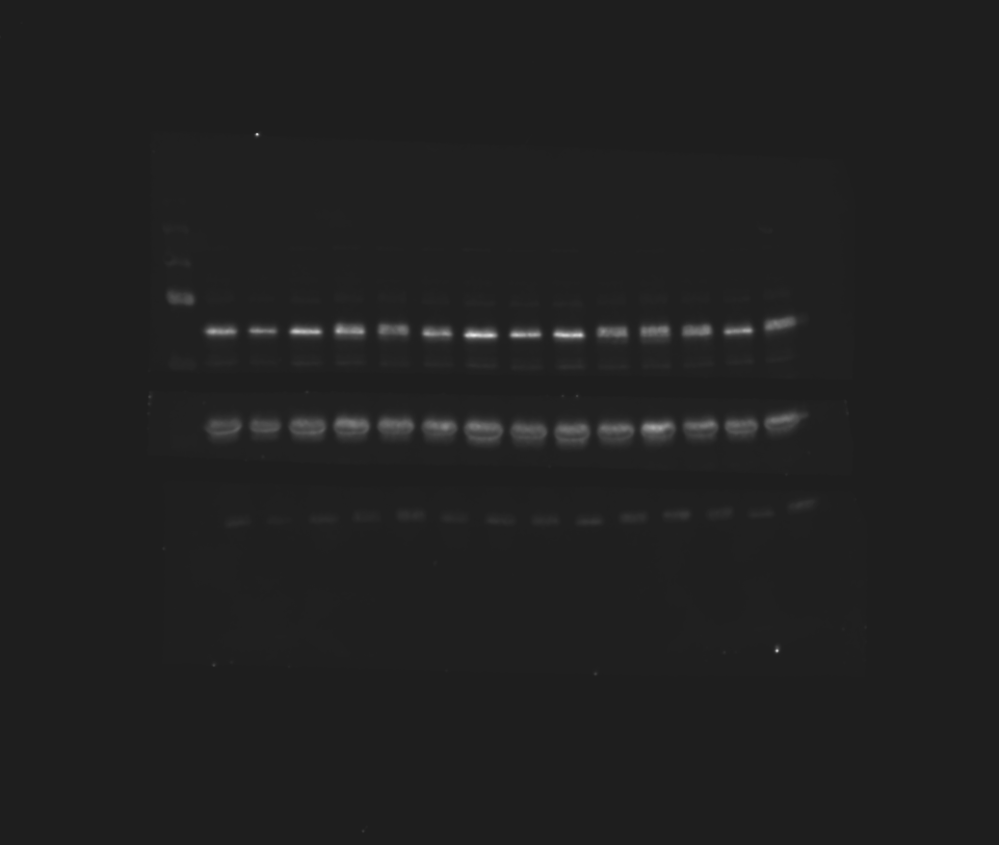

Supplement: Figure 4—figure supplement 1—source data 1. [file elife-85103-fig4-figsupp1-data1.zip › Figure 4 - figure supplement 1 - source data/source data Figure 4 - figure supplement 1D/S6K_PRAS40_S6.tif]

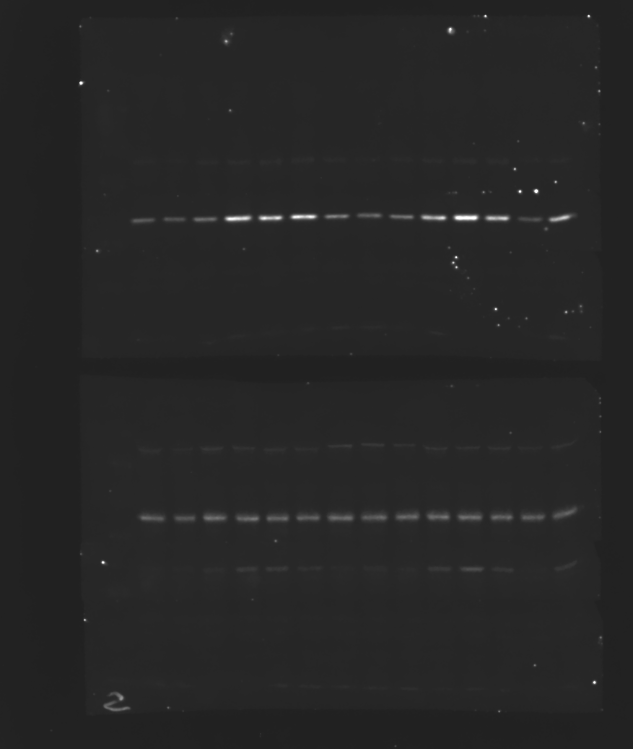

Supplement: Figure 4—figure supplement 1—source data 1. [file elife-85103-fig4-figsupp1-data1.zip › Figure 4 - figure supplement 1 - source data/source data Figure 4 - figure supplement 1D/AKT-pS473_IR.tif]

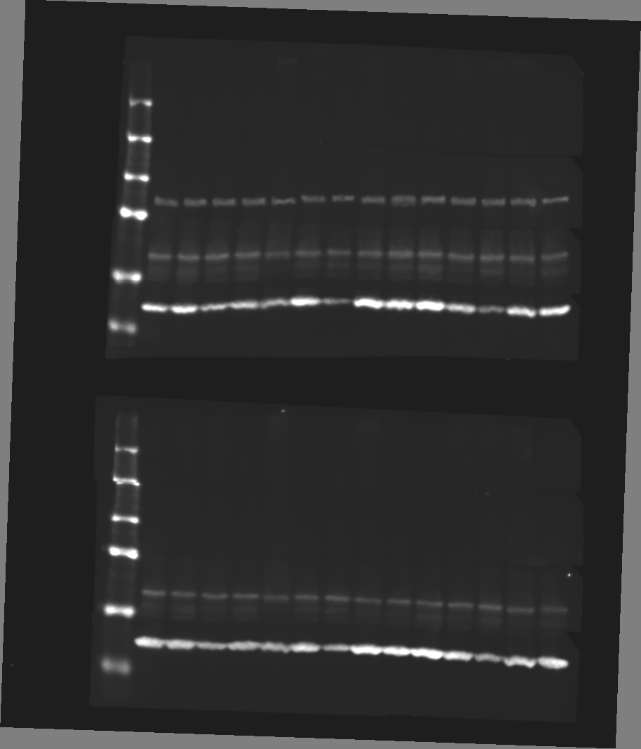

Supplement: Figure 4—figure supplement 1—source data 1. [file elife-85103-fig4-figsupp1-data1.zip › Figure 4 - figure supplement 1 - source data/source data Figure 4 - figure supplement 1B-C/BAT_AKT.tif]

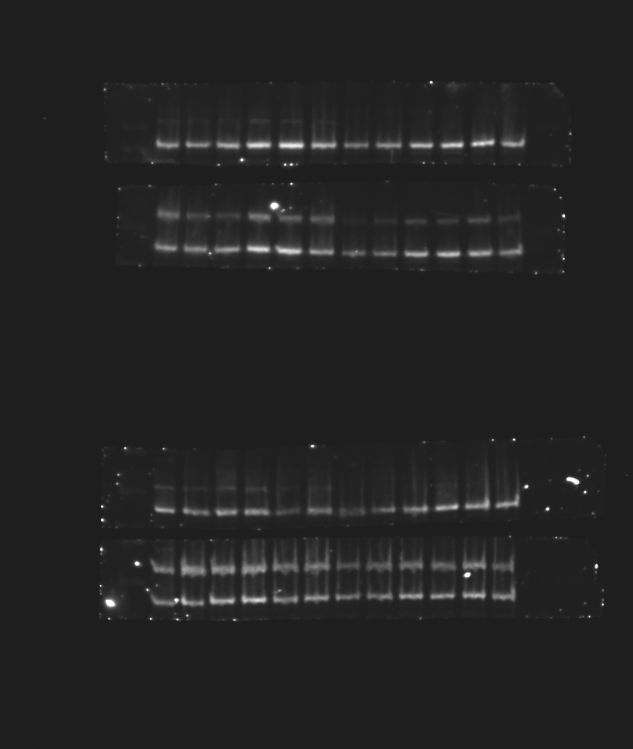

Supplement: Figure 4—figure supplement 1—source data 1. [file elife-85103-fig4-figsupp1-data1.zip › Figure 4 - figure supplement 1 - source data/source data Figure 4 - figure supplement 1B-C/sWAT_CALX.tif]

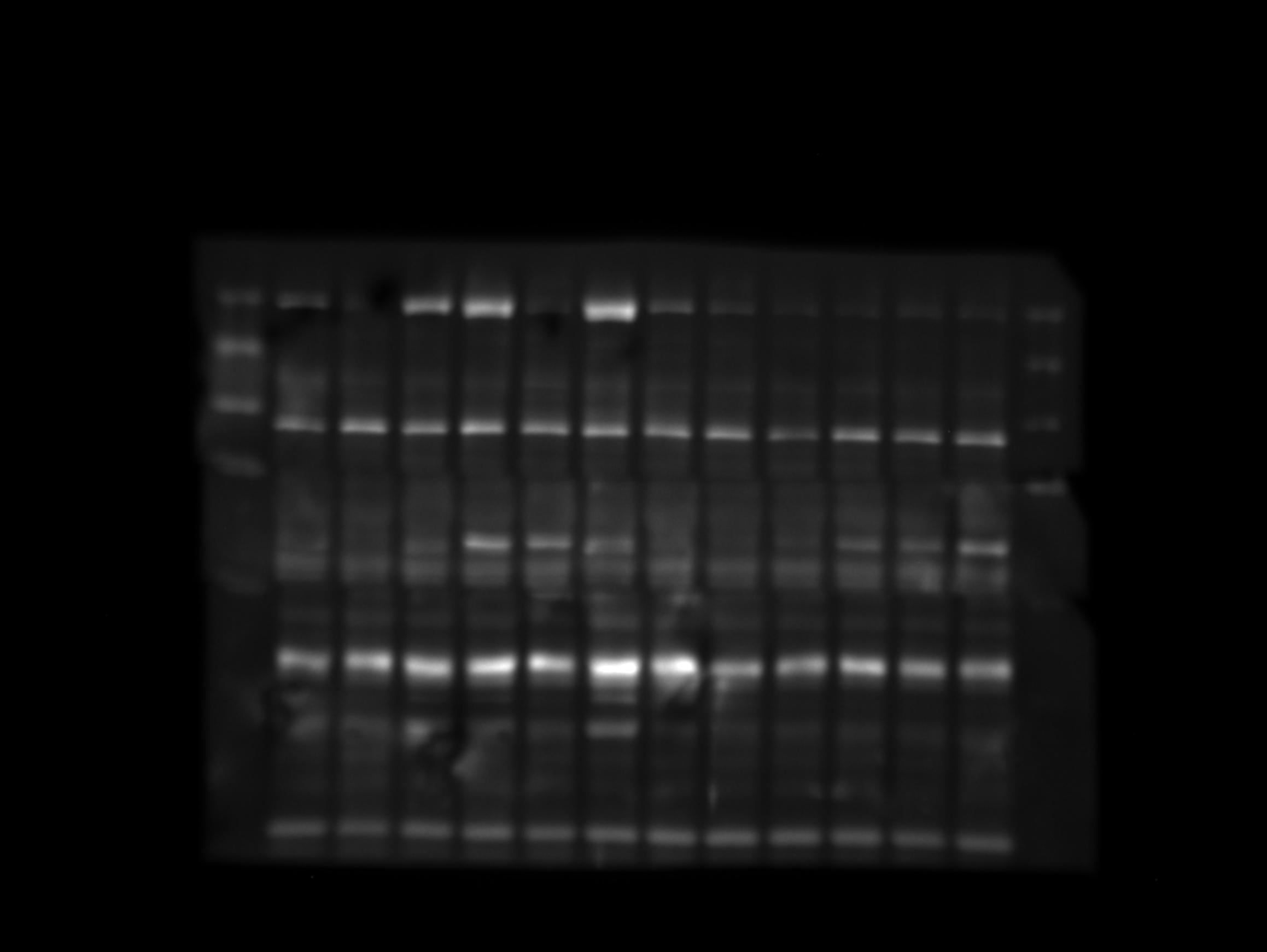

Supplement: Figure 4—figure supplement 1—source data 1. [file elife-85103-fig4-figsupp1-data1.zip › Figure 4 - figure supplement 1 - source data/source data Figure 4 - figure supplement 1B-C/sWAT_CALX_pT308_PRAS40.tif]

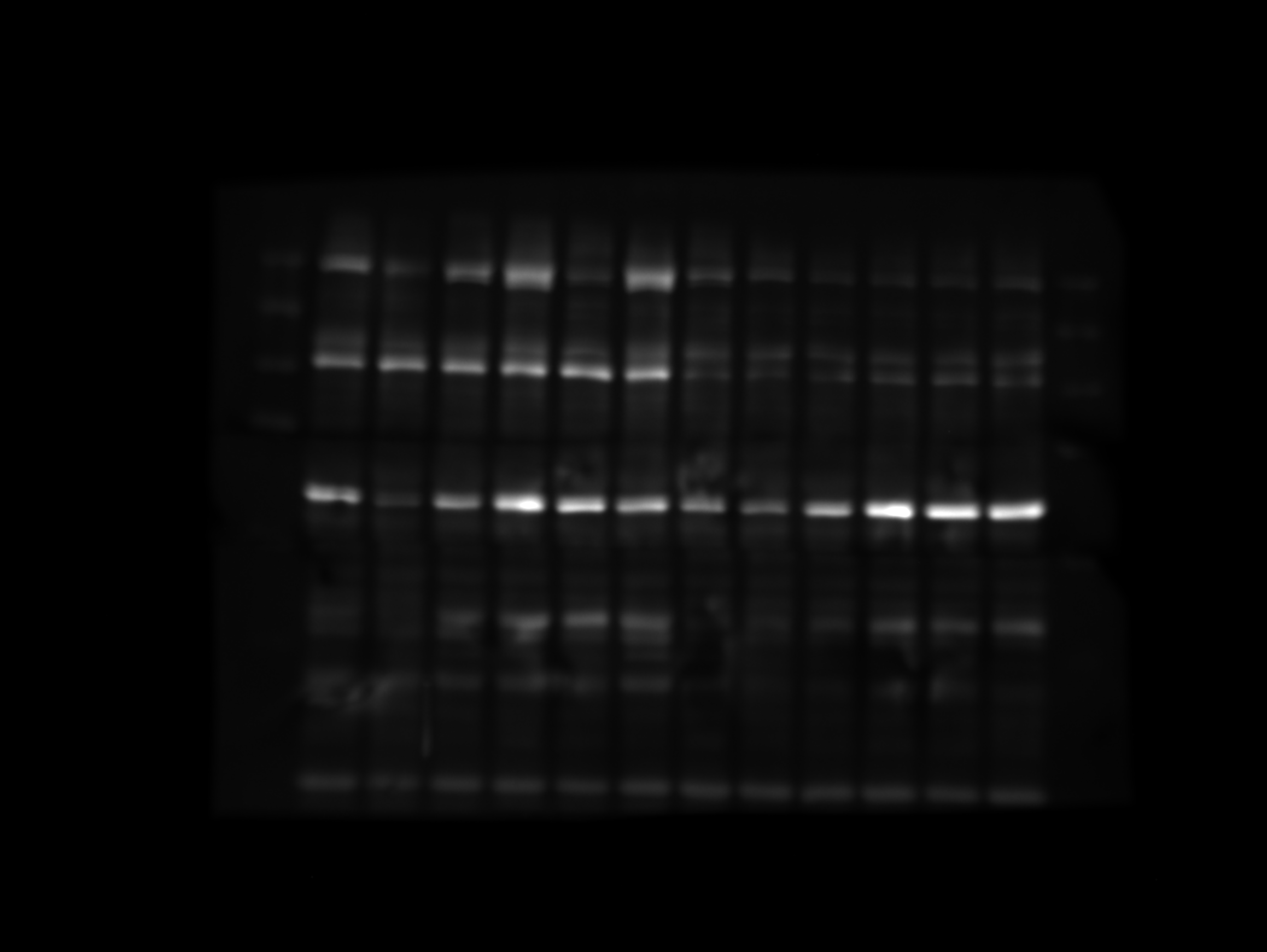

Supplement: Figure 4—figure supplement 1—source data 1. [file elife-85103-fig4-figsupp1-data1.zip › Figure 4 - figure supplement 1 - source data/source data Figure 4 - figure supplement 1B-C/sWAT_HK2_pS473_pPRAS40.tif]

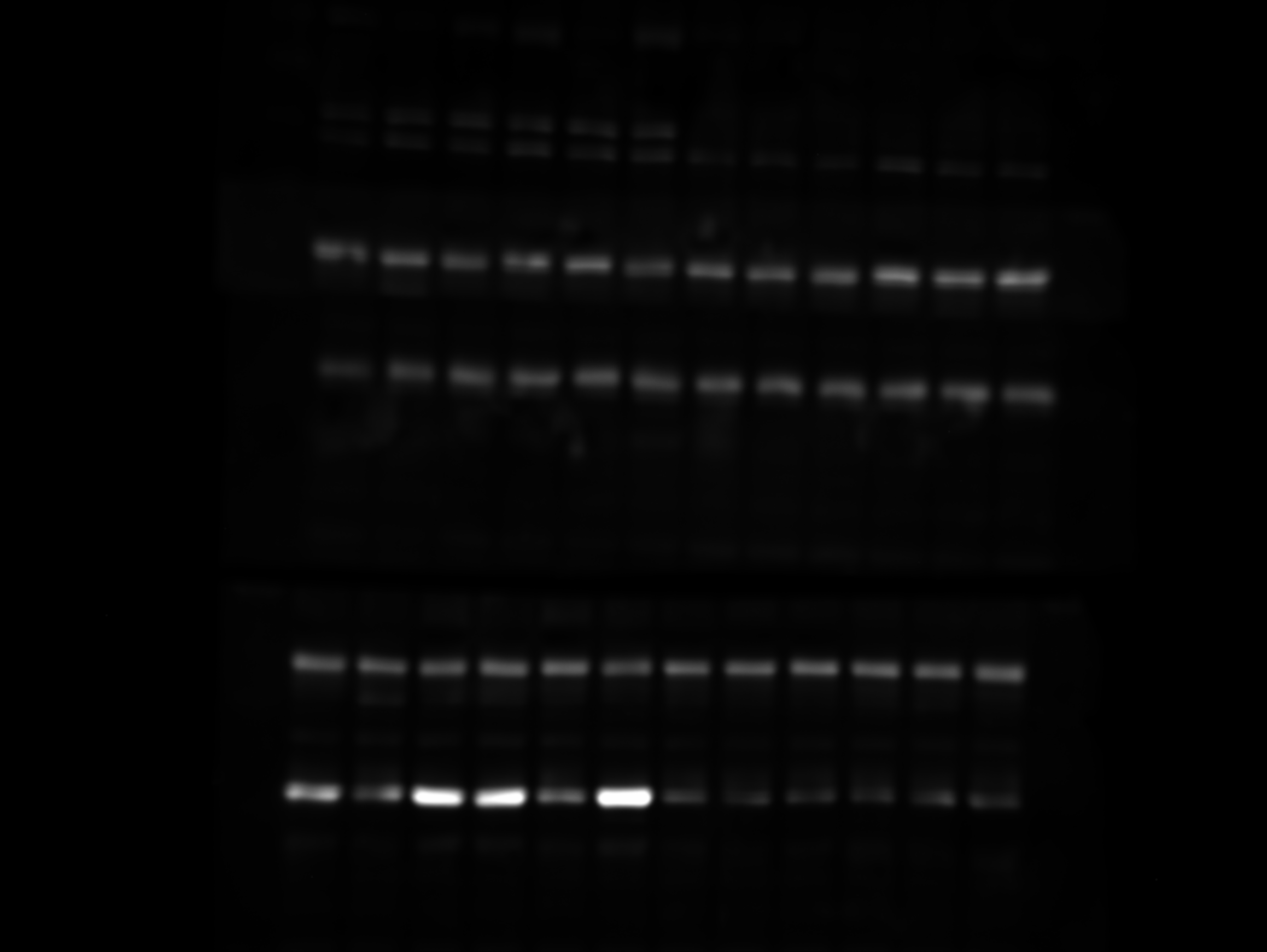

Supplement: Figure 4—figure supplement 1—source data 1. [file elife-85103-fig4-figsupp1-data1.zip › Figure 4 - figure supplement 1 - source data/source data Figure 4 - figure supplement 1B-C/sWAT_CALX_AKT_PRAS40_AKT_GAP43-sub0-As-Displayed.tif]

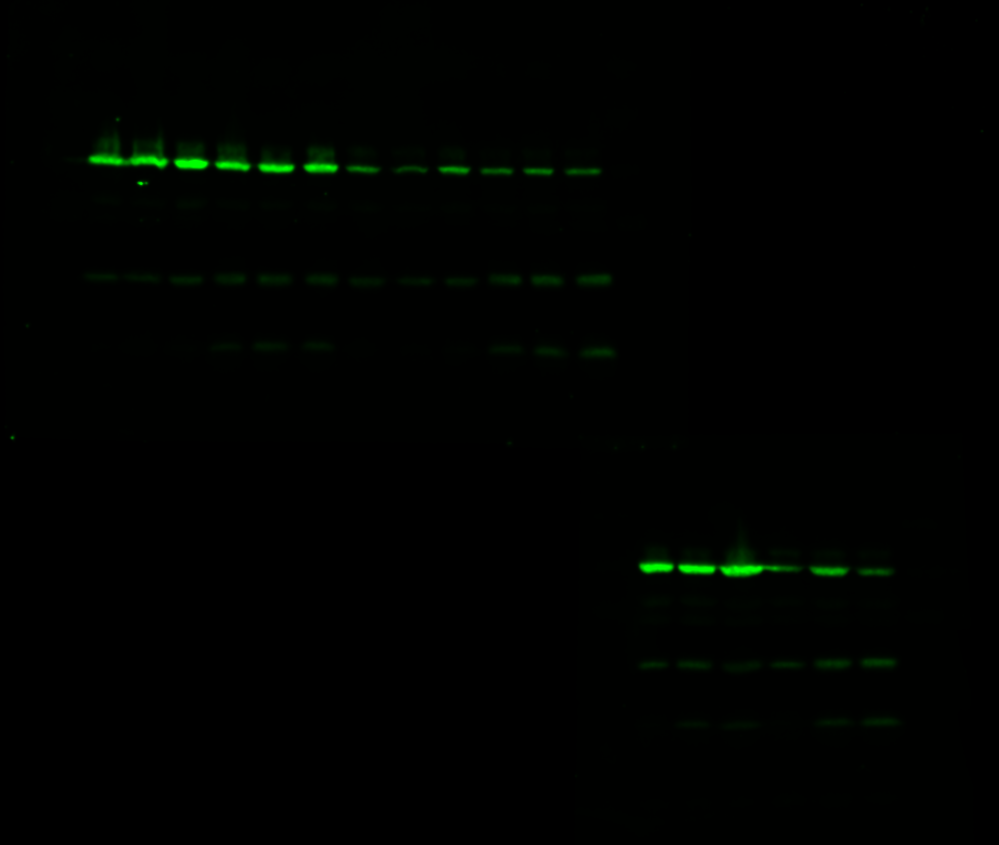

Supplement: Figure 4—figure supplement 1—source data 1. [file elife-85103-fig4-figsupp1-data1.zip › Figure 4 - figure supplement 1 - source data/source data Figure 4 - figure supplement 1B-C/BAT_HK2_2.tif]

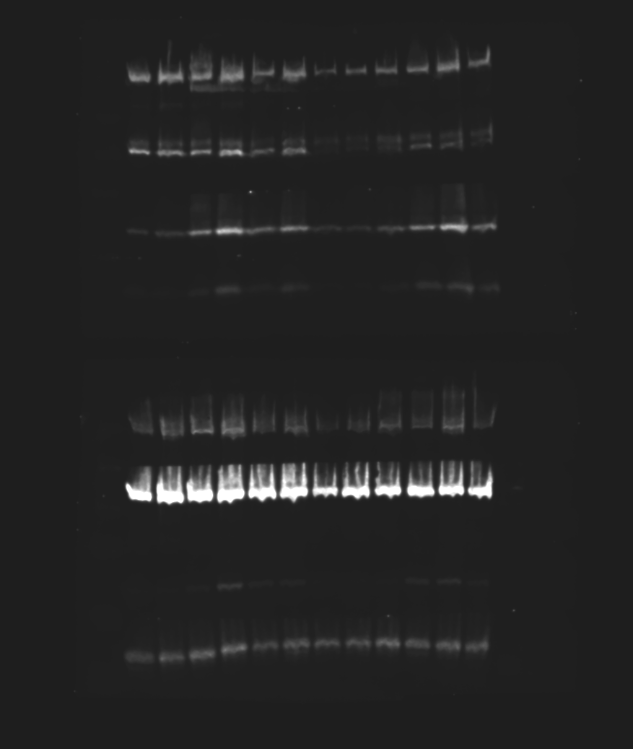

Supplement: Figure 4—figure supplement 1—source data 1. [file elife-85103-fig4-figsupp1-data1.zip › Figure 4 - figure supplement 1 - source data/source data Figure 4 - figure supplement 1B-C/sWAT_HK2_AKT-pS473_PRAS40-pT246_AKT-pT308_PRAS40.tif]

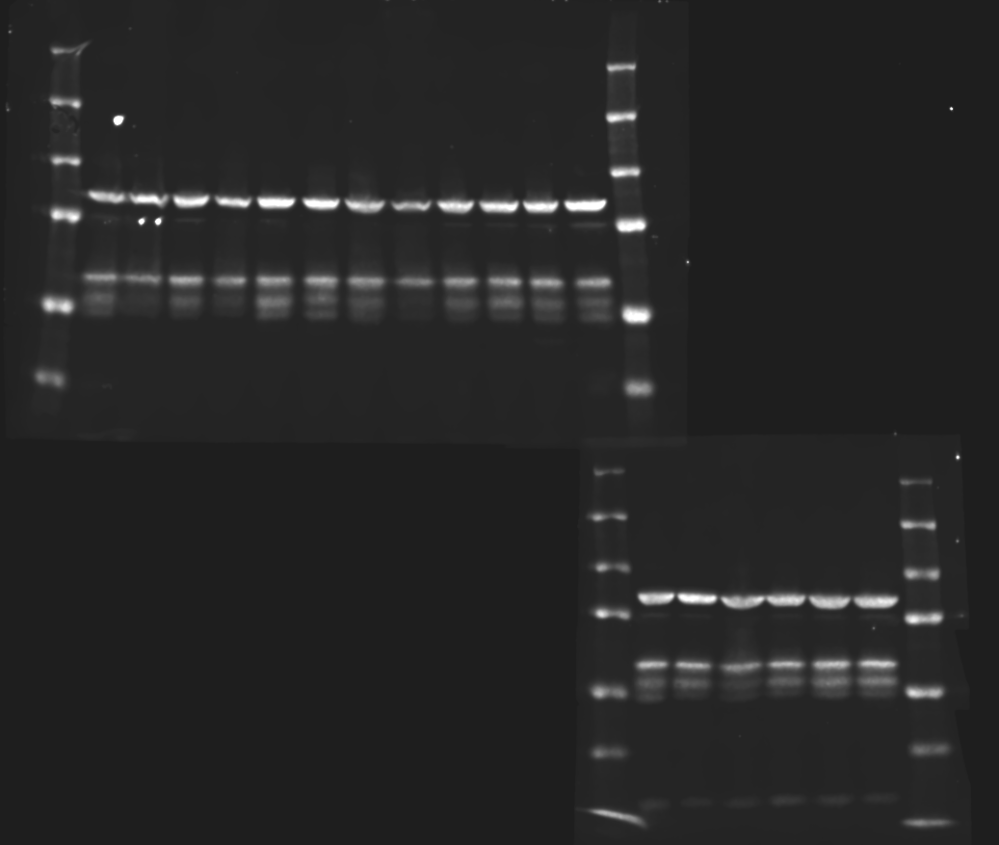

Supplement: Figure 4—figure supplement 1—source data 1. [file elife-85103-fig4-figsupp1-data1.zip › Figure 4 - figure supplement 1 - source data/source data Figure 4 - figure supplement 1B-C/BAT_AKT_PRAS40_2.tif]

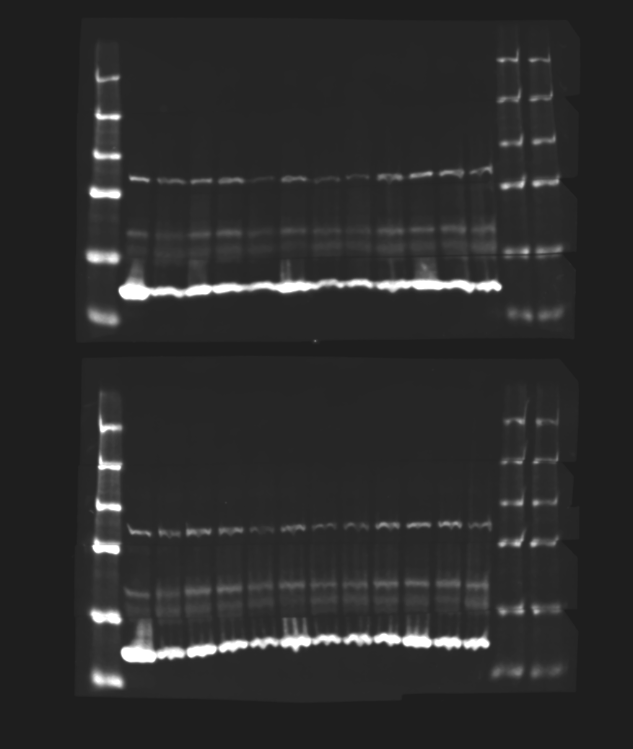

Supplement: Figure 4—figure supplement 1—source data 1. [file elife-85103-fig4-figsupp1-data1.zip › Figure 4 - figure supplement 1 - source data/source data Figure 4 - figure supplement 1B-C/sWAT_AKT.tif]

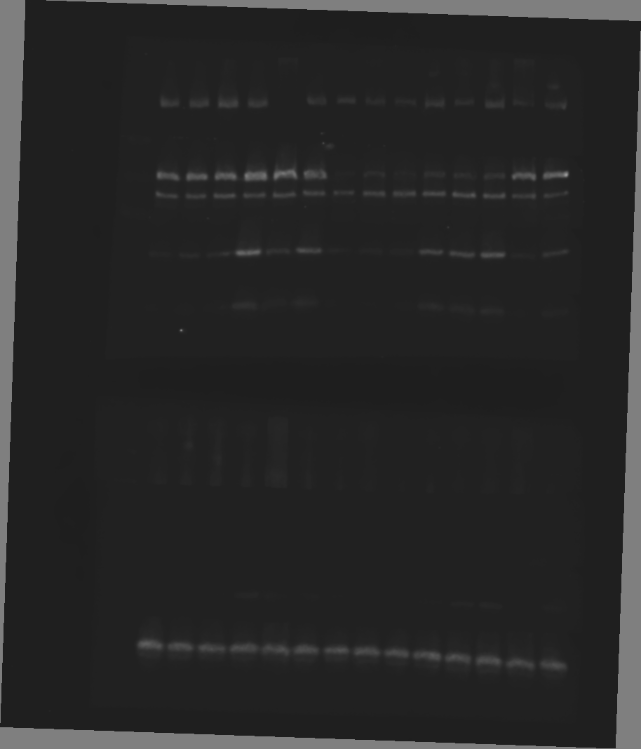

Supplement: Figure 4—figure supplement 1—source data 1. [file elife-85103-fig4-figsupp1-data1.zip › Figure 4 - figure supplement 1 - source data/source data Figure 4 - figure supplement 1B-C/BAT_HK2_AKT-pS473_PRAS40-pT246_CALX.tif]

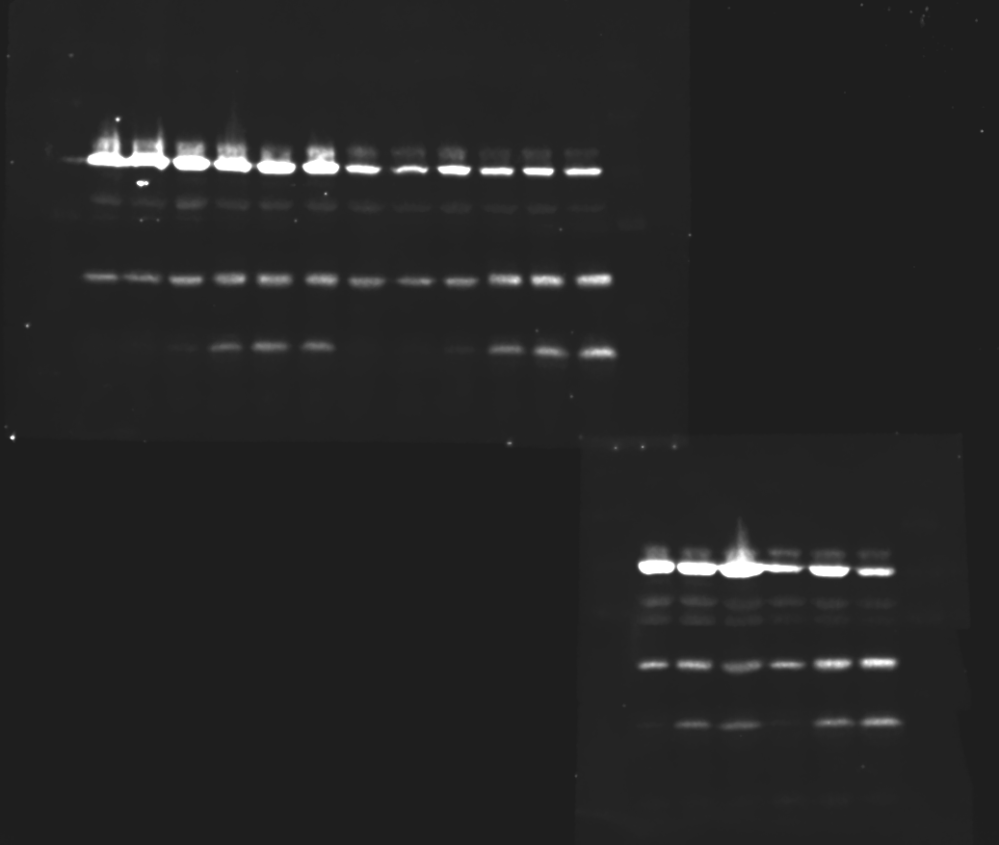

Supplement: Figure 4—figure supplement 1—source data 1. [file elife-85103-fig4-figsupp1-data1.zip › Figure 4 - figure supplement 1 - source data/source data Figure 4 - figure supplement 1B-C/BAT_HK2_AKT-pS473_PRAS40-pT246_CALX_2.tif]

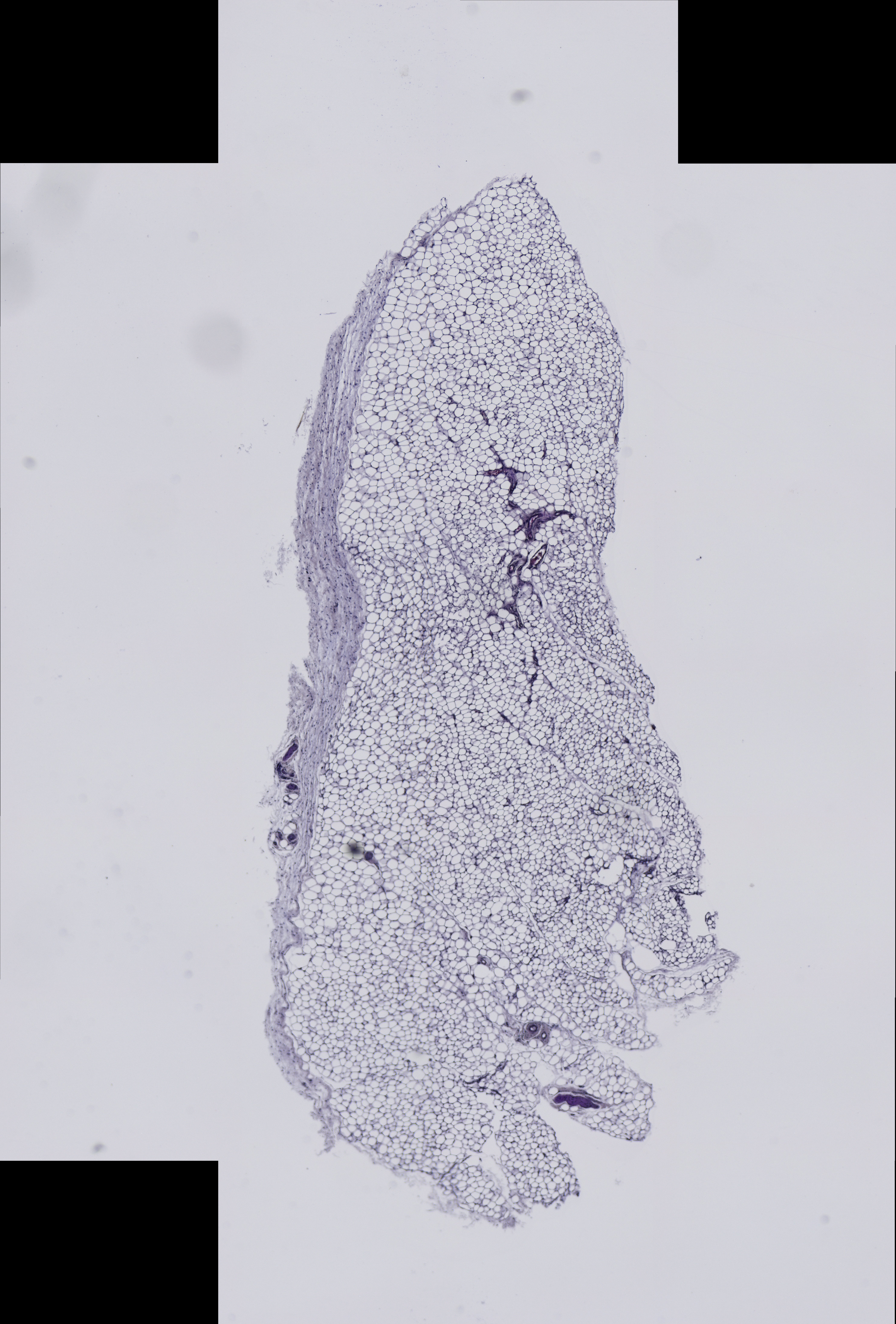

Supplement: Figure 4—figure supplement 2—source data 1. [file elife-85103-fig4-figsupp2-data1.zip › Figure 4 - figure supplement 2 - source data/source data figure 4 - figure supplement 2E/sWAT_16131_KO.jpg]

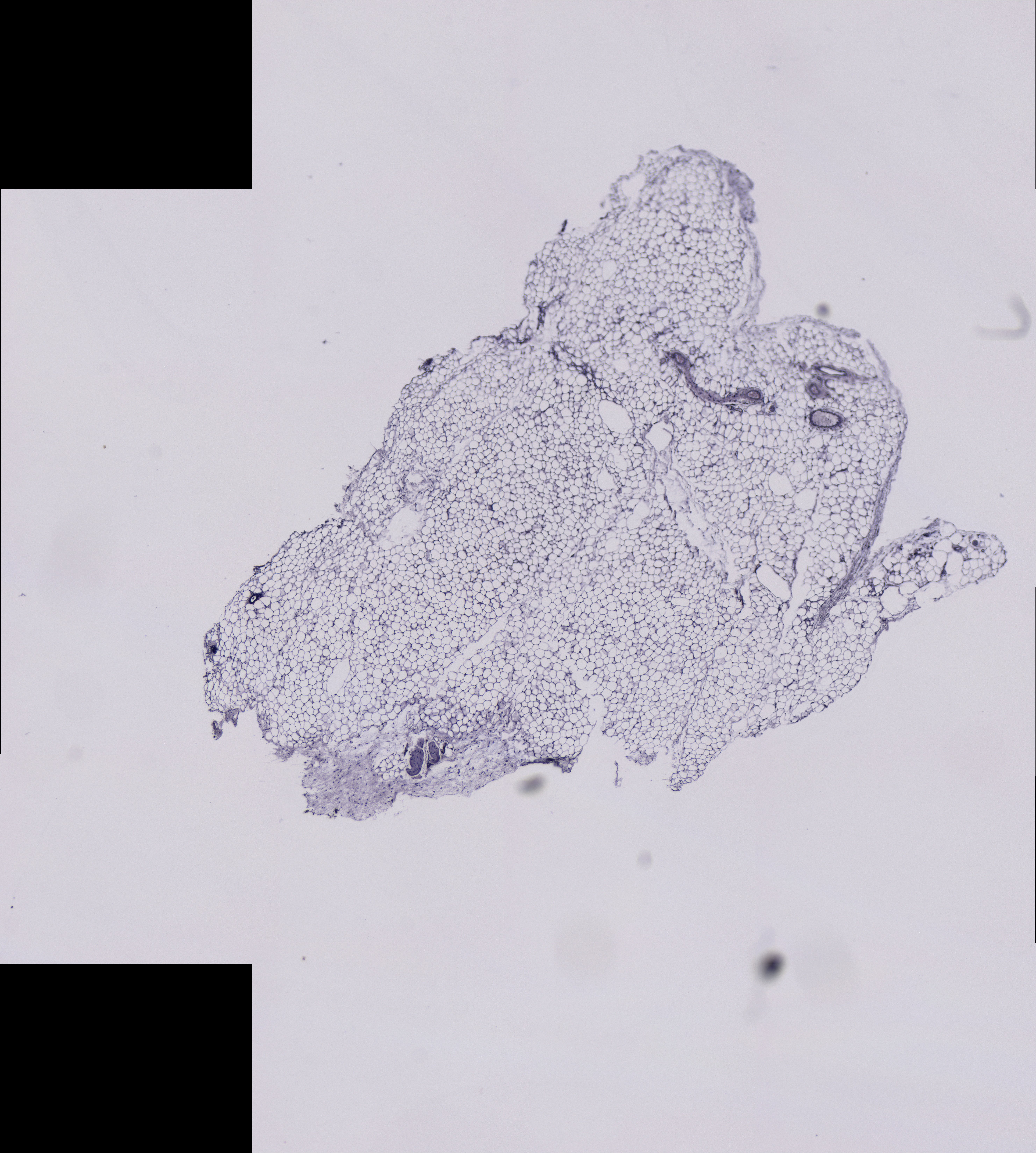

Supplement: Figure 4—figure supplement 2—source data 1. [file elife-85103-fig4-figsupp2-data1.zip › Figure 4 - figure supplement 2 - source data/source data figure 4 - figure supplement 2E/sWAT_16129_control.jpg]

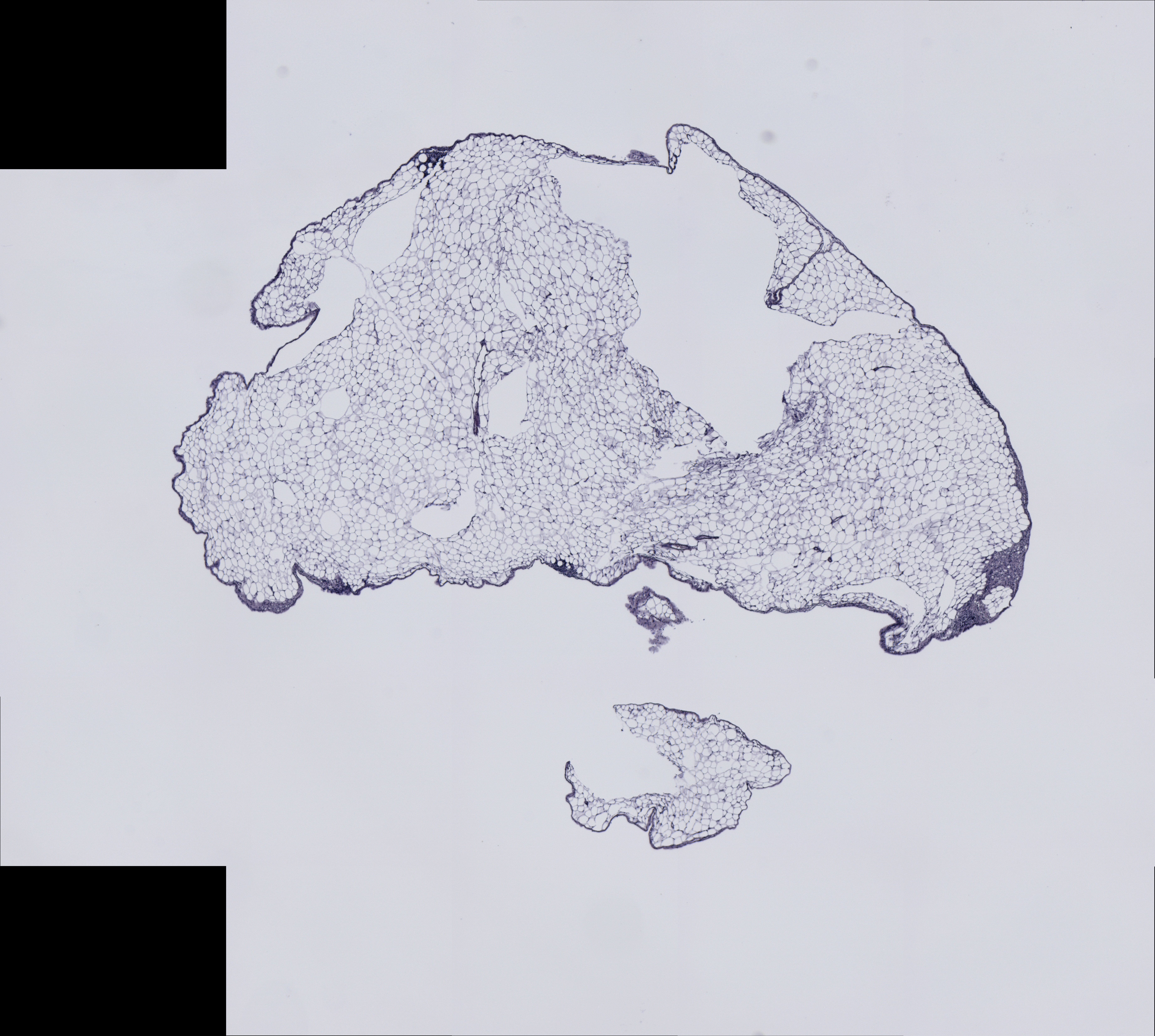

Supplement: Figure 4—figure supplement 2—source data 1. [file elife-85103-fig4-figsupp2-data1.zip › Figure 4 - figure supplement 2 - source data/source data figure 4 - figure supplement 2E/vWAT_16131_KO.jpg]

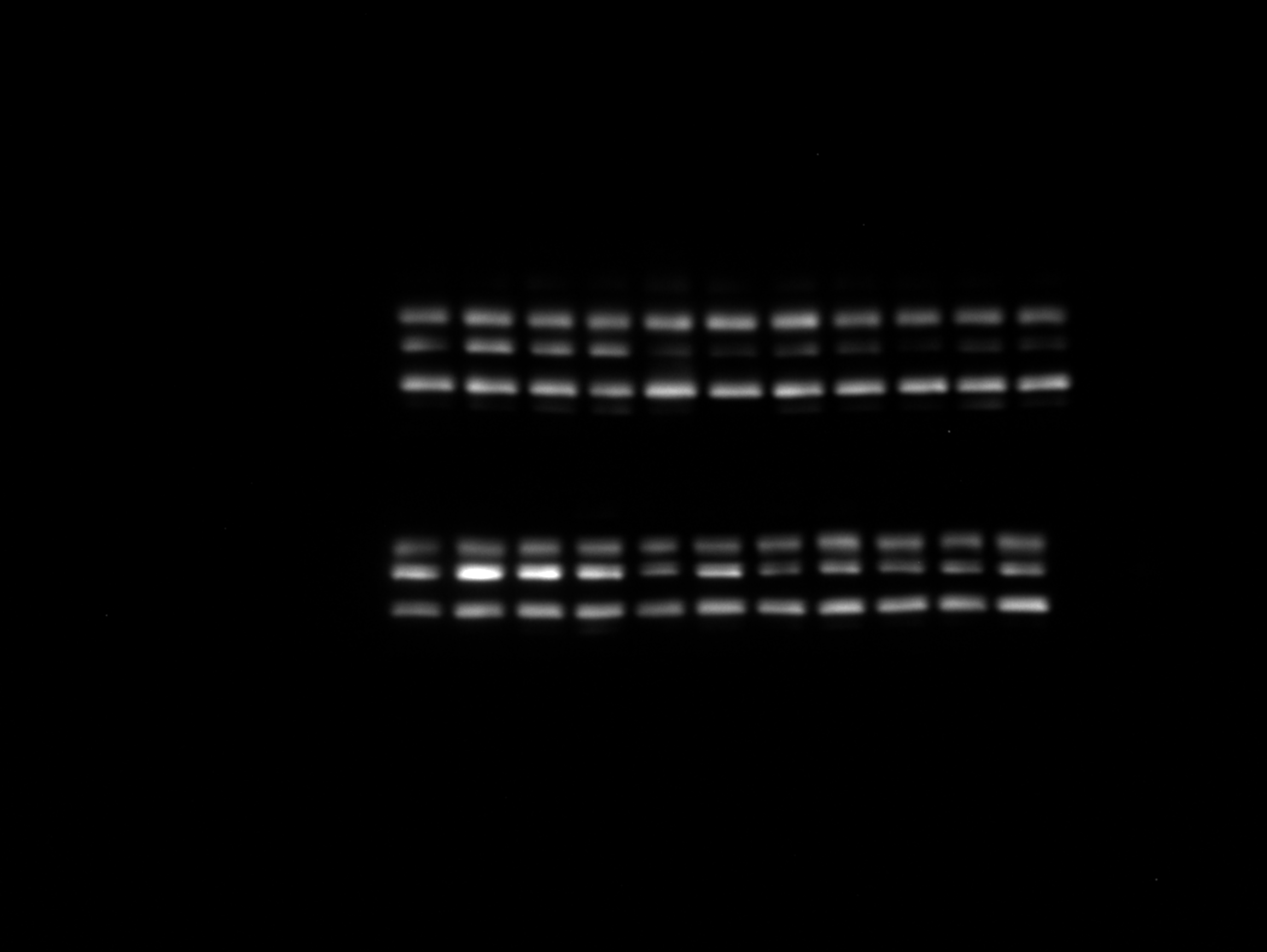

Supplement: Figure 4—figure supplement 3—source data 1. [file elife-85103-fig4-figsupp3-data1.zip › Figure 4 - figure supplement 3 - source data/source data Figure 4 - figure supplement 3/MS65_220406_eWAT_sWAT_HK2_CALX_2.tif]

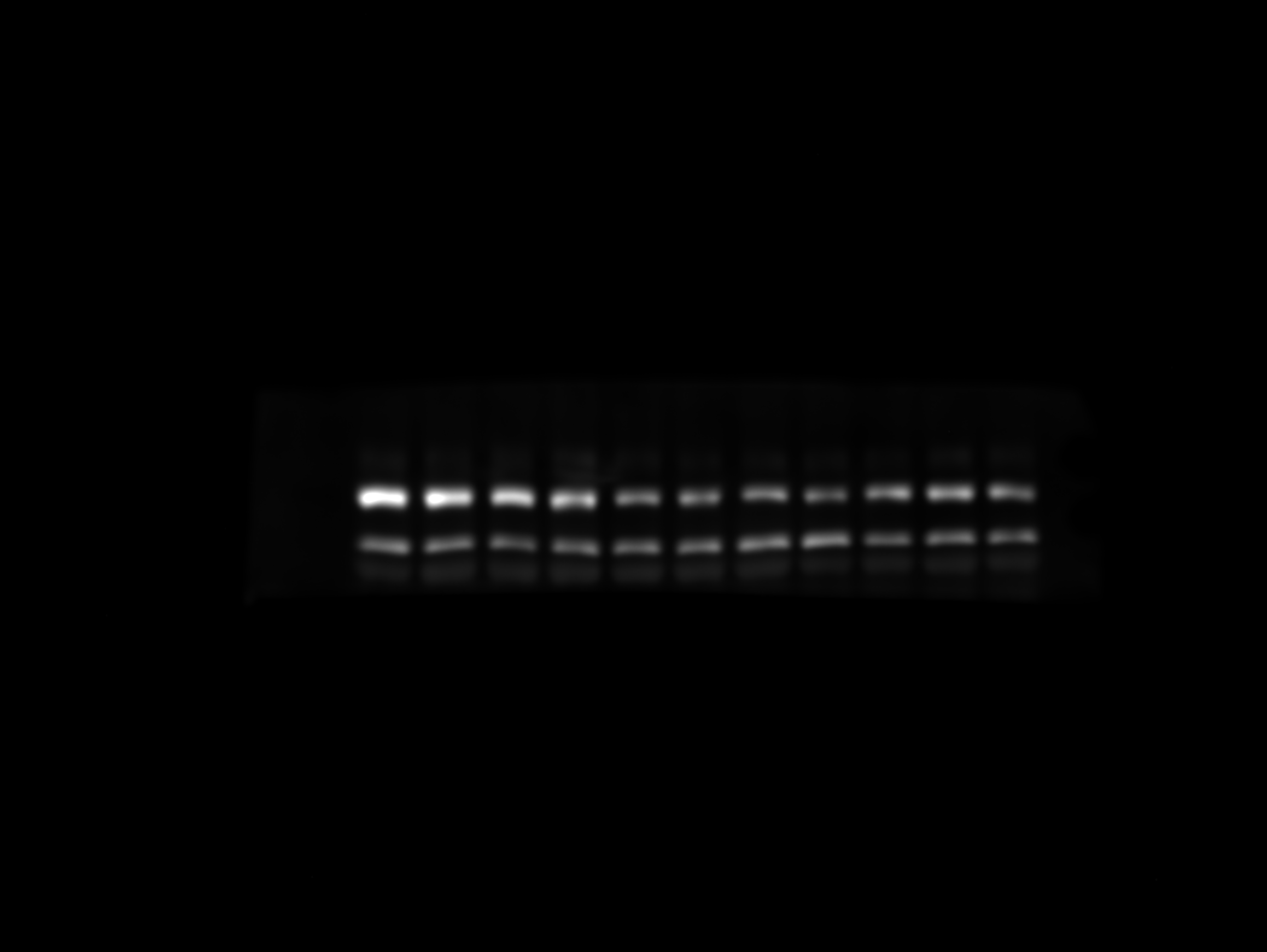

Supplement: Figure 4—figure supplement 3—source data 1. [file elife-85103-fig4-figsupp3-data1.zip › Figure 4 - figure supplement 3 - source data/source data Figure 4 - figure supplement 3/MS65_220502_BAT_HK2_CALX_2.tif]

Figure S8A

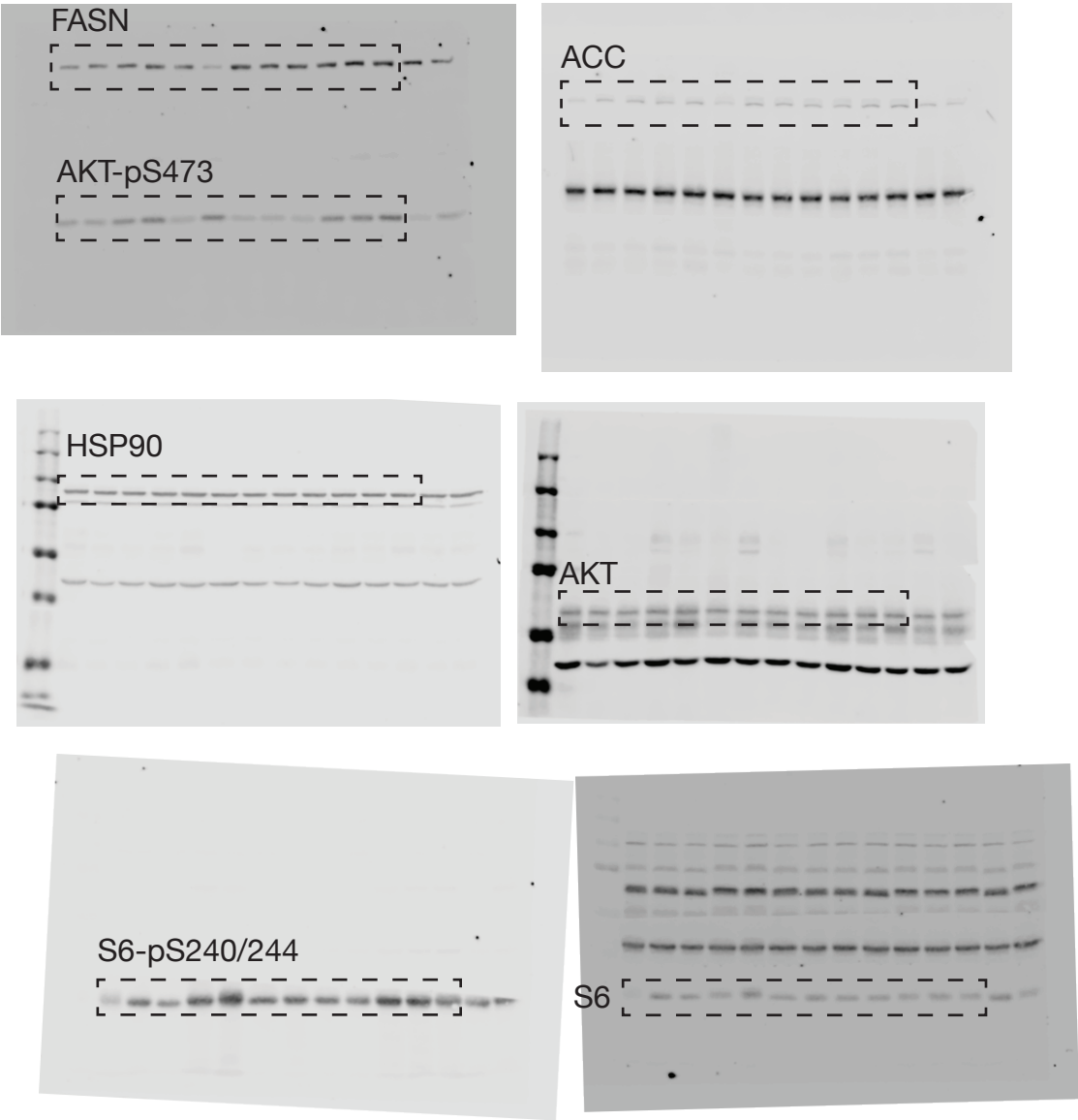

Supplement: Figure 5—figure supplement 1—source data 1. [file elife-85103-fig5-figsupp1-data1.zip › Figure 5 - figure supplement 1 - source data/Figure 5 - figure supplement 1C - source data.pdf]

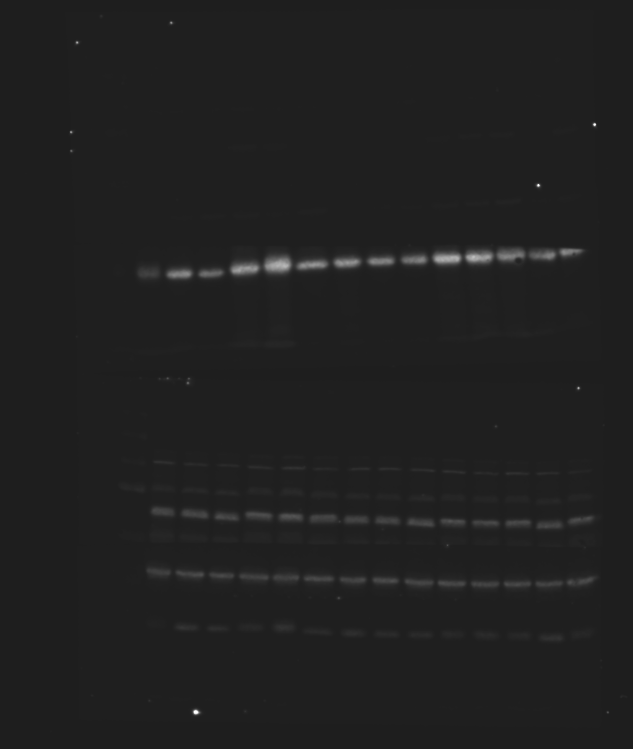

Supplement: Figure 5—figure supplement 1—source data 1. [file elife-85103-fig5-figsupp1-data1.zip › Figure 5 - figure supplement 1 - source data/source data Figure 5 - figure supplement 1C/S6-pS240_S6K_PRAS40_S6.tif]

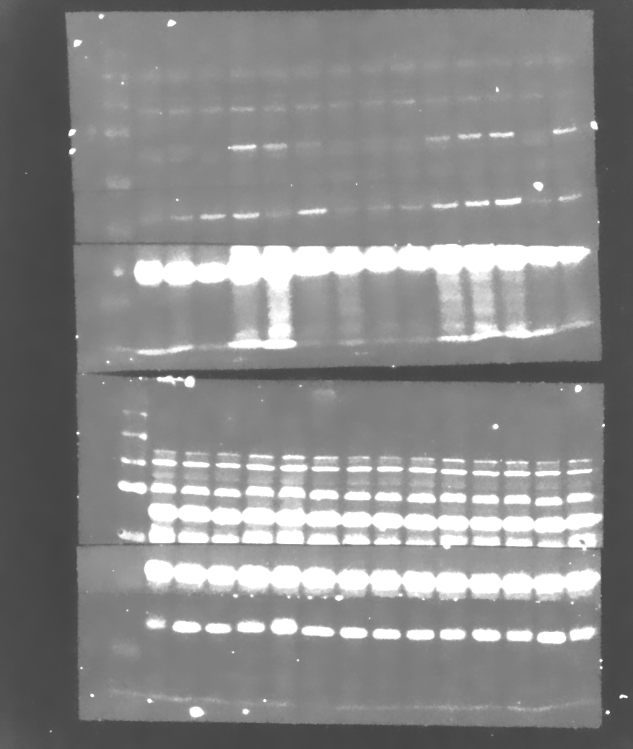

Supplement: Figure 5—figure supplement 1—source data 1. [file elife-85103-fig5-figsupp1-data1.zip › Figure 5 - figure supplement 1 - source data/source data Figure 5 - figure supplement 1C/S6K-pT389_PRAS40-pT246.tif]

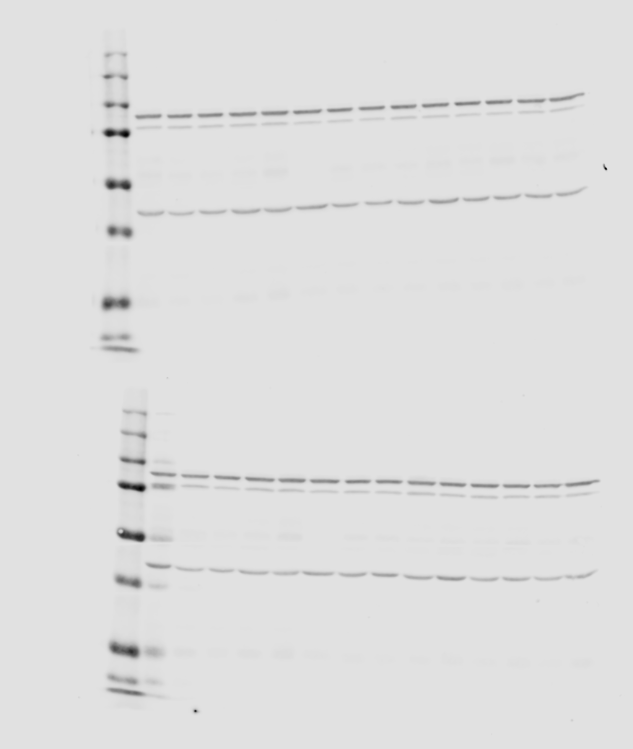

Supplement: Figure 5—figure supplement 1—source data 1. [file elife-85103-fig5-figsupp1-data1.zip › Figure 5 - figure supplement 1 - source data/source data Figure 5 - figure supplement 1C/HSP90.tif]

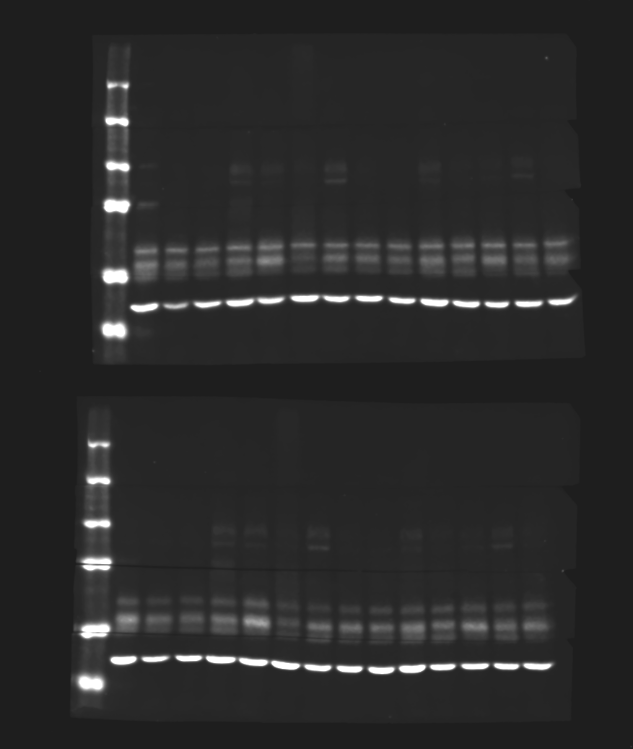

Supplement: Figure 5—figure supplement 1—source data 1. [file elife-85103-fig5-figsupp1-data1.zip › Figure 5 - figure supplement 1 - source data/source data Figure 5 - figure supplement 1C/AKT.tif]

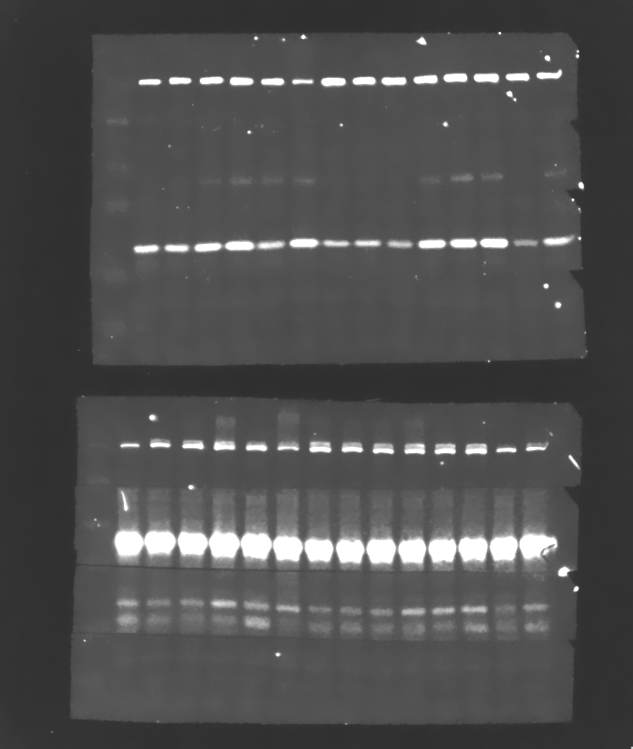

Supplement: Figure 5—figure supplement 1—source data 1. [file elife-85103-fig5-figsupp1-data1.zip › Figure 5 - figure supplement 1 - source data/source data Figure 5 - figure supplement 1C/IR-pY1146_AKT-pT308.tif]

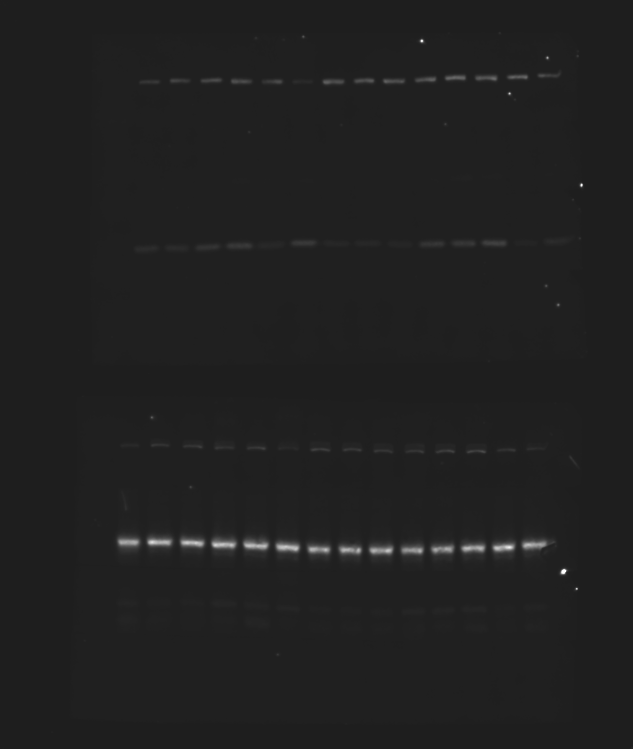

Supplement: Figure 5—figure supplement 1—source data 1. [file elife-85103-fig5-figsupp1-data1.zip › Figure 5 - figure supplement 1 - source data/source data Figure 5 - figure supplement 1C/FASN_AKT-pS473_ACC_IR.tif]

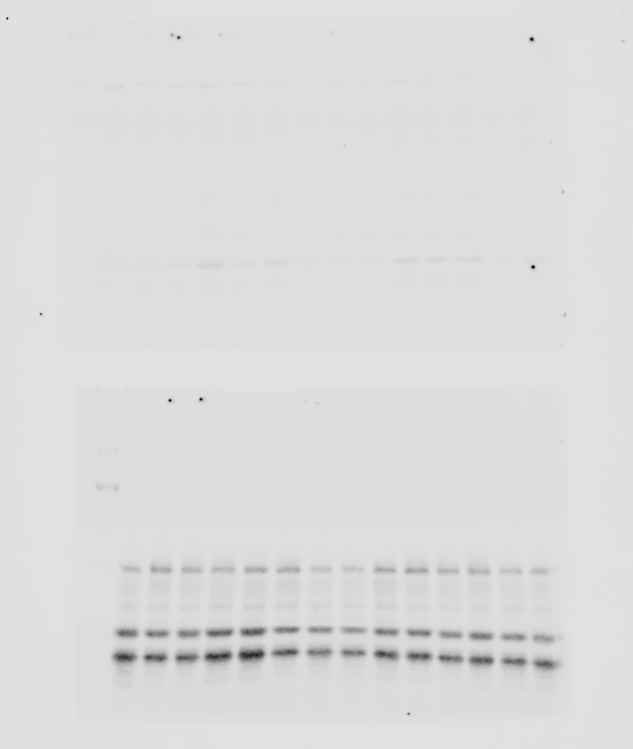

Supplement: Figure 5—figure supplement 1—source data 1. [file elife-85103-fig5-figsupp1-data1.zip › Figure 5 - figure supplement 1 - source data/source data Figure 5 - figure supplement 1C/GSK3-pS21_GSK3.tif]

Figure 5B

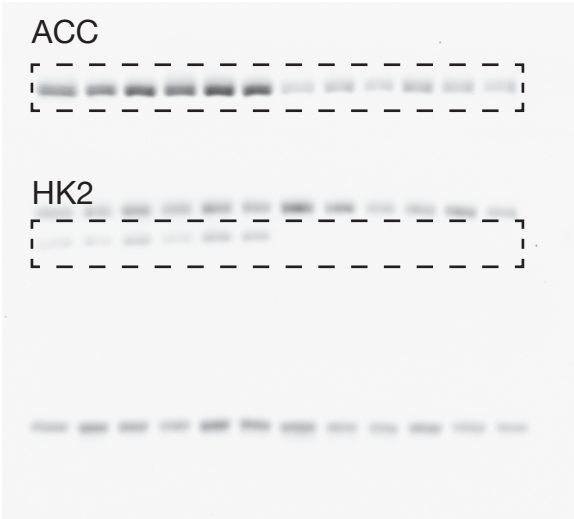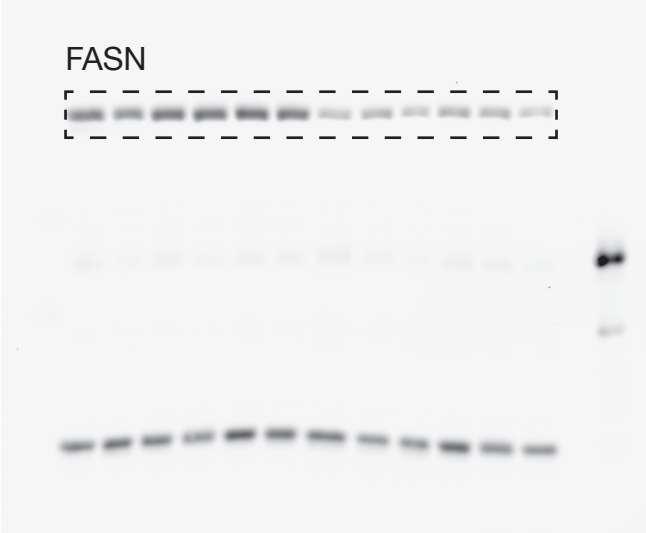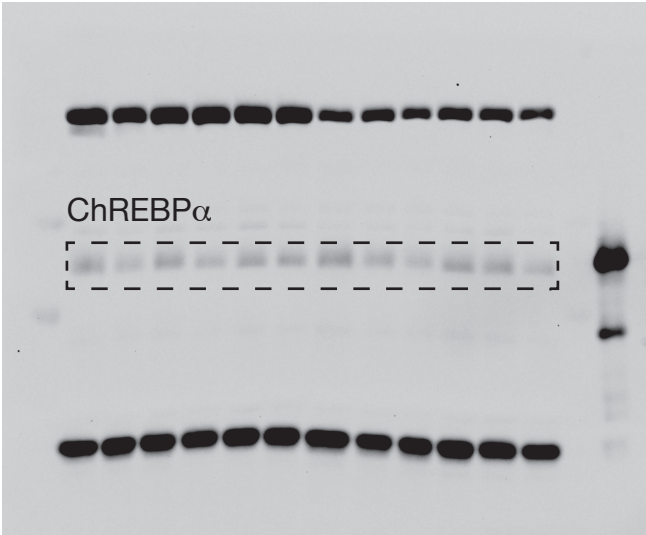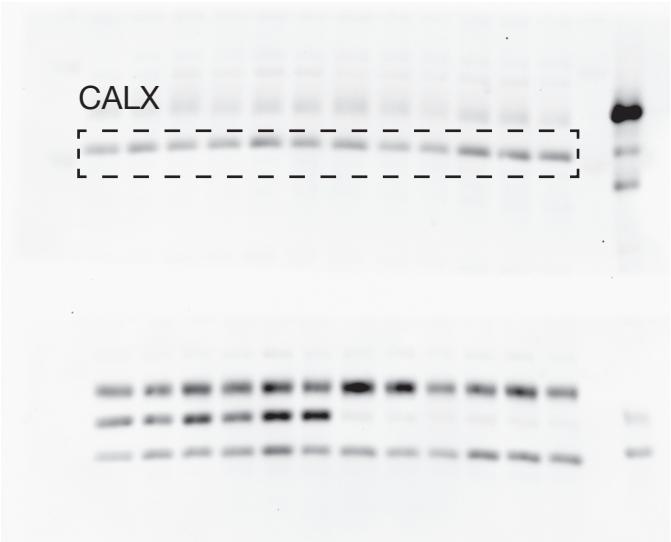

Supplement: Figure 6—source data 1. [file elife-85103-fig6-data1.zip › Figure 6- source data/Figure 6B_source data.pdf]

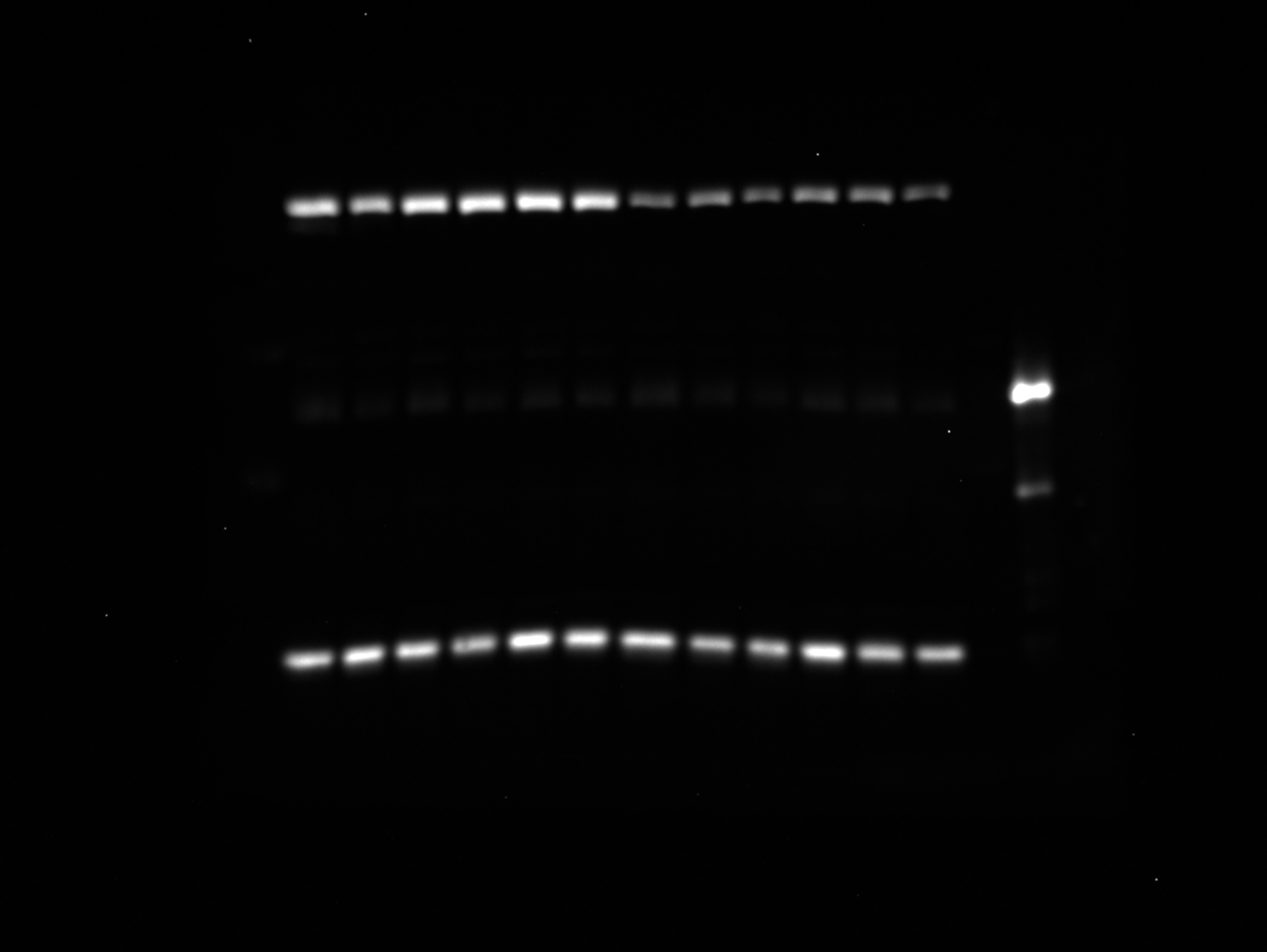

Supplement: Figure 6—source data 1. [file elife-85103-fig6-data1.zip › Figure 6- source data/source data Figure6B/FASN_ChREBP.tif]

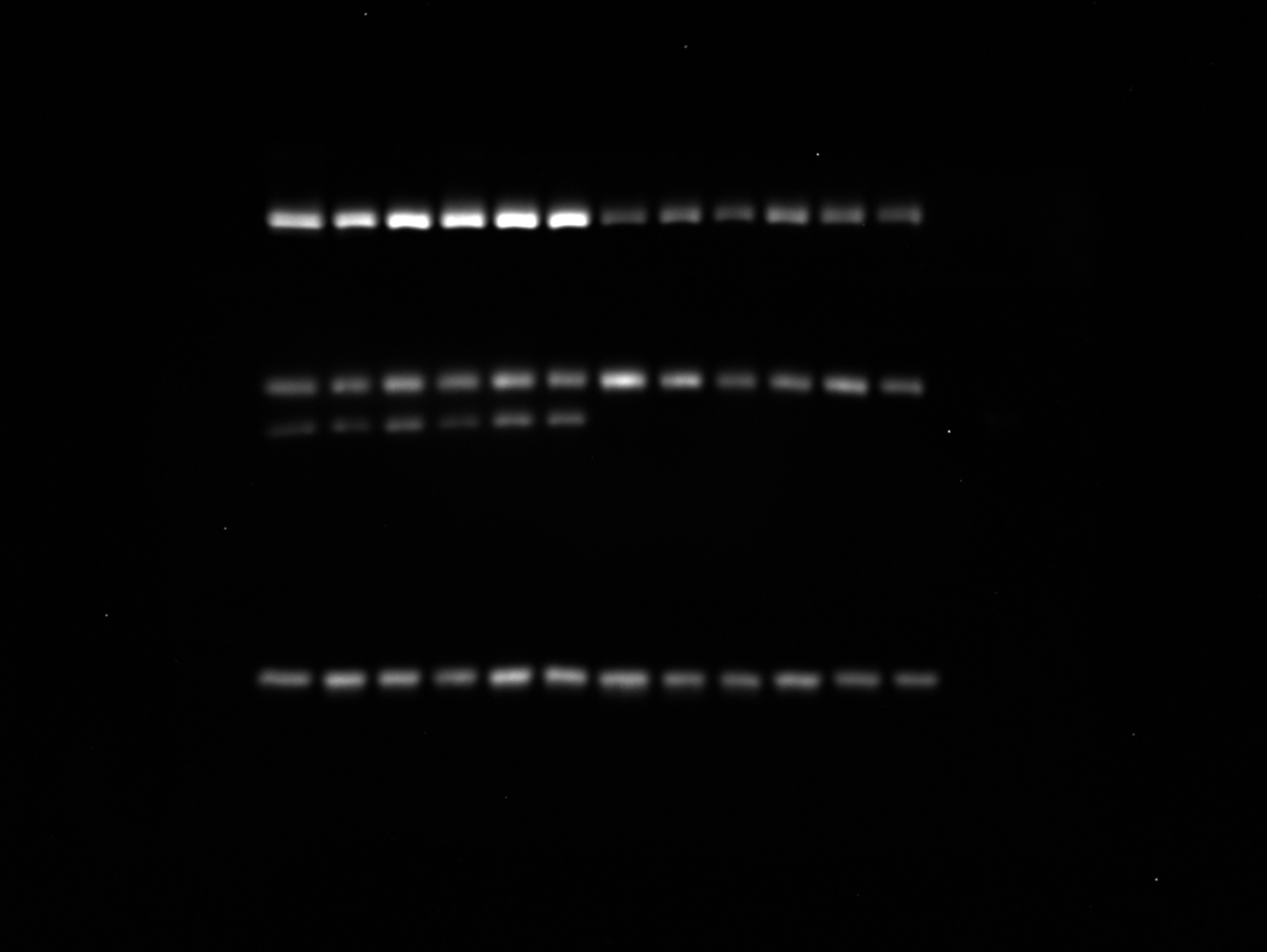

Supplement: Figure 6—source data 1. [file elife-85103-fig6-data1.zip › Figure 6- source data/source data Figure6B/ACC_HK2.tif]

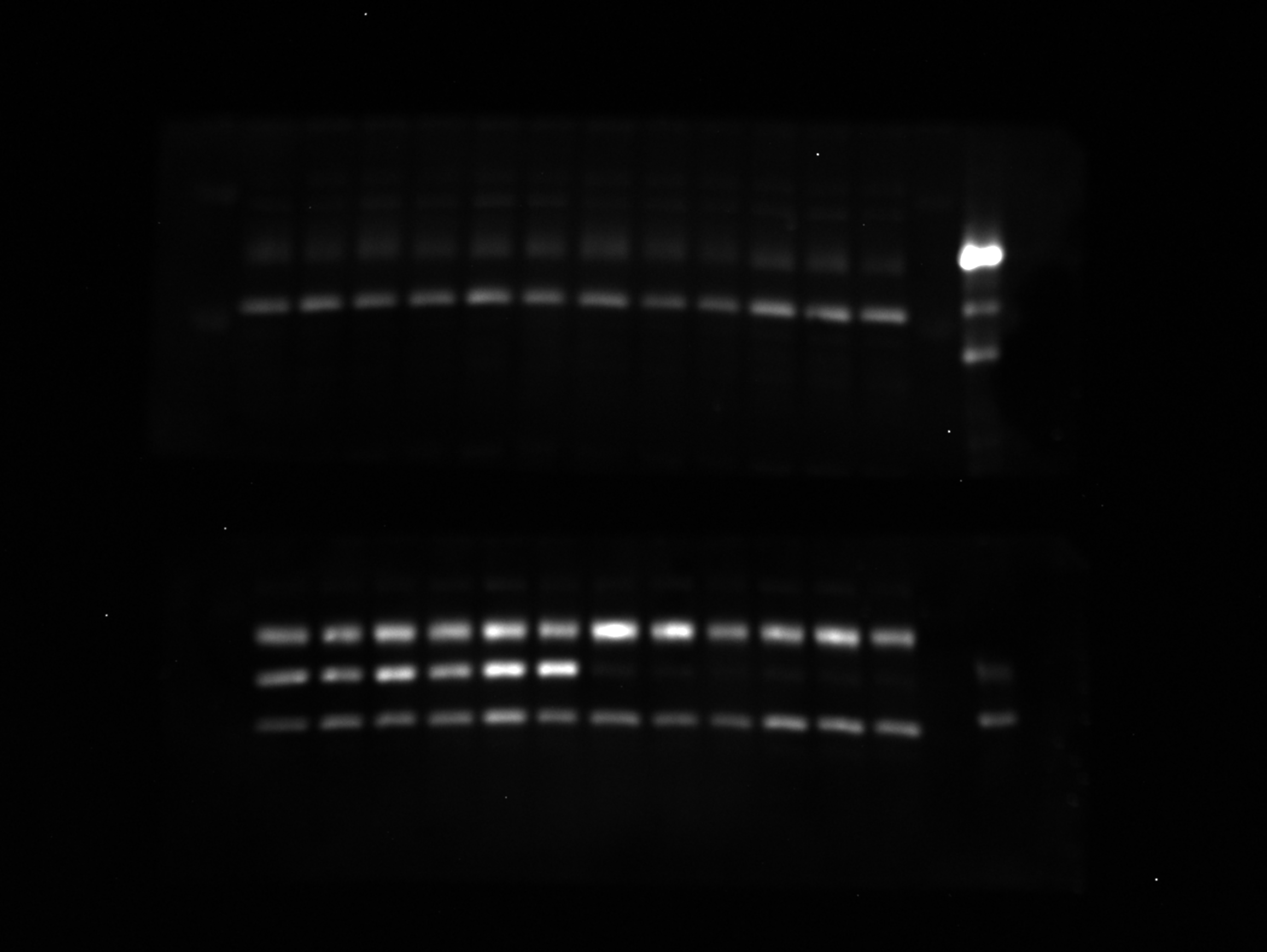

Supplement: Figure 6—source data 1. [file elife-85103-fig6-data1.zip › Figure 6- source data/source data Figure6B/CALX.tif]
