# Supplementary material for: Diet-induced loss of adipose hexokinase 2 correlates with hyperglycemia
Source: eLife. 2023 Mar 15;12:e85103. doi: 10.7554/eLife.85103 (PMC10017106; doi:10.7554/eLife.85103)

Figure 4 - figure supplement 3A vWAT

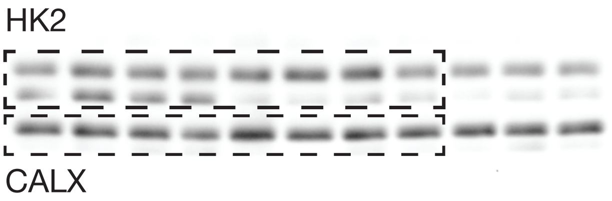

Figure 4 - figure supplement 3A sWAT

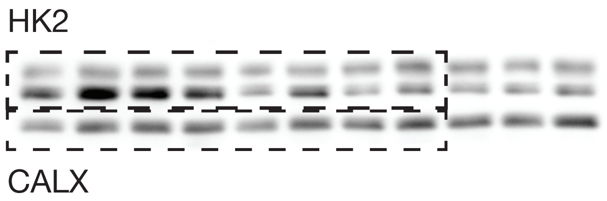

Figure 4 - figure supplement 3A BAT

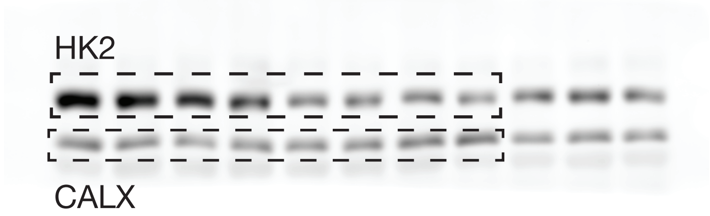

Supplement: Figure 4—figure supplement 3—source data 1. [file elife-85103-fig4-figsupp3-data1.zip › Figure 4 - figure supplement 3 - source data/Figure 4 - figure supplement 3 - source data.pdf]
